# Increased copy number of imprinted genes in the chromosomal region 20q11-q13.32 is associated with resistance to antitumor agents in cancer cell lines

**DOI:** 10.1186/s13148-022-01368-7

**Published:** 2022-12-02

**Authors:** Julia Krushkal, Suleyman Vural, Travis L. Jensen, George Wright, Yingdong Zhao

**Affiliations:** 1 Biometric Research Program, Division of Cancer Treatment and Diagnosis, National Cancer Institute, 9609 Medical Center Dr, Rockville, MD 20850 USA; 2 The Emmes Company, LLC, Rockville, MD 20850 USA; 3 Present Address: Marie-Josee and Henry R. Kravis Center for Molecular Oncology, Memorial Sloan Kettering Cancer Center, 1275 York Avenue, New York, NY 10065 USA

**Keywords:** Imprinting, Copy number, DNA methylation, Epigenetic regulation, Cancer drug treatment

## Abstract

**Background:**

Parent of origin-specific allelic expression of imprinted genes is epigenetically controlled. In cancer, imprinted genes undergo both genomic and epigenomic alterations, including frequent copy number changes. We investigated whether copy number loss or gain of imprinted genes in cancer cell lines is associated with response to chemotherapy treatment.

**Results:**

We analyzed 198 human imprinted genes including protein-coding genes and noncoding RNA genes using data from tumor cell lines from the Cancer Cell Line Encyclopedia and Genomics of Drug Sensitivity in Cancer datasets. We examined whether copy number of the imprinted genes in 35 different genome locations was associated with response to cancer drug treatment. We also analyzed associations of pretreatment expression and DNA methylation of imprinted genes with drug response. Higher copy number of *BLCAP, GNAS, NNAT, GNAS-AS1, HM13, MIR296, MIR298,* and *PSIMCT-1* in the chromosomal region 20q11-q13.32 was associated with resistance to multiple antitumor agents. Increased expression of *BLCAP* and *HM13* was also associated with drug resistance, whereas higher methylation of gene regions of *BLCAP, NNAT, SGK2,* and *GNAS* was associated with drug sensitivity. While expression and methylation of imprinted genes in several other chromosomal regions was also associated with drug response and many imprinted genes in different chromosomal locations showed a considerable copy number variation, only imprinted genes at 20q11-q13.32 had a consistent association of their copy number with drug response. Copy number values among the imprinted genes in the 20q11-q13.32 region were strongly correlated. They were also correlated with the copy number of cancer-related non-imprinted genes *MYBL2, AURKA,* and *ZNF217* in that chromosomal region. Expression of genes at 20q11-q13.32 was associated with ex vivo drug response in primary tumor samples from the Beat AML 1.0 acute myeloid leukemia patient cohort. Association of the increased copy number of the 20q11-q13.32 region with drug resistance may be complex and could involve multiple genes.

**Conclusions:**

Copy number of imprinted and non-imprinted genes in the chromosomal region 20q11-q13.32 was associated with cancer drug resistance. The genes in this chromosomal region may have a modulating effect on tumor response to chemotherapy.

**Supplementary Information:**

The online version contains supplementary material available at 10.1186/s13148-022-01368-7.

## Background

Imprinted genes are characterized by differential allelic expression, which is dependent on the parental origin of the alleles and is closely regulated in normal tissues [1–7]. Many imprinted genes promote body growth, cell metabolism, and cell proliferation in normal embryonic or postnatal development [2, 4, 5, 8–12]. In tumors, imprinted genes undergo multiple genomic and epigenomic alterations including single nucleotide changes, copy number changes, and changes in their DNA methylation and expression [1, 2, 13–21]. Transcriptional upregulation, silencing, and posttranscriptional regulation of imprinted genes have been reported in different cancer categories [2, 20, 22–26]. Some of the epigenetic changes affecting imprinted loci in tumors involve loss or gain of imprinting or an epigenetic switch of allelic expression [13, 14, 19, 24, 27–29].

In cancer, epigenetic dysregulation and genomic changes in protein-coding imprinted loci and in imprinted loci encoding noncoding RNAs modulate tumor cell signaling, differentiation, metabolism, migration, apoptosis, and hormonal regulation and promote tumor growth and cell proliferation [2, 8, 25, 30, 31]. There is also growing evidence that epigenomic and genomic changes affecting some imprinted loci in tumors may affect response to cancer treatment. For example, molecular interactions of the maternally expressed long noncoding RNA transcript *H19* have been linked to resistance of tumor cells to 5-fluorouracil (5-FU) [32]. In cancer stem cells, loss of imprinting of the paternally expressed *IGF2* transcript, colocalized in the same gene cluster and imprinted in the opposite manner from *H19* [33], has been similarly linked to resistance to 5-FU, oxaliplatin, and radiotherapy [34]; however, loss of *IGF2* imprinting has also been associated with improved survival of patients with esophageal cancer [26]. The product of the imprinted organic cation transporter (OCT) gene *OCT2 (SLC22A2)* participates in the uptake of oxaliplatin and its accumulation and cytotoxicity [35]. The product of the *TP73* gene, p73, has been associated with chemosensitivity, and its TA isoforms were upregulated in cancer cell lines by DNA damaging antitumor agents and paclitaxel [36]. PLAGL1 (also known as ZAC1) is involved in androgen receptor signaling in prostate cancer and has been hypothesized to promote castration resistance [2]. The expression of *PEG10* is upregulated by androgen in androgen-dependent hepatocellular carcinoma, whereas the loss of *PEG10* expression blocked the ability of dihydrotestosterone to enhance hepatocellular carcinoma cell growth and apoptotic resistance [2]. Loss of DNA methylation at multiple imprinted loci in hepatocellular carcinoma has been associated with shorter overall patient survival [37]. These and other examples suggest the potential influence of different imprinted loci on response to cancer treatment.

For many imprinted genes, copy number changes frequently occur in tumor cells [14, 38, 39]. Both copy number loss and copy number gains of imprinted genes have been reported, some of which have been specific to individual cancer categories [14, 38, 39]. However, the potential effects of copy number changes of imprinted genes on tumor response to drug treatment have not been examined in depth. To address this question, we performed a comprehensive examination of associations between the response of cancer cell lines to antitumor agents and copy number of human imprinted genes. We used publicly available data from the Cancer Cell Line Encyclopedia (CCLE) and the Genomics of Drug Sensitivity in Cancer (GDSC) [40–45]. We also examined the association of drug response with gene expression and methylation measures of imprinted genes. For validation of the top findings, we analyzed data from tumor samples from the Beat AML 1.0 cohort of patients with acute myeloid leukemia (AML) [46].

## Methods

Additional file 1: Fig. S1 provides an overview of the workflow of the analysis steps in this study.

### Imprinted gene information

Information about human imprinted genes was collected from two comprehensive online resources, the Catalogue of Imprinted Genes [47–49] and Geneimprint [50, 51], and from additional biomedical publications [2, 6, 9, 15, 18, 22, 23, 31, 37, 38, 51–72]. All genes were manually reviewed for the concordance of their imprinted status among different online and literature sources. Due to the abundance of aberrant molecular changes in tumor cells which may involve some patterns that are uncharacteristic for the normal somatic tissues, we included all human genes that had been reported to be imprinted in embryonic or adult somatic tissues, placenta, embryonic stem cells, or induced pluripotent stem cells (iPSCs) [9, 52–54, 58, 68, 70, 73–75] and for which molecular data were available. Those genes whose imprinted status was reported as conflicted among different sources were included if at least two references suggested their imprinting in any human tissue. Additional file 2: Table S1 provides the list of 198 protein-coded imprinted genes and imprinted noncoding RNAs (ncRNAs) which were included in our analysis of their copy number, gene expression, and methylation data, using available molecular information from the CCLE and the GDSC datasets [42–45]. Information about gene name synonyms was obtained from GeneCards [76, 77]. Information about the parental origin of allelic expression of individual genes in non-malignant cells was collected from biomedical literature.

### Cell line drug response data

We used drug response and molecular data for 645 cell lines, the identity of which was matched between the CCLE and the GDSC datasets [40–45]. Below, we refer to the drug response and molecular data for these 645 cell lines as the CCLE-GDSC dataset. The details of cell line identity matching and collection of drug response data were provided in our previous study [78], and the list of 645 matched cell lines is available online [79]. The IC50 measures of drug response, representing the total drug concentration that reduced cell activity by 50%, were available for 24 agents from CCLE [40, 41, 45] and 251 agents from GDSC (GDSC1 dataset, to which we refer as GDSC measures) [42, 44, 80]. All drug response values were transformed to the log_10_(IC50) scale. Cell line identities in the CCLE and GDSC datasets and their cancer categories were verified using Cellosaurus [81]. Response measures for 11 agents which were present in both CCLE and GDSC data were analyzed separately, without combining the CCLE and GDSC measures. For the agents in the GDSC dataset that had duplicate measurements [44], we used the combined average of their drug response measures from separate experiments. The resulting dataset included 275 CCLE and GDSC drug response measures for 255 antitumor agents. The concordance of drug response measures between the CCLE and GDSC datasets has been reported previously [82–84]. Information about molecular drug targets of individual agents was obtained from the GDSC data download site [80] and from biomedical literature.

### Cell line copy number data

Imprinted genes included in the copy number analysis and their chromosomal locations are listed in Additional file 3: Table S2. Copy number data for imprinted genes were obtained from the CCLE legacy portal [40, 41, 45]. At present time, both legacy and most recent CCLE copy number data are available from the Cancer Dependency Map (DepMap) project site [45, 85, 86]. We analyzed gene copy number of 623 cell lines, which were a part of the 645 cell line dataset with available methylation, gene expression, and drug response data. Gene-level copy number data had been generated by the CCLE Consortium using Affymetrix 6.0 Genome-Wide Human SNP arrays, with segmentation of normalized log_2_ ratios of the copy number estimates performed using the circular binary segmentation algorithm [40, 41]. These continuous copy number values downloaded from CCLE were used in the association analyses with drug response and in the analyses of correlation with methylation and expression of imprinted genes. For visualization of the copy number of imprinted genes, these continuous values were transformed to gene-level discreet copy number estimates, rounded to the nearest integer, and plotted on a separate histogram for each imprinted gene (Additional file 4: Fig. S2). Quality control of the copy number data had been described by the CCLE consortium [40].

Chromosomal location of the imprinted genes was identified according to the information in the Catalog of Imprinted Genes [47–49], Bonaldi et al. [52], and biomedical literature. For those imprinted genes that had minor discrepancies among their reported chromosomal location from different sources, their chromosomal location was reported according to GeneCards [76, 77].

### Cell line RNA-seq gene expression data

RPKM expression values of 108 imprinted genes which also had copy number data (Additional file 2: Table S1) were downloaded from the CCLE legacy portal of the Broad Institute [45, 87]. Both legacy and most recent CCLE expression data are now available from the Cancer Dependency Map (DepMap) project site [45, 85, 86]. RNA sample library preparation using Illumina TruSeq RNA Sample Preparation protocol, RNA sequencing using Illumina HiSeq 2000 and HiSeq 2500, and initial data processing were previously described by the CCLE project [88].

### Cell line gene region-averaged DNA methylation data

Methylation data for imprinted genes were obtained from the epigenome-wide dataset generated by the GDSC project [44] using Illumina Infinium HumanMethylation450 (450K) BeadChip array (Illumina, Inc.). QC and filtering of these data were described in our previous report [78]. Methylation data were downloaded from the National Center for Biotechnology Information Gene Expression Omnibus (NCBI GEO) [89]. Methylation probe beta values for individual cell lines with detection *p *values ≥ 10^–3^ and the entire probes with median detection *p *values ≥ 10^–6^ were excluded. Probes overlapping with single nucleotide polymorphisms were also filtered out, based on the probe masking recommendations for hg19 (GRCh37) [90, 91]. The resulting methylation dataset included methylation beta values for 5808 probes that passed all filtering and were annotated as being part of 113 imprinted genes that also had copy number data. Among them, 98 genes had methylation, expression, and copy number data (Additional file 2: Table S1).

We combined the methylation probes that passed the filtering to compute gene region-averaged methylation beta values as described in our earlier report [78]. We used the Illumina Infinium HumanMethylation450 BeadChip annotation of each probe [92] according to the UCSC genome browser to compute average methylation for 6 gene regions: TSS1500 (200–1500 bases upstream of the transcriptional start site, or TSS), TSS200 (0–200 bases upstream of the TSS), 5′ UTR (the 5′ untranslated region between the TSS and the ATG start site), 1st exon, gene body (the region between the ATG start site and the stop codon), and 3' UTR (the 3' untranslated region between the stop codon and poly A signal). We refer to these 6 gene fragments as gene regions (as opposed to chromosomal regions, which denote cytogenetic locations). The gene region-averaged methylation values were computed for 515 gene regions in 113 imprinted genes, with each gene represented by up to 6 gene regions. We examined association of methylation of each gene region with drug response. Chromosomal regions (cytobands) of individual gene regions were identified according to the UCSC genome annotation database for the hg19 (GRCh37) assembly of the human genome based on the probe coordinates in the Illumina Infinium HumanMethylation 450K BeadChip annotation. This annotation of locations of individual gene regions was in full agreement with the cytoband locations of their respective genes according to GeneCards [76, 77], which are reported in Additional file 2: Table S1.

### Association analysis of copy number, methylation, and expression of imprinted genes with drug response in the CCLE-GDSC dataset

Association of copy number, expression, and DNA methylation of imprinted genes with log(IC50) was examined using Spearman and Pearson correlation analyses. We chose to employ correlation analysis to examine their associations, since all variables were continuously distributed. Significance of the associations was evaluated using the Benjamini–Hochberg adjustment procedure for false discovery rate (FDR) [17]. The associations with FDR-adjusted *p* < 0.05 were considered statistically significant. In addition to our focus only on statistically significant associations, we also used the amplitude of the correlation coefficient as a measure of effect size of the correlations, and we primarily focused on the associations with the absolute value of the Spearman correlation coefficient |*ρ*| > 0.3.

Association analyses were performed in the combined dataset of different cancer categories (pancancer analysis of all 645 cell lines), and also separately within each cancer category with ≥ 10 cell lines. There were 22 tumor types with ≥ 10 cell lines in the analysis of copy number data and 23 such cancer categories for expression and methylation data. While we analyzed many cancer categories based on the Cancer Genome Atlas (TCGA) definitions, some cancer types from the same organ were grouped into broader categories, resulting in the inclusion of a broader range of similar cell lines than those defined by TCGA [78]. Additional categories not presented in TCGA (e.g., small cell lung cancer, neuroblastoma, and others) were also analyzed. In the FDR adjustment of the results of the analyses stratified by individual cancer categories, we accounted for all cancer types used in these analyses.

Adjustment for multiple testing in each analysis accounted for all 275 drug response measures analyzed. Adjustment for multiple testing of the associations of methylation and gene expression data with log(IC50) accounted for all imprinted genes included in these analyses, treating these genes as independent from each other. When analyzing the association of copy number data of the imprinted genes with drug response, we used chromosomal segment-based gene grouping [93] to account for non-independence of the copy number values of the genes located in close proximity to each other in the same imprinted clusters or in the adjacent chromosomal regions. We assigned the imprinted genes to 35 segments (bins), based on their chromosomal locations (Additional file 3: Table S2). All imprinted genes located in the same cytogenetic region were assigned to the same segment. Imprinted genes located in the adjacent cytogenetic regions were assigned to the same segment if evidence had been reported for their frequent joint copy number loss or gain in germline, somatic, and/or tumor cells. For example, the imprinted genes in the Prader–Willi syndrome locus 15q11-q13, the genes in the *DLK1-DIO3* cluster at 14q32.2-q32.31, and the imprinted genes in the 20q11-q13.32 region were grouped in their respective segments, separate for each of these three chromosomal regions [22, 57, 94–96]. We also assigned imprinted genes in the adjacent cytogenetic regions to the same segment if their copy number values were strongly correlated with each other (Pearson correlation coefficient *r* > 0.7 in the CCLE data according to CellMinerCDB v. 1.3 [97, 98]), which resulted in our combining the genes in the 6q24.2-q25.3 region in one segment and the genes on 8q24.22-q24.3 in another segment. When adjusting the *p *values resulting from copy number analysis for FDR, for each specific agent and cancer category (if performing a stratified analysis among tumor types), we used a conservative approach in which the highest *p* value among all genes in each segment was assigned to that segment and was used for FDR adjustment, with each of the 35 segments represented once. All genes within each segment were assigned the same FDR-adjusted *p* value for that segment for association with a given agent. We also accounted for the tumor category in the tumor-specific analysis of copy number data. We refer to the *p *values prior to FDR adjustment as *p*_0_*,* FDR-adjusted *p *values using segment-based gene grouping based on the maximum *p *value in each segment as *p*_SegmFDR_*,* and FDR-adjusted *p* values when treating each gene independently as *p*_FDR_. All copy number association results presented in this report which satisfied *p*_SegmFDR_ < 0.05 also satisfied FDR-adjusted *p* < 0.05 if considering the imprinted genes independently or using the lowest *p *value among the imprinted genes in each segment (data not shown).

For the top genes whose copy number, expression, or DNA methylation were associated with drug response, we also examined Spearman and Pearson correlation among these molecular measures. Analyses and graphical presentation of the results were performed using Python v. 2.7.17 and R v. 3.6.3, and RStudio v. 1.2.5033 and 1.4.1103.

### Follow-up association analysis of non-imprinted genes *MYBL2*, *AURKA*, and *ZNF217* at 20q11-q13.32

The objective of our study was to investigate the association of copy number, expression, and methylation of imprinted genes with drug response. After our analysis of imprinted genes in different parts of the genome showed a consistent and significant association of molecular measures for several imprinted genes in the chromosomal region 20q11-q13.32 with drug resistance, we performed an additional Spearman and Pearson correlation analysis to evaluate a possible influence of three non-imprinted cancer-related genes, *MYBL2, AURKA,* and *ZNF217,* located in the same chromosomal region (Additional file 1: Fig. S1). This follow-up analysis examined their effect as potential confounders of the association of molecular measures of imprinted genes at 20q11-q13.32 with drug response.

### Validation of associations of gene expression in the 20q11-q13.32 region with drug resistance using data from the Beat AML 1.0 cohort

After observing that molecular features of genes at 20q11-q13.2 in our pancancer cell line analysis were associated with multiple agents used in the treatment of hematological malignancies [99, 100], we validated these findings by analyzing publicly available gene expression data, ex vivo drug response measures, and patient survival data from the Beat AML 1.0 cohort of patients with acute myeloid leukemia [46]. This dataset represents the first two waves of patient and data accrual by the Beat AML study [101].

The log_2_-transformed RPKM gene expression measures, which had been generated by the Beat AML study using RNA sequencing for 451 specimens derived from 411 patients [46], were downloaded from cBioPortal [102, 103]. Such expression data were available for the imprinted genes *BLCAP, HM13, GNAS, NNAT, L3MBTL1*, and *SGK2* and the non-imprinted genes *MYBL2, AURKA*, and *ZNF217*. Ex vivo IC50 drug response measures for 409 primary tumor specimens (freshly isolated mononuclear cell populations) from 363 AML patients, patient survival data (in days), clinical and cytogenetic information (including cytogenetic data indicating the presence of the loss of the entire 20q or its subregions which are a part of 20q11-q13.32), and *ASXL1* mutation status were downloaded from Additional file 8: Tables S5 and S10 of the Beat AML 1.0 publication [46]. Among the 122 small-molecule inhibitors screened by the Beat AML 1.0 study, drug response data were available for the following agents which were associated with copy number of genes at 20q11-q13.32 in the CCLE-GDSC dataset: axitinib (AG-013736), imatinib, masitinib (AB-1010), TG101348 (fedratinib), GW-2580, lenalidomide, lestaurtinib (CEP-701), linifanib (ABT-869), nilotinib, panobinostat, quizartinib (AC220), ruxolitinib (INCB018424), and tivozanib (AV-951). After applying the log_10_-transformation, these ex vivo IC50 measures were used to analyze Spearman and Pearson correlation with log_2_-transformed RPKM expression data of tumor samples of imprinted and non-imprinted genes at 20q11-q13.32, using all available tumor specimens with IC50 measures for a given agent and expression measures for a given gene. We further assessed whether log_2_-transformed RPKM expression of imprinted and non-imprinted genes was associated with overall patient survival, using the coxph function of the R survival package. The *p* values from correlation and survival analyses of expression of genes at 20q11-q13.32 were adjusted for multiple testing, using all results for each of the 9 genes and each of the 13 agents in the FDR adjustment. Separate FDR adjustments were performed for the results of Spearman, Pearson, and survival analyses. We also used the log-rank test to examine whether available cytogenetic data on the loss of 20q11-q13.32 (deletion of the entire chromosome 20, 20q arm deletion, or loss of a small cytogenetic region within 20q11-q13.32) were associated with overall survival of Beat AML patients [46].

## Results

### Distribution of the copy number values and gene region-averaged methylation values of imprinted genes in the CCLE-GDSC dataset

The range and the median values of rounded copy number of imprinted genes in the 623 cell lines with available copy number data are shown in Additional file 5: Table S3. The distribution of the rounded copy number values is presented in Additional file 4: Fig. S2. While the median copy number of each imprinted gene was 2, some tumor cells had copy number gain and others lost one or both copies of certain imprinted genes. Each of the 198 imprinted genes had a loss of one or both copies in at least one cell line. The largest range of copy number values was observed for *ANO1,* which ranged from the loss of both copies in the CCK81 cell line derived from a metastasis of colorectal cancer to an estimated 17 copies in the FADU hypopharyngeal carcinoma cell line [81]. A number of other cell lines from different tumor types also had high-level *ANO1* amplifications (data not shown), consistent with frequent amplifications of *ANO1* in head and neck squamous carcinoma (HNSCC), bladder, and breast cancers as part of the amplification of the chromosomal region 11q13 [104–106]. Several other imprinted genes also had high-level amplifications in individual cell lines. Their examples include 15 copies of *NTM1,* 14 copies each of *ZFAT*, *PSIMCT-1*, and *AIM1,* and 12 copies of *GLIS3,* as well as 11 or fewer copies of multiple other imprinted genes*.* Consistent with an earlier study [14] which analyzed copy number data generated by the COSMIC cell line project of the Sanger Institute, we observed 14 copies of *HM13* in the SKLU1 cell line in the CCLE copy number data.

Additional file 6: Fig. S3 shows the combined distribution of gene-averaged methylation beta values among 515 imprinted gene regions and separate distribution plots for each imprinted gene region category in 645 cell lines from all cancer categories combined. Among the 6 gene regions, the imprinted status, corresponding to a distribution peak around methylation beta values of 0.5, which would be characteristic of hemimethylated regions that would potentially represent imprinted sites [52, 107–109], was observed for the TSS200 and 5′ UTR gene regions located upstream of imprinted genes, and the first exons of imprinted genes. This location of hemimethylated gene regions is consistent with the previously established overlap between differentially methylated regions (DMR) and promoter regions of imprinted genes [110]. Additional peaks for the three gene regions TSS200, 5′ UTR, and 1st exon showed that some of them were not imprinted in individual genes and cell lines, having low (close to 0) and high (close to 1) methylation beta values. The upstream region TSS1500 showed the presence of multiple intermediate methylation values between 0 and 1. In contrast to these findings for imprinted genes, in an earlier study [78] of this 645 CCLE-GDSC cell line dataset, we examined the distribution of gene region-averaged methylation values among all 93,591 gene regions of all annotated 20,643 genes and ncRNA, the overwhelming majority of which were non-imprinted. Consistent with earlier reports by multiple authors (e.g., [111, 112]), most gene regions in the combined dataset, which was dominated by the gene regions of non-imprinted genes, had the peaks of their average methylation beta values near 0 and 1. The peaks at 0.5 in the TSS200 and 5′ UTR gene regions of the imprinted genes (Additional file 6: Fig. S3), corresponding to hemimethylated sites, were nearly absent from the combined distribution of values when a large number of non-imprinted genes were included [78].

### Association of the copy number of imprinted genes in tumor cell lines with drug response

**Fig. 1:**
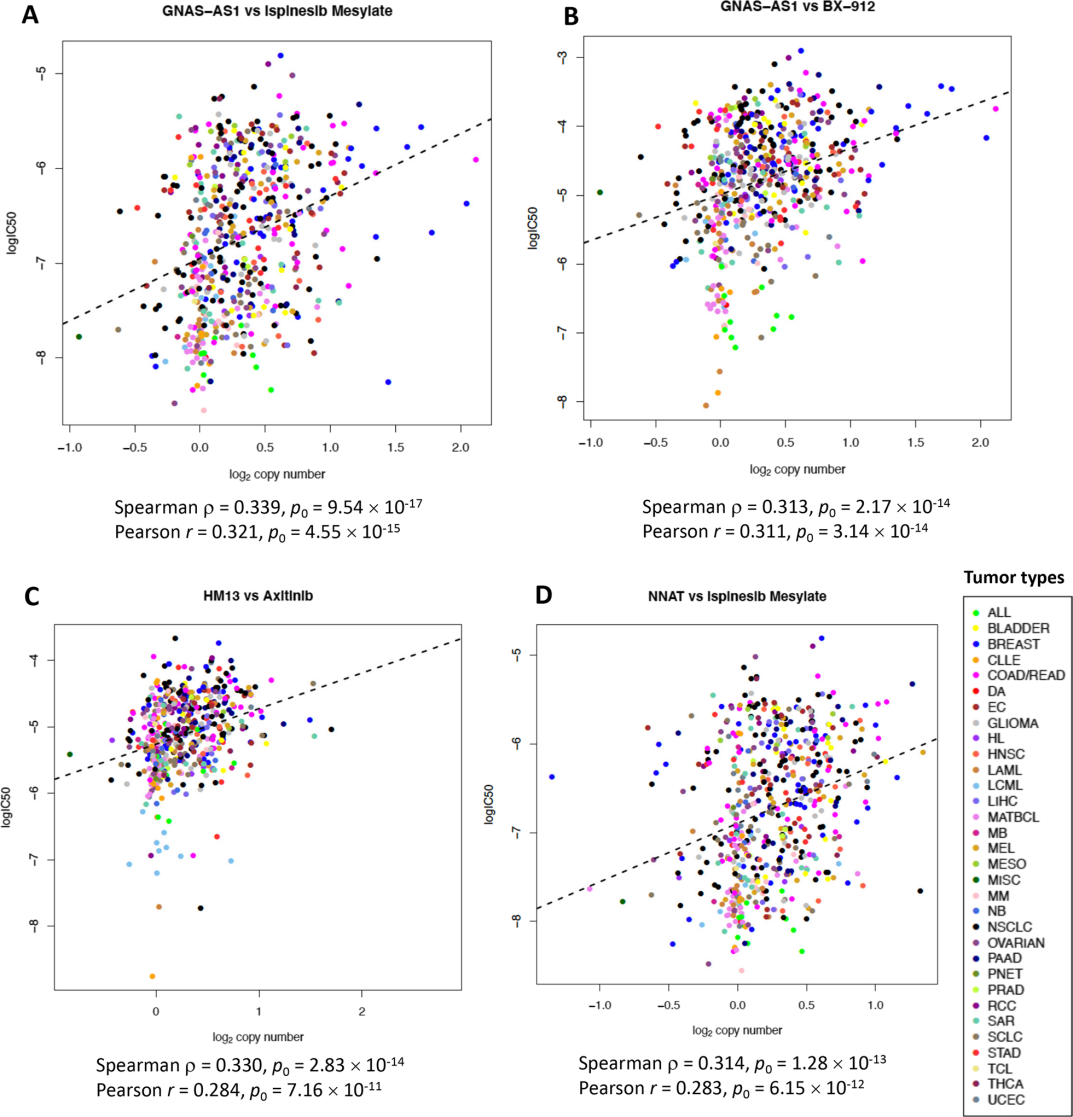
Example scatterplots of the copy number of selected imprinted genes at 20q11-q13.32 versus log(IC50) of antitumor agents listed in Table 1. The dashed line indicates the linear regression line. The list of cancer categories is provided under Abbreviations. *ρ*, Spearman correlation coefficient; *r*, Pearson correlation coefficient. **A** Copy number of *GNAS-AS1* (located at 20q13.32) versus response to ispinesib mesylate. **B** Copy number of *GNAS-AS1* versus response to BX-912. **C** Copy number of *HM13* (at 20q11.21) versus response to axitinib. **D** Copy number of *NNAT* (at 20q11.23) versus response to ispinesib mesylate

Analysis of continuous copy number values of the imprinted genes with log(IC50) in the pancancer dataset showed a modest (Spearman *ρ* > 0.3) statistically significant correlation (*p*_SegmFDR_ < 0.05) of gene copy number values in the imprinted region at 20q11-q13.32 with resistance to multiple agents, which included several kinase inhibitors and other categories of antitumor agents (Table 1, Fig. 1). Higher copy number values of the imprinted protein-coding genes *BLCAP, GNAS, HM13,* and *NNAT,* the ncRNA genes *GNAS-AS1, MIR296,* and *MIR298,* and the imprinted pseudogene *PSIMCT-1* were associated with resistance to 15 antitumor agents including the VEGFR inhibitor axitinib, the PDK1 inhibitor BX-912, the Aurora B/C kinase inhibitor GSK1070916, the Rock inhibitor GSK429286A, the BCR/ABL inhibitor imatinib, the *c-*kit inhibitor masitinib, the CRAF inhibitor TL-2-105, the RIPK1 inhibitor XMD13-2, the ALK/CDK7 inhibitor XMD14-99, the small-molecule kinase inhibitor QL-XI-9, ispinesib mesylate inhibiting the kinesin spindle protein (KSP), *S*-trityl-L-cysteine inhibiting the kinesin related motor protein Eg5, the liver X receptor (LXR) agonist T0901317, the Sonic Hedgehog (Shh) pathway inhibitor cyclopamine, and UNC1215 which acts as an agonist of the epigenetic factor L3MBTL3.

**Table 1:** Results of Spearman correlation analysis of continuous copy number values of the imprinted genes with log(IC50) satisfying *p*_SegmFDR_ < 0.05 and Spearman |*ρ*|> 0.3

Gene	Agent	Sample size	Spearman *ρ*	*p*_0_	Gene category	Location	*p*_SegmFDR_
*MIR298*	Ispinesib mesylate	567	0.3393	9.54 × 10^–17^	ncRNA	20q13.32	3.74 × 10^–8^
*GNAS-AS1*	Ispinesib mesylate	567	0.3393	9.54 × 10^–17^	ncRNA	20q13.32	3.74 × 10^–8^
*MIR296*	Ispinesib mesylate	567	0.3393	9.54 × 10^–17^	ncRNA	20q13.32	3.74 × 10^–8^
*GNAS*	Ispinesib mesylate	567	0.3390	1.03 × 10^–16^	Protein-coding	20q13.32	3.74 × 10^–8^
*MIR298*	T0901317	565	0.3333	4.03 × 10^–16^	ncRNA	20q13.32	9.08 × 10^–7^
*GNAS-AS1*	T0901317	565	0.3333	4.03 × 10^–16^	ncRNA	20q13.32	9.08 × 10^–7^
*MIR296*	T0901317	565	0.3333	4.03 × 10^–16^	ncRNA	20q13.32	9.08 × 10^–7^
*GNAS*	T0901317	565	0.3315	5.83 × 10^–16^	Protein-coding	20q13.32	9.08 × 10^–7^
*PSIMCT-1*	Axitinib	506	0.3295	2.83 × 10^–14^	Pseudogene	20q11.21	2.48 × 10^–6^
*HM13*	Axitinib	506	0.3295	2.83 × 10^–14^	Protein-coding	20q11.21	2.48 × 10^–6^
*HM13*	T0901317	565	0.3239	2.86 × 10^–15^	Protein-coding	20q11.21	9.08 × 10^–7^
*PSIMCT-1*	T0901317	565	0.3239	2.90 × 10^–15^	Pseudogene	20q11.21	9.08 × 10^–7^
*MIR298*	XMD14-99	569	0.3172	9.10 × 10^–15^	ncRNA	20q13.32	3.18 × 10^–6^
*GNAS-AS1*	XMD14-99	569	0.3172	9.10 × 10^–15^	ncRNA	20q13.32	3.18 × 10^–6^
*MIR296*	XMD14-99	569	0.3172	9.10 × 10^–15^	ncRNA	20q13.32	3.18 × 10^–6^
*HM13*	Imatinib	227	0.3150	1.27 × 10^–6^	Protein-coding	20q11.21	0.01754
*PSIMCT-1*	Imatinib	227	0.3150	1.27 × 10^–6^	Pseudogene	20q11.21	0.01754
*GNAS*	XMD14-99	569	0.3150	1.43 × 10^–14^	Protein-coding	20q13.32	3.18 × 10^–6^
*MIR298*	Cyclopamine	217	0.3149	2.22 × 10^–6^	ncRNA	20q13.32	0.01338
*GNAS-AS1*	Cyclopamine	217	0.3149	2.22 × 10^–6^	ncRNA	20q13.32	0.01338
*MIR296*	Cyclopamine	217	0.3149	2.22 × 10^–6^	ncRNA	20q13.32	0.01338
*MIR298*	BX-912	569	0.3129	2.17 × 10^–14^	ncRNA	20q13.32	3.87 × 10^–6^
*GNAS-AS1*	BX-912	569	0.3129	2.17 × 10^–14^	ncRNA	20q13.32	3.87 × 10^–6^
*MIR296*	BX-912	569	0.3129	2.17 × 10^–14^	ncRNA	20q13.32	3.87 × 10^–6^
*GNAS*	XMD13-2	569	0.3120	2.62 × 10^–14^	Protein-coding	20q13.32	2.48 × 10^–6^
*GNAS*	BX-912	569	0.3114	2.90 × 10^–14^	Protein-coding	20q13.32	3.87 × 10^–6^
*GNAS*	Cyclopamine	217	0.3109	3.01 × 10^–6^	Protein-coding	20q13.32	0.01338
*HM13*	S-Trityl-L-cysteine	221	0.3108	2.46 × 10^–6^	Protein-coding	20q11.21	0.00495
*PSIMCT-1*	S-Trityl-L-cysteine	221	0.3108	2.46 × 10^–6^	Pseudogene	20q11.21	0.00495
*GNAS*	QL-XI-92	569	0.3106	3.41 × 10^–14^	Protein-coding	20q13.32	1.57 × 10^–6^
*MIR298*	XMD13-2	569	0.3093	4.41 × 10^–14^	ncRNA	20q13.32	2.48 × 10^–6^
*GNAS-AS1*	XMD13-2	569	0.3093	4.41 × 10^–14^	ncRNA	20q13.32	2.48 × 10^–6^
*MIR296*	XMD13-2	569	0.3093	4.41 × 10^–14^	ncRNA	20q13.32	2.48 × 10^–6^
*MIR298*	QL-XI-92	569	0.3092	4.53 × 10^–14^	ncRNA	20q13.32	1.57 × 10^–6^
*GNAS-AS1*	QL-XI-92	569	0.3092	4.53 × 10^–14^	ncRNA	20q13.32	1.57 × 10^–6^
*MIR296*	QL-XI-92	569	0.3092	4.53 × 10^–14^	ncRNA	20q13.3	1.57 × 10^–6^
*MIR298*	GSK1070916	555	0.3072	1.37 × 10^–13^	ncRNA	20q13.32	2.76 × 10^–6^
*GNAS-AS1*	GSK1070916	555	0.3072	1.37 × 10^–13^	ncRNA	20q13.32	2.76 × 10^–6^
*MIR296*	GSK1070916	555	0.3072	1.37 × 10^–13^	ncRNA	20q13.32	2.76 × 10^–6^
*GNAS*	GSK1070916	555	0.3057	1.81 × 10^–13^	Protein-coding	20q13.32	2.76 × 10^–6^
*MIR298*	UNC1215	553	0.3054	2.13 × 10^–13^	ncRNA	20q13.32	5.28 × 10^–6^
*GNAS-AS1*	UNC1215	553	0.3054	2.13 × 10^–13^	ncRNA	20q13.32	5.28 × 10^–6^
*MIR296*	UNC1215	553	0.3054	2.13 × 10^–13^	ncRNA	20q13.32	5.28 × 10^–6^
*MIR298*	GSK429286A	569	0.3052	9.81 × 10^–14^	ncRNA	20q13.32	1.50 × 10^–6^
*GNAS-AS1*	GSK429286A	569	0.3052	9.81 × 10^–14^	ncRNA	20q13.32	1.50 × 10^–6^
*MIR296*	GSK429286A	569	0.3052	9.81 × 10^–14^	ncRNA	20q13.32	1.50 × 10^–6^
*BLCAP*	Ispinesib mesylate	567	0.3044	1.28 × 10^–13^	Protein-coding	20q11.23	3.74 × 10^–8^
*NNAT*	Ispinesib mesylate	567	0.3044	1.28 × 10^–13^	Protein-coding	20q11.23	3.74 × 10^–8^
*GNAS*	UNC1215	553	0.3037	2.91 × 10^–13^	Protein-coding	20q13.32	5.28 × 10^–6^
*HM13*	TL-2–105	569	0.3034	1.40 × 10^–13^	Protein-coding	20q11.21	1.34 × 10^–7^
*GNAS*	GSK429286A	569	0.3028	1.59 × 10^–13^	Protein-coding	20q13.32	1.50 × 10^–6^
*GNAS*	Masitinib	568	0.3023	1.81 × 10^–13^	Protein-coding	20q13.32	2.31 × 10^–6^
*PSIMCT-1*	TL-2–105	569	0.3021	1.79 × 10^–13^	Pseudogene	20q11.21	1.34 × 10^–7^
*PSIMCT-1*	UNC1215	553	0.3019	4.05 × 10^–13^	Pseudogene	20q11.21	5.28 × 10^–6^
*HM13*	Ispinesib mesylate	567	0.3004	2.74 × 10^–13^	Protein-coding	20q11.21	3.74 × 10^–8^

*Sample size* number of cell lines with available data used in correlation analysis; *Spearman ρ* Spearman correlation coefficient; *p*_0_ *p* value prior to FDR adjustment; *p*_SegmFDR_ *p* value after FDR adjustment using the maximal *p* values from each chromosomal segment

Segment assignment of all imprinted genes is provided in Additional file 3: Table S2. All correlations presented in the table also satisfied FDR-adjusted *p* < 0.05 if considering all genes independently, without grouping them into segments (*p*_FDR_ < 0.05). All agents listed in the table were from the GDSC dataset

Additional file 7: Table S4 provides an expanded list of associations with a variety of antitumor agents, satisfying *p*_SegmFDR_ < 0.05 and a weak threshold for the Spearman correlation coefficient, |*ρ*| > 0.25. The majority of weak associations included genes at 20q11-q13.32 and involved agents with different mechanisms of action. In addition to kinase inhibitors, examples of weakly associated agents included epigenetic agents such as tubastatin A, vorinostat, panobinostat, selisistat, PFI-3, and SGC0946, various DNA damaging agents, e.g., topotecan, CX-5461, veliparib, and temozolomide, and other agents from separate categories of antitumor drugs. In addition to the imprinted genes listed in Table 1, *L*3*MBTL1 (L3MBTL), SGK2,* and *GDAP1L1* in the chromosomal region at 20q11-q13.32 had weak statistically significant associations of their copy number with resistance to antitumor agents which did not reach *ρ* > 0.3. For example, *L3MBTL1* and *SGK2* both had *ρ* = 0.286 and *p*_SegmFDR_ = 3.74 × 10^–8^ for associations with resistance to ispinesib mesylate.

*BLCAP* and *NNAT* each had between 1 and 5 copies in different cell lines (Additional file 5: Table S3 and Additional file 4: Fig. S2). These genes have an overlapping genome location, with *NNAT* encoded in an antisense orientation within an intron of *BLCAP.* The complex *GNAS* locus at 20q13.32, which encodes multiple transcripts [64], was represented in the CCLE-GDSC dataset by the summary gene-level measures for *GNAS*, the ncRNA transcript *GNAS-AS1,* and miRNAs *MIR296* and *MIR298*. Copy number values of *GNAS* and *GNAS-AS1* ranged between 1 and 9. Only 5 out of 623, or 0.8% of the cell lines in the CCLE-GDSC dataset had one copy of *GNAS* based on the rounded values, suggesting a loss of one copy of that gene. These cell lines were from the NSCLC (HCC15 and LXF289), gastric adenocarcinoma (GCIY), SCLC (NCI-H69), and Hodgkin lymphoma (HDLM2) tumors of origin. In contrast, 283 cell lines, or 45.4% of all cell lines, carried more than 2 copies of *GNAS*. The highest number of amplifications (5-9) was observed in pancreatic adenocarcinoma (HS766T), breast cancer (EFM19, AU565, HCC1954, HCC1428, MCF7, HCC1419, and UACC893), NSCLC (CHAGOK1), and colorectal cancer (SNU61 and HT55) cell lines.

### Analysis of association of expression of imprinted genes in tumor cell lines with drug response

**Fig. 2:**
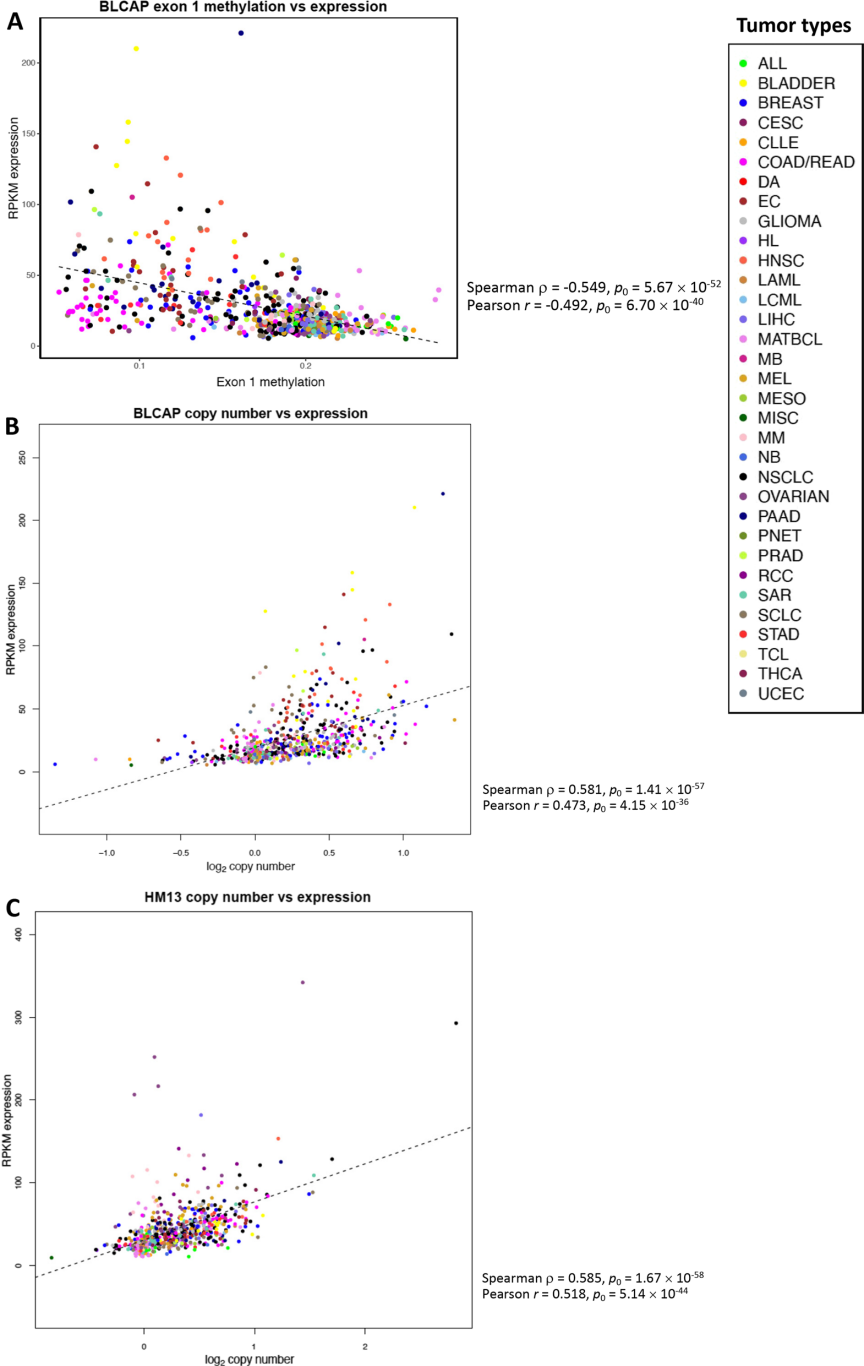
Scatterplots of the copy number, expression, and gene region methylation of the imprinted genes *HM13* and *BLCAP*. The dashed line indicates the linear regression line. The list of cancer categories is provided under Abbreviations. *ρ*, Spearman correlation coefficient; *r*, Pearson correlation coefficient. **A** Methylation of exon 1 of *BLCAP* versus *BLCAP* expression. **B** *BLCAP* copy number versus *BLCAP* expression. **C** *HM13* copy number versus *HM13* expression

Analysis of expression of imprinted genes in the pancancer data confirmed the association of increased expression of two genes, *BLCAP* and *HM13,* at 20q11-q13.32 with tumor cell resistance to multiple agents (Additional file 8: Table S5). Increased *BLCAP* expression was most strongly associated with resistance to the *ALK* inhibitor crizotinib (*ρ* = 0.3823, *p*_0_ = 2.03 × 10^–9^, *p*_FDR_ = 8.44 × 10^–8^), whereas elevated *HM13* expression had the strongest association with the antifolate agent methotrexate (*ρ* = 0.3600, *p*_0_ = 1.64 × 10^–17^, *p*_FDR_ = 7.45 × 10^–15^). Increased expression of both genes was also associated with resistance to multiple other agents. Using the criteria of |*ρ*| > 0.3, *p*_FDR_ < 0.05, we observed additional associations of *BLCAP* expression with resistance to BX795, cyclopamine, lestaurtinib, PD173074, and salubrinal and of *HM13* expression with resistance to AR-42, axitinib, BX-912, daporinad, GSK429286A, imatinib, ispinesib mesylate, linifanib, NPK76-II-72-1, panobinostat, PFI-3, QL-XI-92, quizartinib, ruxolitinib, SGC0946, T0901317, tivozanib, TL-2-105, topotecan, tubastatin A, UNC1215, vorinostat, VX-702, XMD13-2, XMD14-99, XMD15-27, and zibotentan (Additional file 8: Table S5). Notably, all these associations involved resistance to a variety of agents, and neither *BLCAP* nor *HM13* expression was associated with drug sensitivity. Expression of both genes was strongly and positively correlated with their copy number (*ρ* = 0.58, *p*_0_ ≤ 1.41 × 10^–57^; Fig. 2).

When considering all imprinted genes located in different chromosomal regions, not restricted to the 20q11-q13.32 chromosomal region, the strongest association between baseline expression and drug response was observed for the histone deacetylase (HDAC) inhibitor panabinostat with *CPA4* (carboxypeptidase A4) gene (Additional file 8: Table S5). Interestingly, overexpression of *CPA4* was associated with resistance to panabinostat (*ρ* = 0.424, *p*_0_ = 1.45 × 10^–17^, *p*_FDR_ = 6.70 × 10^–15^). This association is consistent with the suggested functional involvement of CPA4 in the histone hyperacetylation pathway and the upregulation of the *CPA4* gene by histone deacetylase inhibitors [113, 114]. *CPA4* is located in the imprinted domain on 7q32 and is associated with aggressiveness of the prostate cancer and the poor prognosis of gastric cancer patients [113–115]. Neither the copy number of *CPA4* nor the copy number of any other genes on 7q32 were associated with drug response in our data (Additional file 7: Table S4), suggesting that the association of pretreatment *CPA4* expression with panabinostat resistance may be due to transcriptional regulation and functional involvement of *CPA4* rather than because of changes in the number of copies of that gene.

**Fig. 3:**
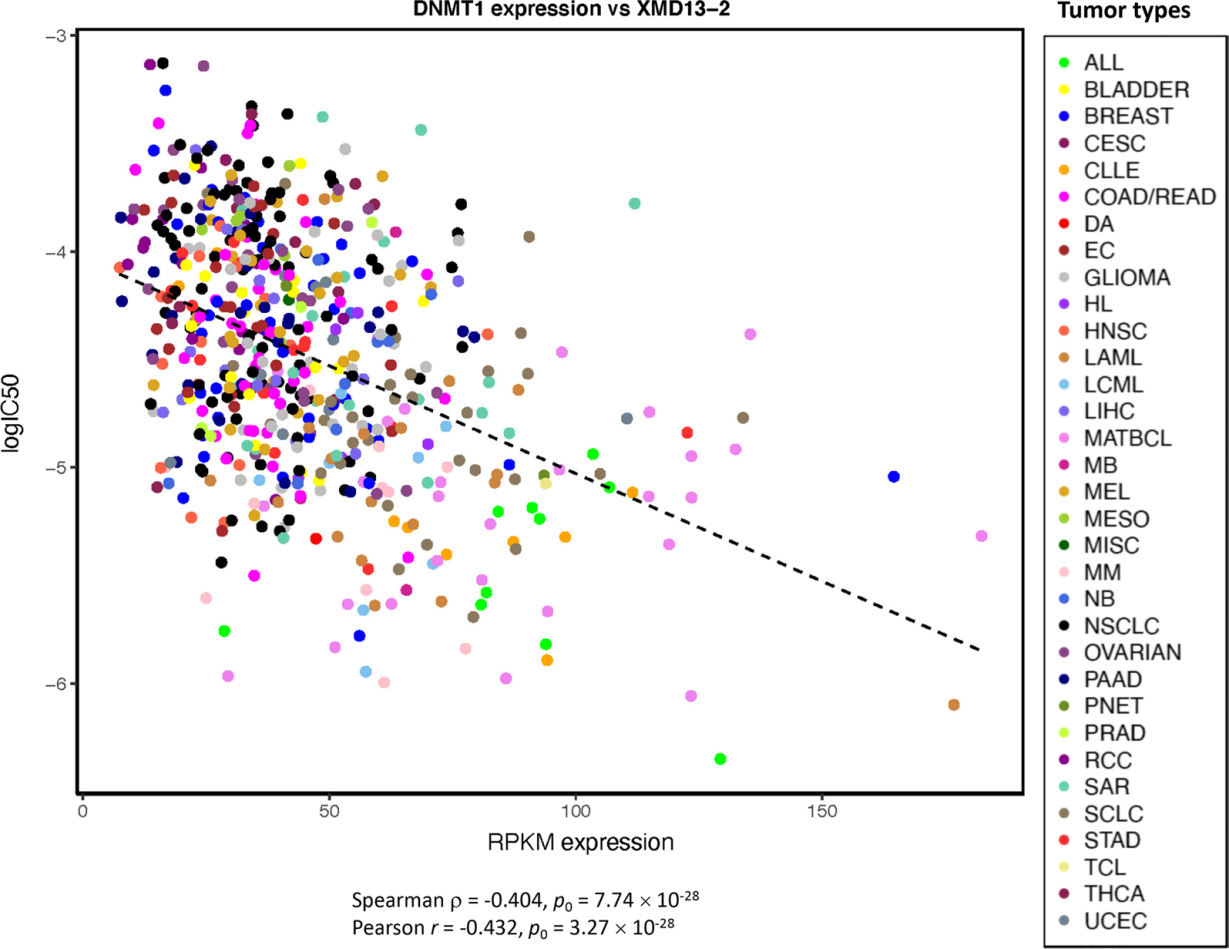
Scatterplot of log(IC50) of XMD13-2 vs expression of the *DNMT1* gene located in the chromosomal region 19p13.2. The dashed line indicates the linear regression line. The list of cancer categories is provided under Abbreviations. *ρ*, Spearman correlation coefficient; *r*, Pearson correlation coefficient

Many other top associations of gene expression included associations of expression of *DNMT1,* located at 19p13.2, with sensitivity to multiple agents (− 0.421 ≤ *ρ* ≤ − 0.301, *p*_FDR_ ≤ 2.36 × 10^–5^), and associations of expression of *PHLDA2* at 11p15.4 with response to multiple agents, predominantly with drug resistance (0.301 ≤|*ρ*| ≤ 0.402, *p*_FDR_ ≤ 5.18 × 10^–5^; Additional file 8: Table S5; Fig. 3). Both genes are imprinted in the placenta [53, 54, 116]. We previously noted associations of *DNMT1* expression with drug sensitivity in the CCLE-GDSC dataset [78], which may be explained by the profound effect of the *DNMT1* product, DNA methyltransferase 1, on epigenome-wide DNA methylation [117]. DNMT1 also plays a crucial role in regulating monoallelic expression of the imprinted genes [116, 118, 119]. *DNMT1* expression was associated with sensitivity to 68 agents with Spearman *ρ* < − 0.3, including the strongest associations with zibotentan, XMD13-2, and daporinad (*ρ* < −0.4; Fig. 3). *DNMT1* copy number showed a weak trend for association with sensitivity to PI-103 and THZ-2-102-1, whoever it did not reach the threshold of |*ρ*| > 0.3 (*ρ* = − 0.256 and − 0.268, respectively; Additional file 7: Table S4).

*PHLDA2* encodes pleckstrin homology-like domain, family A, member 2, which is involved in apoptosis and is a downstream target of EGFR and ErbB2 signaling [120]. It participates in fetal growth regulation, and its increased expression in placenta, where it is maternally expressed, has been associated with low birth weight [54, 116]. *PHLDA2* expression was associated with resistance to 54 agents with *ρ* > 0.3, including the strongest associated with the PDK1 inhibitor BX-912 with *ρ* = 0.402. Consistent with the association of its increased expression with resistance to many agents in our pancancer analysis, *PHDLA2* had been previously found to be a part of molecular signatures and pathways overexpressed in melanoma and multiple myeloma cell lines resistant to a BRAF inhibitor/MEK inhibitor combination and proteasome inhibitors, respectively [121, 122]. Interestingly, in our analysis *PHLDA2* expression was associated with sensitivity to four agents including three MEK inhibitors (trametinib, refametinib, and PD0325901) and an HSP90 inhibitor, tanespimycin (− 0.378 ≤ *ρ* ≤ − 0.329; Additional file 8: Table S5). None of the antitumor agents in the CCLE-GDSC dataset had their response associated with *PHLDA2* copy number, suggesting that the associations of drug response with expression of this gene in tumor cells may be influenced by its transcriptional regulation rather than by copy number changes.

Consistent with previous reports by our group and by other authors [78, 123–126], *RB1* expression was associated with sensitivity to the cyclin-dependent kinase (CDK) 4/6 inhibitor palbociclib (*ρ* = − 0.306, *p*_0_ = 1.79 × 10^–12^, *p*_FDR_ = 1.68 × 10^–10^) in accordance with the mechanism of action of palbociclib targeting the cyclin D–CDK 4/6–Rb pathway, in which RB1 is the major rate-limiting substrate [126].

### Analysis of association of methylation of imprinted genes in tumor cell lines with drug response

Higher methylation levels of gene regions of four imprinted genes at 20q11-q13.32 were significantly associated with drug sensitivity (*ρ* < − 0.3, *p*_FDR_ < 0.05; Additional file 9: Table S6). Increased methylation of the 1st exon of *BLCAP* and of the gene body of *NNAT* was associated with sensitivity to a variety of agents including cyclopamine, GSK269962A, GSK319347A, GSK429286A, XMD8-85, crizotinib, STF-62247, TL-1-85, JW-7-24-1, and salubrinal. *NNAT* is encoded in the first intron of *BLCAP* [127], and association of their methylation with an overlapping set of agents is consistent with their colocalization. Notably, these results are consistent with the association between increased expression of *BLCAP* and resistance to the inhibitor of the Sonic Hedgehog signaling pathway cyclopamine and the eIF2 signaling inhibitor salubrinal (Additional file 8: Table S5). Methylation of the 1st exon of *BLCAP* was significantly negatively associated with *BLCAP* expression (*ρ* = − 0.549, *p*_0_ = 5.67 × 10^–52^; Fig. 2).

Among other genes at 20q11-q13.32, methylation of the TSS200 of *SGK2* was associated with sensitivity to the inhibitor of JNK1 and p38 signaling ZG-10. Increased methylation of the gene body of the *GNAS* locus was associated with sensitivity to the dual LCK/SRC inhibitor WH-4-023.

When considering the regions of imprinted genes in all genome locations not restricted to 20q11-q13.32, 9 out of 11 of the strongest correlations (based on the absolute value of |*ρ*|) of methylation of gene regions of imprinted genes were with the ERK5/LLRK inhibitor XMD8-85 (Additional file 9: Table S6). The strongest association with drug response was observed for the methylation of exon 1 of *ANO1,* the gene encoding anoctamin 1 at 11q13.3. ANO1, a Ca^2+^-activated chloride channel protein, is involved in cancer cell proliferation, cell cycle changes, cell migration, and metastasis [104–106]. Methylation of the TSS200, the 5′ UTR, and the 1st exon of *DLX5,* which is located on 7q21.3 and encodes the distal-less homeobox 5 protein, was associated with sensitivity to XMD8-85 (− 0.3865 ≤ *ρ* ≤ − 0.3697, 2.74 × 10^–9^ ≤ *p*_0_ ≤ 1.46 × 10^–8^, 5.65 × 10^–7^ ≤ *p*_FDR_ ≤ 1.97 × 10^–6^; Additional file 9: Table S6). In contrast, *DLX5* expression was associated with resistance to that agent (*ρ* = 0.3540, *p*_0_ = 6.33 × 10^–8^, *p*_FDR_ = 1.59 × 10^–6^; Additional file 8: Table S5).

Methylation of the 5′ UTR of *NLRP2* and of TSS200 of *MIR371* at 19q13.42 was associated with sensitivity to the BMX inhibitor WZ-1-84 (*ρ* = − 0.384 and − 0.360, respectively, *p*_0_ ≤ 2.74 × 10^–8^, *p*_FDR_ ≤ 3.20 × 10^–6^; Additional file 9: Table S6). Methylation of the 5′ UTR of *NLRP2* was also associated with sensitivity to the ERK5/LLRK inhibitor XMD8-85 and to the multi-receptor tyrosine kinase inhibitor sunitinib (*ρ* = − 0.373 and − 0.336, respectively, *p*_0_ ≤ 2.46 × 10^–7^, *p*_FDR_ ≤ 1.69 × 10^–5^). *NLRP2* (NLR family pyrin domain containing 2) is a maternal effect gene which plays a role in early embryonic implantation and development and has an allelic expression bias in placenta [128, 129]. Genetic variants in this gene have been associated with multi-locus imprinting disturbance (MLID) [130, 131]. *NLRP2* is located near the imprinted *MIR371-MIR373* locus at 19q13.42 [132]. Neither the copy number of this chromosomal region, which also contains other imprinted genes [132], nor the expression of *NLRP2* or *MIR371* was associated with drug response (Additional file 7: Table S4; Additional file 8: Table S5), suggesting that methylation of the 5′ UTR of *NLRP2* could potentially influence the expression of some additional transcript or transcripts, which may affect tumor cell sensitivity to kinase inhibitors.

In the *DLK1-DIO3* imprinted gene cluster at 14q32.2-q32.31, methylation of exon 1 of *DLK1* was associated with sensitivity to WZ-1-84, whereas the TSS1500, TSS200, and the 5′ UTR upstream of *DIO3* were associated with sensitivity to XMD8-85, cyclopamine, CGP-082996, Z-LLNle-CHO, sunitinib, GNF-2, and crizotinib (Additional file 9: Table S6).

Sensitivity to XMD8-85 and to WZ-1-84 and resistance to the mTOR inhibitor temsirolimus included the agents that were the most strongly associated with DNA methylation of imprinted gene regions in different genome locations (*ANO1, DLX5, DIO3, NLRP2, MAGEL2, PLAGL1, DDC*, and *ZIM3*; Additional file 9: Table S6). Notably, none of these three agents were associated with the copy number of those respective genes, even though, as discussed above, *ANO1* had the highest range of copy number variation (0–17) among all genes analyzed in this study (Additional file 5: Table S3; Additional file 4: Fig. S2). Interestingly, copy number of several of these genes (*ANO1, DLX5, DIO3,* and *DDC*) and of other genes colocalized in the same chromosomal regions with them was weakly associated with sensitivity to several other kinase inhibitors, e.g., the BCR-ABL inhibitor imatinib, the ALK inhibitor crizotinib, and the cyclin-dependent kinase (CDK) inhibitor seliciclib (0.25 < *ρ* < 0.3; Additional file 7: Table S4).

Methylation of gene regions of *PHLDA2* was associated with sensitivity to multiple agents with diverse mechanisms of action, and with resistance to one agent, tanespimycin (|*ρ*| > 0.3; Additional file 9: Table S6)*.* A recent study reported that elevated methylation of cg1605792, one of the 24 CpG probes in the TSS1500 region of *PHDLA2,* in malignant breast tumors and in peripheral blood leukocytes was associated with increased breast cancer risk [21]. In our analysis, higher methylation of the TSS1500 of *PHDLA* was associated with sensitivity to BX-912, TL-1-85, ZG-10, and GNF-2 (Additional file 9: Table S6). As discussed above, *PHLDA2* expression was associated with drug response, predominantly resistance, to multiple agents (Additional file 8: Table S5), whereas no association with drug response was observed for the copy number of the chromosomal region 11p15.4 where *PHLDA2* is located. Consistent with the inverse directions of associations of *PHLDA2* gene region methylation and expression with drug response, methylation of *PHLDA2* gene regions was negatively and significantly associated with *PHLDA2* expression (*ρ* = − 0.507, − 0.345, − 0.372, − 0.339, and − 0.630 for TSS1500, TSS200, 5′ UTR, exon 1, and 3′ UTR, respectively, *p*_0_ ≤ 9.15 × 10^–19^ for all associations), in agreement with an earlier study [21]. While, similar to *PHLDA2, DNMT1* expression was associated with drug response (Additional file 8: Table S5) and both genes are imprinted in the placenta, none of the *DNMT1* gene regions had their average methylation associated with drug response (Additional file 9: Table S6).

### Copy number values of imprinted and non-imprinted genes in the chromosomal region 20q11-q13.32 were highly correlated with each other

**Fig. 4:**
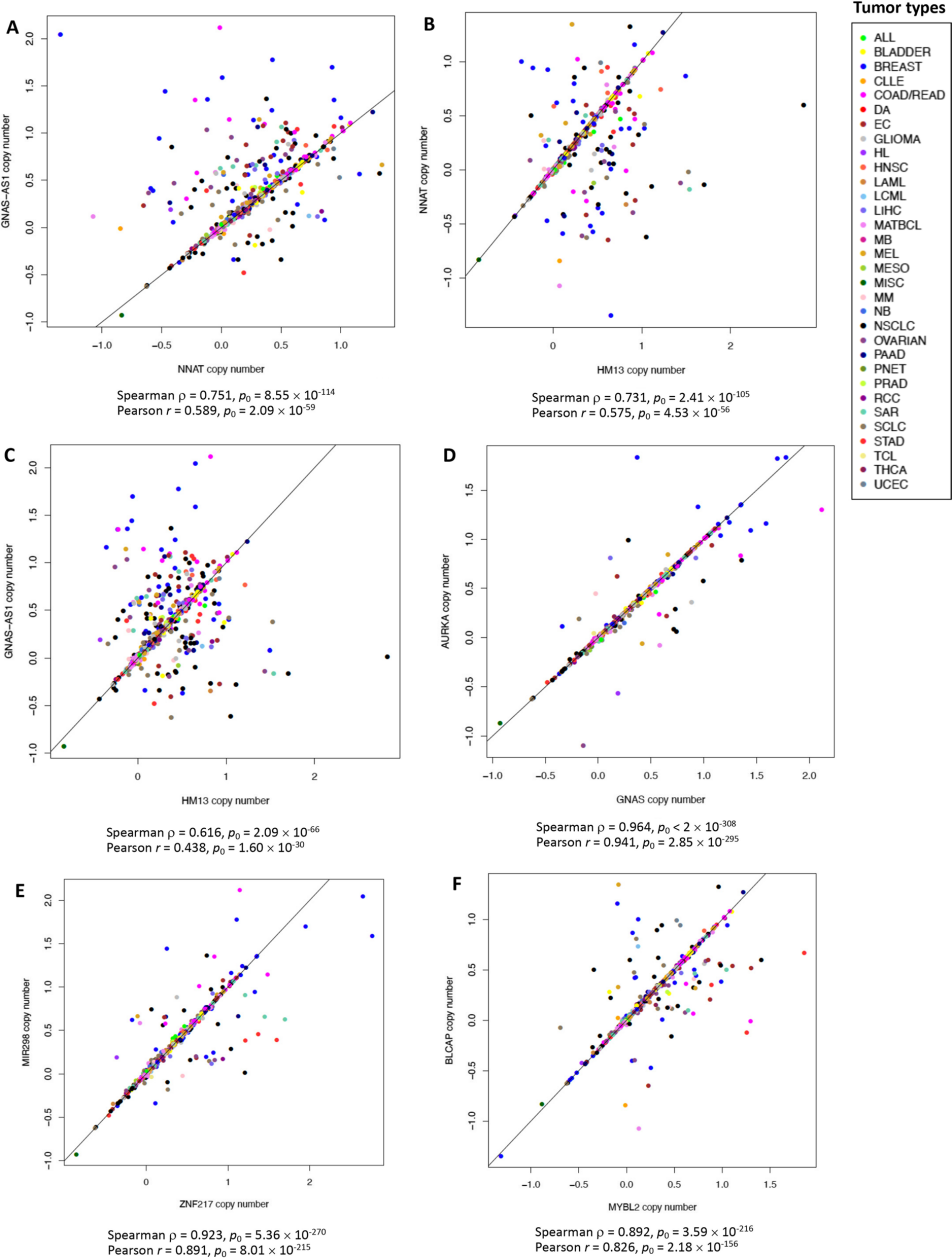
Example scatterplots showing strong correlations among copy number values of selected imprinted and non-imprinted genes at 20q11-q13.32. The solid line represents the identity line. The list of cancer categories is provided under Abbreviations. *ρ*, Spearman correlation coefficient; *r*, Pearson correlation coefficient. **A** Copy number of *NNAT* (located at 20q11.23) versus that of *GNAS-AS1* (at 20q13.32). **B** *HM13* (20q11.21) versus *NNAT* (20q11.23). **C** *GNAS-AS1* (20q13.32) versus *HM13* (20q11.21). **D** *GNAS* (20q13.32) versus *AURKA* (20q13.2). **E** *ZNF217* (20q13.2) versus *MIR298* (20q13.32). **F** *MYBL2* (20q13.12) versus *BLCAP* (20q11.23)

The chromosomal region 20q11-q13.32 contains several subamplicons which are co-amplified in a variety of tumors including breast, ovarian, and prostate cancer [95, 133]. In contrast, the long arm of 20q and specifically the chromosomal region 20q12 are commonly deleted in myelodysplastic syndrome (MDS), AML, and chronic myeloid malignancies [72, 134]. Loss of 20q13.12 is also often found in follicular thyroid carcinomas and atypical adenomas, where copy number loss of chromosomal regions enriched in imprinted genes has been observed [39]. In addition to imprinted genes, the 20q11-q13.32 chromosomal region contains multiple non-imprinted genes involved in cancer, e.g., *AIB3*, *AIB4*, *AURKA* (*STK6*), *BTAK, MYBL2, PTPN1, STK15,* and *ZNF217* [77, 95, 133, 135]. We examined associations among copy number values of imprinted genes at 20q11-q13.32, listed in Table 1 due to their association with drug response, with copy number values of the non-imprinted cancer genes *MYBL2* located at 20q13.12, and *AURKA* and *ZNF217* at 20q13.2. These three non-imprinted genes are commonly amplified in cancer [133, 136–138]. Copy number values of imprinted genes at 20q11-q13.32 were strongly and significantly (Spearman *ρ* between 0.63 and 0.97, Pearson *r* between 0.46 and 0.96, *p*_0_ ≤ 4.85 × 10^–34^) correlated with each other and with copy number of non-imprinted genes *MYBL2, AURKA,* and *ZNF217* in the same chromosomal region (Additional file 10: Table S7; Fig. 4). Consistent with correlations among the gene copy number values, higher copy number of the non-imprinted genes *AURKA* and *ZNF217* was also associated with resistance to ispinesib mesylate, T0901317, cyclopamine, BX-912, GSK429286A, XMD14-99, GSK1070916, UNC1215, TL-2-105, XMD13-2, and QL-XI-92 (0.301 ≤ *ρ* ≤ 0.349) The associations for the copy number of *MYBL2* were weaker and did not reach the threshold of |*ρ*| >0.3 (data not shown).

While the presence of multiple imprinted and non-imprinted genes at 20q11-q13.32 may be confounding the associations of the copy number of individual genes with drug resistance, only the expression of the imprinted genes *BLCAP* and *HM13*, located in that region, was associated with resistance to the genes listed in Table 1 (Additional file 8: Table S5; Fig. 3). Among the non-imprinted genes, expression of *MYBL2* was correlated with sensitivity to GNF-2 and CGP-60474 (*ρ* = − 0.311 and − 0.300, respectively, *p*_0_ ≤ 4.59 × 10^–6^; data not shown). All other associations of expression of the non-imprinted genes *MYBL2, AURKA,* and *ZNF217* with drug response were weak (|*ρ*| <0.3; data not shown). This suggests that expression of these three non-imprinted cancer genes may not be the primary driver of the association between the copy number of the multiple imprinted genes at 20q11-q13.32 and cancer drug resistance.

### Association analysis of copy number of imprinted genes with drug response in individual tumor categories of tumor cell lines

Association analysis of imprinted genes assigned to 35 chromosomal segments with 275 drug response measures in 22 individual tumor categories resulted in 169,365 chromosomal segment–agent–tumor category combinations with available data. After FDR adjustment for multiple testing, none of the chromosomal segments were associated with drug response in individual tumor categories. The lowest *p*_SegmFDR_ = 0.2043 was observed for three chromosomal regions, including the association of *RBP5* copy number at 12p13.31 with sensitivity to KIN001-236 (*ρ *= − 0.773, *p*_0_ = 2.30 × 10^–6^) in ovarian cell lines, of the *DLK1*-*DIO3* imprinted cluster at 14q32.2-q32.31 (including copy number of *DIO3*, *MIR134, MIR379*, *MIR409*, *MIR410*, *MIR487B*, *MIR656, DLK1*, *MEG3*, *MEG8*, and *RTL1*) with sensitivity to bleomycin in renal cell carcinoma cell lines ( ≤ *ρ* ≤ − 0.318, respectively, 3.00 × 10^–6^ ≤ *p*_0_ ≤ 9.91 × 10^–6^), and the association of the 15q25.1 chromosomal region containing *RASGFR1* and *MIR184* with resistance to both idelalisib in colorectal adenocarcinoma cell lines (COAD/READ; *ρ* = 0.667, *p*_0_ = 3.60 × 10^–6^) and to KIN001-244 in liver hepatocellular carcinoma (LIHC; *ρ* = 0.891, *p*_0_ = 3.62 × 10^–6^; data not shown).

### Expression profiles of imprinted and non-imprinted genes at 20q11-q13.32 were associated with ex vivo drug response in the Beat AML cohort

In the CCLE-GDSC pancancer dataset, molecular measures of genes at 20q11-q13.32 were correlated with response to multiple agents, many of which are used in the treatment of hematological malignancies [99, 100]. In order to validate these findings, we used available expression measures of genes in that chromosomal region and ex vivo drug response data from tumor samples from an independent cohort of acute myeloid leukemia patients, Beat AML 1.0 [46]. We examined associations of expression of genes at 20q11-q13.32 with all agents from Additional file 7: Table S4 that were associated in the CCLE-GDSC dataset with copy number of the genes in that region. Increased expression of several genes at 20q11-q13.32 was significantly associated with drug resistance in AML tumor samples (Additional file 11: Table S8; Additional file 12: Fig. S4). They included the imprinted genes *SGK2, L3MBTL1, NNAT,* and *GNAS* and the non-imprinted gene *ZNF217.* Similar to the CCLE-GDSC cell line dataset, the majority (8 out 10) of associations in AML cells satisfying Spearman |*ρ*|> 0.25 and *p*_FDR_ < 0.1 were positive, indicating that increased gene expression in patient leukemia cells was associated with their resistance to nilotinib, TG101348 (fedratinib), lestaurtinib (CEP-701), and panobinostat. Only the directions of associations with lenalidomide were mixed, including a positive correlation with *ZNF217* expression and negative correlations with expression of *GNAS* and *SGK2.* The three strongest associations with drug resistance in the Beat AML dataset involved correlations of *SGK2* and *L3MBTL1* expression with resistance to nilotinib and of *NNAT* expression with resistance to TG101348 (0.3005 ≤ *ρ* ≤ 0.3343, 2.40 × 10^–9^ ≤ *p*_0_ ≤ 0.0058, 2.80 × 10^–7^ ≤ *p*_FDR_ ≤ 0.0451; Additional file 11:Table S8). Associations of expression of *GNAS* with resistance to the kinase inhibitors TG101348, nilotinib, and lestaurtinib and of *NNAT* expression with TG101348 are consistent with significant associations of their copy number with response to the same agents in the pancancer analysis of CCLE-GDSC cell lines (Additional file 7: Table S4). Expression of *HM13* and *BLCAP,* which was significantly associated with drug resistance in the pancancer CCLE-GDSC dataset, did not pass the threshold for association in the AML dataset. Results of Pearson correlation analysis were similar to Spearman correlation results (Additional file 12: Fig. S4).

While expression of multiple genes at 20q11-q13.32 was associated with drug response in the Beat AML 1.0 cohort, it was not significantly associated with overall patient survival for any of these genes after adjustment for multiple testing beyond that expected by chance (*p*_FDR_ ≥ 0.2198; data not shown). Similarly, a log-rank test showed no significant differences in overall survival between patients with and without deletions of 20q11-q13.32 based on cytogenetic information (*p* = 0.44; hazard ratio = 1.23; 95% CI 0.73–2.07).

## Discussion

We observed a modest significant association of the copy number of imprinted genes in the chromosomal region 20q11-q13.32 with response to multiple antitumor agents, including a number of kinase inhibitors. This chromosomal region contains multiple imprinted and non-imprinted genes involved in cancer, cell growth, and cell proliferation. *BLCAP,* encoding the bladder cancer-associated protein and located at 20q11.23, is imprinted in the human brain [51, 139, 140]. While *BLCAP* has been reported to be a tumor suppressor gene promoting apoptosis [127, 140], its product interacts with STAT3 and has been suggested to promote bladder cancer progression [141]. The neuronatin gene *NNAT,* encoded in the first exon of *BLCAP,* is imprinted in multiple tissues, with paternal allelic expression [127]. Its expression is elevated in several tumor categories, and increased expression is associated with tumor aggressiveness and worse outcomes in several cancer categories [127]. Due to their co-location, copy number values of *BLCAP* and *NNAT* were fully correlated (Pearson and Spearman correlation coefficients = 1, *p*_0_ = 0; Additional file 10: Table S7). Methylation of both the 1st exon of *BLCAP* and the body of *NNAT* was associated with sensitivity to several agents (Additional file 9: Table S6), whereas elevated *BLCAP* expression was associated with resistance to two of these agents, cyclopamine and subluminal, and increased copy number of both *BLCAP* and *NNAT* was associated with drug resistance (Additional file 7: Table S4 and Additional file 8: Table S5).

Our findings indicate that expression of *BLCAP* is positively associated with its copy number and negatively with methylation of its 1st exon (Fig. 2), and therefore either its expression, DNA methylation, or copy number, or a combination of these factors may contribute to drug response. The direction of correlations in our analysis of cell line data was consistent with earlier reports of associations of increased BLCAP protein expression and lower *BLCAP* promoter methylation with inferior survival of bladder cancer patients [142, 143]. Earlier studies showed that *BLCAP* expression involves different transcripts which are expressed in a promoter-specific and tissue-specific manner and that the *BLCAP* transcript expressed in human fetal brain is imprinted, with predominantly maternal expression [140]. Further studies may be needed to clarify whether a specific *BLCAP* transcript, expression of which may be regulated by methylation of the first exon, may play a role in drug response. In addition to expression, cellular localization patterns of BLCAP have been reported to be associated with survival of bladder cancer patients and breast cancer patients with lobular carcinomas [142, 144]. Therefore, potential effects of nuclear or cytoplasmic localization of BLCAP on tumor response to treatment warrant further investigation. In our study, both increased expression and higher copy number of *BLCAP* were associated with drug resistance (with positive *ρ*). This direction of association could be due to potential therapeutic vulnerability of tumor cells with fewer copies of the *BLCAP* gene and lower *BLCAP* expression. An alternative explanation could be a possibility of an indirect association of *BLCAP* expression with drug response due to an increased number of copies of multiple other genes located in that chromosomal region.

Increased copy number and elevated expression of the *HM13* gene, encoding minor histocompatibility antigen H13 at 20q11.21, were also associated with resistance to multiple agents in the CCLE-GDSC dataset (Additional file 7: Table S4; Additional file 8: Table S5; Figs. 1 and 2). Interestingly, Miranda et al. [145] found methylation of this gene to be a part of a gene signature predictive of gemcitabine response in the GDSC dataset, whereas in our gene region-focused analysis we did not find any associations of methylation of any regions of *HM13* with drug response (Additional file 9: Table S6). The genome location of *HM13* overlaps with that of the imprinted pseudogene *PCIMST-1* [26, 146]. Interestingly, in breast cancer, most prominently in the luminal B subtype, overexpression of *HM13* was found to be caused by its biallelic expression due to the loss of imprinting, and it was independent of the copy number gain [26]. In contrast, we found a strong and highly significant correlation between copy number and expression levels of *HM13* in our analysis (Spearman *ρ* = 0.585, *p*_0_ = 1.67 × 10^–58^, Pearson* r* = 0.518, *p*_0_ = 5.14 × 10^–44^; Fig. 2C). A population-based survey of 23,116 non-cancerous human epigenomes from blood, umbilical cord blood, purified monocytes, and adipose tissue found biallelic methylation or biallelic hypomethylation of *HM13* to be the most common epigenome variation, with approximate rates of 1 per 350 and 1 per 3300 individuals, respectively [147]. These findings suggest the complexity of epigenetic regulation of *HM13* and a potential difficulty in the interpretation of downstream phenotypic effects of *HM13* overexpression in tumor cells.

Among the imprinted genes at 20q13.32 whose copy number was associated with drug resistance, the *GNAS* (guanine nucleotide-binding protein, alpha-stimulating) gene encodes a growth-promoting factor. Genetic variation in *GNAS* is associated with birth weight [11, 148]. Mutations in the *GNAS* gene are frequently observed in a variety of malignant, premalignant, and benign tumors and in tumor-derived organoids and patient-derived xenografts [19, 149–154]. This gene has been reported to play an oncogenic role in SCLC. It is activated in a subset of human SCLC tumors through either gene amplification or mutational mechanisms, and *GNAS* activation significantly increases growth and progression of mouse SCLC models [155]. Consistent with common activation of *GNAS* in tumors, we observed a frequent amplification of this gene (in 45.4% of all tumor cell lines), whereas it had a deletion of one copy in only 0.8% of the cell lines. We also found that higher methylation of the gene body of the *GNAS* locus was associated with sensitivity to the dual LCK/SRC inhibitor WH-4-023. The complex *GNAS* locus encodes several transcripts which are imprinted (*NESP55*, *XLαs, A/B*, *GNAS-AS1*, *miR296*, and *miR298*) or have tissue-dependent imprinting patterns (*Gsα*) [19, 64, 156]. The *Gsα* transcript in maternally expressed in the pituitary, proximal renal tubule, gonads, and thyroid tissues, and neonatal brown adipose tissue, and biallelically expressed in other normal somatic tissues, whereas the *NESP55* of *GNAS* is exclusively maternally expressed, and *XLαs, A/B*, and *GNAS-AS1* are paternally expressed across tissues [2, 19, 64, 156]. Regulation of these transcripts through DNA methylation is complex and is affected by alternative promoters, with methylated regions located predominantly outside of the *Gsα* exons [156]. It remains to be investigated which transcripts in the *GNAS* locus may have their expression affected by the gene body methylation of *GNAS,* which was associated with WH-4-023 sensitivity in our study.

Interestingly, higher copy number of the imprinted genes *L3MBTL1* and *SGK2* at 20q13.12 was weakly associated with drug resistance (Additional file 7: Table S4), and their increased expression was associated with resistance to several agents in leukemia tumor samples from the Beat AML study (Additional file 11: Table S8, Additional file 12: Fig. S4). The actively expressed, paternally inherited copies of both genes are commonly deleted in myeloproliferative neoplasms, which has been suggested to contribute to epigenetic dysregulation of chromatin function; in some cases, both genes are lost as part of larger areas, or the entire long arm of 20q is deleted, which is a frequent event in hematologic neoplasms [72, 134, 157–161]. In the CCLE-GDSC dataset, each of these two genes had 1–7 copies across all cancer categories. Only a small proportion of the cell lines (14 for *L3MBTL1*, or 2.2% and 17 for SGK2, or 2.7% of the total) had a loss of one copy of either gene. Among them, three lymphoma cell lines, Hodgkin lymphoma lines HDLM2 and KMH2 and diffuse large B cell lymphoma (DLBCL) A3KAW, had a loss of a copy of one or both genes. Copy number of both *L3MBTL1* and *SGK2* was associated with sensitivity to ispinesib mesylate in pancancer analysis of CCLE-GDSC tumor cell lines. Ispinesib is considered a drug candidate for lymphoma treatment [162]. We also observed the loss of one or both of these genes in several lung, breast, and ovarian cell lines (data not shown). Many other cell lines from the same and other tumor categories including solid tumors and hematopoietic and lymphoid malignancies had an amplification (3 or more copies) of one or both genes (244 and 243 cell lines, for *SGK2* and *L3MBTL1,* respectively, representing 39% of all cell lines). Because individual tumor types such as lymphomas and lung, breast, and ovarian tumor types each had a range of copy number loss and gain of these two genes, the weakly positive association between copy number of the imprinted genes at 20q13.12 is unlikely to be caused by the tissue specificity of ispinesib mesylate activity. The data from the Beat AML leukemia dataset show that a number of tumor samples with cytogenetic deletions of all or a part of 20q had high levels of expression of *SGK2* or *L3MBTL1* (Additional file 12: Fig. S4), suggesting possible compensatory mechanisms of upregulation of expression of the remaining allele of both genes or potential tumor heterogeneity of some samples.

Our findings from cell line data analysis are consistent with an earlier report of Martin-Trujillo et al. [14] who observed frequent amplifications and rare deletions of *GNAS, BLCAP, L3MBTL1,* and *MSCT2* (*PSIMCT*-*1*) in lung, colorectal, breast, and hepatocellular carcinoma primary tumors from the Cancer Genome Atlas (TCGA), including a range of copy number gains and losses of all four genes in lung cancer tumors. Martin-Trujillo et al. [14] noted a potential role of genome co-location of non-imprinted oncogenes and tumor suppressor genes with imprinted genes in other genome regions in their effect on cancer processes. It is unclear whether the association of the amplification of the 20q11-q13.32 region with drug resistance identified in our analysis is driven by multiple imprinted genes at 20q11-q13.32, either individually or in a cooperative manner, or whether non-imprinted oncogenes and tumor suppressor genes, and other non-imprinted genes located in the same chromosomal region may be contributing to drug response. For example, the imprinted gene *GNAS* and the non-imprinted gene *AURKA,* both important in cancer pathogenesis, had nearly identical copy number in our data (*ρ* = 0.964, *p*_0_ < 2 × 10^–308^; Fig. 4; Additional file 10: Table S7). The three non-imprinted genes at 20q11-q13.32 examined in our study, *AURKA, MYBL2,* and *ZNF217*, have a strong influence on cell cycle, proliferation, signaling, survival, and differentiation of malignant cells and on cancer progression, and they have been implicated in cancer drug resistance [136, 137, 163–166]. Expression of *AURKA, MYBL2,* or *ZNF217* was not associated with drug resistance in our pancancer analysis of cell lines, which may suggest that the association between the copy number of imprinted genes at the 20q11-q13.32 and cancer drug resistance may not be due to the direct effects of these non-imprinted genes. In support of this suggestion, the association of *MYBL2* and *ZNF217* expression with their copy number in our analysis of pancancer cell line data was very weak (|*ρ*| < 0.24, data not shown). However, potential influences of these non-imprinted genes on drug response cannot be excluded, as expression of *ZNF217* was weakly associated with response to lenalidomide in the Beat AML dataset (*ρ* = 0.261, *p*_FDR_ = 0.065; Additional file 11: Table S8, Additional file 12: Fig. S4). While overexpression of both genes was previously reported to be associated with 20q13 amplification in tumors [95, 138], earlier studies demonstrated that additional mechanisms may also induce overexpression of both *MYBL2* and *ZNF217* [133, 135], which may explain the lack of association between copy number and expression of *MYBL2* and *ZNF217* in our analysis. Of note, *AURKA* expression in our pancancer analysis of cell lines was strongly positively correlated with *AURKA* copy number (*ρ* = 0.541, *p*_0_ = 1.42 × 10^–48^), even though the correlation of *AURKA* expression with drug response in the CCLE-GDSC dataset was very weak (|*ρ*| < 0.22; data not shown). A number of additional non-imprinted genes at 20q11-q13.32 are also overexpressed in tumors with amplifications of that chromosomal region [133–135], and the overall impact of the amplification or copy number loss of this region on drug response may be complex and could involve both imprinted and non-imprinted genetic components. A more detailed future investigation is needed to examine the potential effects of interactions between imprinted and non-imprinted genes in that chromosomal region in drug resistance.

In addition to possible interactions among imprinted and non-imprinted genes located in close proximity from each other, earlier studies identified extensive networks of interactions and co-regulation in mammalian growth and differentiation between imprinted and non-imprinted genes located in different genome regions, [3, 167]. For example, the antiapoptotic factor BIRC5*,* whose gene is located at 17q25.3, has regulatory interactions with several imprinted genes, with the strongest connection to *PLAGL1* (*ZAC1*) at 6q24.2 [2, 167]. ZAC1 directly regulates expression of multiple imprinted and non-imprinted genes, acts as co-activator of nuclear hormonal receptors, and may enhance transcriptional activity of p53 [2]. Such interactions have been hypothesized to directly influence tumor response to therapy, including potential tumor-specific effects [2]. Our study did not consider interactions between imprinted and non-imprinted genes, which represents a potential limitation of its findings. A possible influence of the copy number of imprinted genes on expression of non-imprinted genes and on functional regulation of their protein products is an intriguing direction which needs to be explored in future studies.

Among imprinted genes in all chromosomal regions analyzed in this study in tumor cell lines, only imprinted genes in the chromosomal region 20q11-q13.32 showed a consistent modest association of their copy number, expression, and DNA methylation with drug response. Significant associations of the copy number data in that region were observed only in the pancancer dataset. The lack of statistical significance for associations between copy number and drug response in individual cancer categories may be due to small sample sizes (10–93 cell lines) in individual tumor types and a very large number (169,365) of individual comparisons for multiple drugs, cancer categories, and chromosomal segments. Such a large number of tests likely resulted in some true positive associations in individual tumor types not reaching statistical significance. Further studies with sufficiently large sample sizes in individual cancer categories may provide an additional insight into potential effects of the copy number gain or loss of the chromosomal regions containing the imprinted genes on drug response within specific cancer categories. Adequately powered analysis within individual cancer categories may be particularly relevant for associations of gene expression and DNA methylation data. We observed cancer-specific differences in expression and drug response, although the patterns of copy number change among the genes at 20q11-q13.32 were more consistent among many tumor types (Additional file 13: Fig. S5, Additional file 14: Fig. S6, Additional file 15: Fig. S7). Previous studies also reported tissue-specific variation in expression of imprinted genes in other genome locations, including those genes whose expression was associated with drug response in our analysis (Additional file 8: Table S5), e.g., *DLK1* at 14q32.2-q32.31 [22]. As an example of previously reported tissue-specific associations, an earlier study found an association of lower DNMT1 protein expression with improved histopathological and clinical response in gastric cancer patients treated with a combination of platinum therapy and 5-florouracil, and with in vitro sensitivity to cisplatin in gastric cancer cell lines [168]. While none of the agents analyzed in that study were associated with *DNMT1* expression in our pancancer analysis (Additional file 8: Table S5), we observed an association of increased *DNMT1* expression with sensitivity, rather than resistance, to multiple other agents in the pancancer dataset.

Our analysis of tumor samples from the Beat AML study found that expression of the imprinted genes *SGK2, L3MBTL1, NNAT,* and *GNAS* and of the non-imprinted gene *ZNF217* at 20q11-q13.32 was weakly or modestly associated with response, predominantly resistance, to multiple antitumor agents that were also associated with copy number of genes at 20q11-q13.32 in our pancancer cell line analysis. Significant correlations of increased *GNAS* expression with resistance to TG101348, nilotinib, and lestaurtinib and of *NNAT* expression with resistance to TG101348 were directly parallel to the associations of the increased copy number of both imprinted genes with response to the same agents in the CCLE-GDSC dataset. While regulation of gene expression in different tumor categories may involve different mechanisms in addition to copy number changes, this independent validation provides strong support for our initial findings of the potential role of that chromosomal region in drug resistance.

Molecular influences of the 20q11-q13.32 chromosomal region in the response of leukemia cells to drug treatment may be complex. In addition to the frequent loss of *SGK2, L3MBTL1,* and other genes as part of the 20q deletion, this region also includes the non-imprinted epigenetic regulator *ASXL1* gene at 20q11.21. *ASXL1* is frequently mutated in hematological malignancies, and its mutations have been associated with drug resistance, inferior response to treatment, and poor prognosis of patients with leukemia or MDS [158–160, 169–171]. The presence of *ASXL1* mutations was associated with increased expression of *PEAR1,* a biomarker of inferior patient survival in the expanded Beat AML 2.0 cohort [101]. Consistent with this effect of *ASXL1* mutations, we found that they commonly occurred in drug-resistant AML cells, both in samples with high and low levels of expression of other genes at 20q11-q13.32 that were associated with response to the same agents in our analysis (Additional file 12: Fig. S4).

While our analysis of AML data supported the potential effect of multiple genes at 20q11-q13.32 on drug response, their expression or the loss of that cytogenetic region were not significantly associated with overall patient survival in our analysis. The lack of association of genes in that region with survival of AML patients may be explained by multiple factors, e.g., weak to modest associations of individual genes which were associated with drug response, complex drug treatment regimens of the patients involved in the study, and genetic and clinical heterogeneity of the study patients [46]. Some effects of the genes at 20q11-q13.32 analyzed in this study may be tumor-specific, as suggested by the findings of Moreira et al. [142] and Chen et al. [143] of associations of BLCAP protein expression and promoter methylation levels with survival of bladder cancer patients, which were consistent with our analysis of drug response in pancancer cell lines. Similarly, Anwar et al. [37] reported an association of survival of hepatocellular carcinoma patients with increased methylation of several of the same imprinted genes and imprinted gene regions (*GNAS* at 20q13.32*,* the *DLK1-DIO3* cluster at 14q32.2-q32.31*,* and *ZIM3* at 19q13.43) in primary tumor samples, which were also associated with increased drug sensitivity in our pancancer analysis of tumor cell lines (Additional file 9: Table S6).

Precise identification of the parental origin of each allele of the imprinted genes in our study was not possible since no maternal or paternal genetic information, or matching normal tissue data were available for the cell lines used in our study. In the absence of matched normal samples or parental samples, we investigated the overall effects of the variation in the combined measures for both alleles for copy number, expression levels, and DNA methylation of imprinted genes on drug response. Our study did not examine potential effects of allele-specific expression or methylation of imprinting control regions of the imprinted genes on drug sensitivity or resistance. In support of our approach, a study of 280 GDSC lung, colorectal, breast, and hepatocellular carcinoma primary tumors [14] found that copy number changes of imprinted genes have a more common occurrence and a stronger influence on methylation of imprinted loci in tumor cells than does the change in their imprinted status. However, regulation of imprinted genes and changes in the imprinting status in cancer may be complex. Some tumor cells exhibit loss or gain of imprinting or the switch of allele-specific expression, either with or without the switch of imprinting between parental alleles [13, 28]. A recent study identified aberrant allelic expression of both *GNAS* and *HM13* at 20q11-13.32, as well as that of imprinted *GRB10* at 7p12.1 and *SNRPN* 15q11.2 as useful biomarkers for lung cancer diagnosis [29]. Future detailed analysis of how of allele-specific changes in imprinting status of imprinted genes at 20q11-q13.32 and in other genome locations may influence drug response would provide a more refined understanding of how imprinted genes may contribute to response to individual agents. In such analysis, the parental origin of both alleles could be inferred using tumor datasets with available family data or matching normal tissues from the same individuals. In addition, since our study found associations of drug response with copy number changes of the 20q11-q13.32 region that included multiple genes with diverse parent-of-origin imprinting patterns in the normal tissues, in the future would be beneficial to investigate parent of origin-specific copy number changes of each allele of the imprinted genes in a dataset where such information could be inferred. Such analysis would be able to examine whether alleles inherited from a particular parent may be preferentially lost or gained for specific imprinted genes and in particular tumor types.

Without the information about the parent of origin of each allele, available RNA-seq data for the cell lines provide an opportunity to conduct a follow-up investigation of whether transcript isoforms of imprinted genes are mono- or biallelically expressed, or whether they may have a partial biallelic expression. Experimentally identified aberrant biallelic and multiallelic expression and increased total expression of imprinted genes have been shown to be useful as cancer biomarkers [24, 29]. We are currently pursuing a follow-up large-scale bioinformatic project to infer the haplotype status of both alleles and the extent of expression of both alleles of transcriptional isoforms of the imprinted genes using RNA-seq expression data. Information derived from this follow-up analysis may help refine the potential effect of the allelic dosage in expression of imprinted genes on drug response.

A more detailed follow-up analysis may also be important since our study included a variety of genes, some of which are imprinted at specific developmental stages or in specific tissues, with varied imprinted patterns among several overlapping mRNA transcripts from the same gene locus. For example, *PHLDA2,* which is located at 11p15.4 and the gene expression and methylation of which were associated with response to multiple agents in our study (Additional file 8: Table S5, Additional file 9: Table S6), is imprinted in the placenta, but it is not imprinted in lymphoblastoid cell lines or skin fibroblasts [71]. *DNMT1,* whose expression was associated with drug response (Additional file 8: Table S5)*,* is located at 19p13.2 and also has placenta-specific imprinting [53, 128]. As discussed above, *GNAS* and *BLCAP* at 20q11-q13.32 have complex transcript-dependent and tissue-dependent imprinting patterns [2, 19, 64, 140, 156]. A number of additional genes in other genome regions are also imprinted in a tissue-specific manner in adult or fetal tissues. They include, e.g., *CPA4, PLAGL1, IGF2, GRB10,* and other genes whose copy number, expression, and/or methylation measures were associated with drug response (Additional file 7: Table S4, Additional file 8: Table S5, Additional file 9: Table S6) [2, 71, 113, 140, 172]. Tissue specificity of their imprinting, in addition to variation in gene expression among tissues regulated by mechanisms other than imprinting, underscores the importance of future analyses of associations of allelic dosage, parent-specific allelic expression, and novel types of omics data [3] with drug response, which would need to be conducted in separate tumor categories with large sample sizes. An additional analysis of the mutation status of the imprinted genes in tumors would add further depth to the understanding of their influence on drug response, since protein-changing mutations in many imprinted genes including those genes which were associated with drug response in our study, e.g., *NLRP2, CDKN1C,* and *GNAS,* commonly occur in patients with imprinted disorders and/or cancer [1, 129, 131, 149–154, 156].

Our analysis was based on the gene-level copy number data generated by the CCLE Consortium using Affymetrix Genome-Wide Human SNP Array 6.0 arrays [40, 41]. Due to the density of the array probes, copy number information in this dataset was available at the whole gene level and also for focal copy number changes corresponding to relatively large genome regions. While we analyzed amplifications and deletions at the whole gene level, these changes often involved large genome regions containing multiple genes. The 20q11-q13.32 chromosomal region, associated with drug response in our study, contained multiple imprinted and non-imprinted genes whose copy number was strongly correlated with each other, consistent with prior evidence that multiple genes in that region are often co-amplified or co-deleted [39, 72, 95, 133, 134, 173]. While evidence for events on a smaller scale involving imprinted genes in cancer is limited, small germline microdeletions, microinsertions, and chromosomal rearrangements have been reported within the *GNAS* locus and in the genes adjacent to *GNAS* at 20q13, in patients with endocrine disorders pseudohypoparathyroidism type 1a and type 1b [174, 175]. This raises an intriguing question about to the extent to which small-scale copy number changes affecting imprinted genes at 20q11-q13.32 or other imprinted genes may occur in tumors, how they may influence expression of these genes, and whether they may affect response of malignant cells to drug treatment. The importance of such fine-scale analysis of imprinted gene clusters was suggested earlier [94]. Recent additions to the CCLE data at the DepMap project site at the Broad Institute [45, 85, 86] and the Cell Line Passports project at Sanger Institute [176] include high-resolution copy number data which these projects have been generating using whole genome and whole exome sequencing information. These new additions of next-generation sequencing data will provide an opportunity for future fine-scale bioinformatic analyses of copy number variation using finely mapped breakpoints within imprinted genes. Such fine-scale analysis of possible intragenic changes may provide a more detailed picture of the effects of copy number changes on expression of imprinted genes and their isoforms in malignant cells.

Our study analyzed the data obtained from tumor cell lines to examine an association between molecular features of imprinted genes and tumor cell response to drug treatment. Some features of cancer cell lines may not be identical to those of primary tumors. A study comparing the features of imprinted genes in GDSC tumor cell lines versus primary TCGA tumors found much more pronounced changes in DNA methylation status and copy number gains in cancer cell lines than in primary tumors [14]. However, multiple studies showed concordance between cancer cell lines and primary tumors in the direction of changes of the copy number, methylation, and/or expression status of many imprinted genes in cancer [2, 8, 14]. These observations provide support for the translational importance of the associations observed in our study between molecular features of imprinted genes and chemoresistance using in vitro data.

## Conclusions

Increased copy number and changes in gene expression and DNA methylation of imprinted genes located in the chromosomal region 20q11-q13.32 were associated with response to multiple chemotherapeutic agents. The influence of this chromosomal region on cancer drug response could be complex and may involve both imprinted and non-imprinted genes located in close proximity to one another. Expression and methylation of a number of imprinted genes in several other genome locations were also associated with drug response.

## Supplementary Information

**Additional file 1: Fig. S1.** The workflow representing the steps of the analysis. Detailed description of each step is provided in the Methods section. CCLE, Cancer Cell Line Encyclopedia. GDSC, Genomics of Drug Sensitivity in Cancer (GDSC1 dataset). *p*_FDR,_ *p* value after FDR adjustment in the analyses of expression and DNA methylation data. *p*_SegmFDR_, *p* values after FDR adjustment using the maximal *p* values from each chromosomal segment in the analysis of copy number data.

**Additional file 2: Table S1.** Names of imprinted genes included in the analyses of copy number, methylation, and expression data.

**Additional file 3: Table S2.** Chromosomal segment (bin) assignment of imprinted genes used for false discovery rate adjustment of the *p* values in the association analysis of copy number data with drug response.

**Additional file 4: Fig. S2.** Distribution of the rounded copy number values of imprinted genes in the 623 cell lines with available CCLE copy number data and GDSC drug response data. Each gene is presented on a separate page.

**Additional file 5: Table S3.** Median and range of the rounded copy number of imprinted genes in the 623 cell lines with available CCLE copy number data and GDSC drug response data.

**Additional file 6: Fig. S3.** Distribution of gene-averaged methylation beta values among 515 imprinted gene regions in 645 cell lines in the pancancer dataset. Shown is the combined distribution of all six gene regions and separate distribution plots for each imprinted gene region category. Horizontal axis represents gene region-averaged methylation beta values, whereas the vertical axis represents gene region counts. The 6 gene regions include TSS1500, TSS200, 5′ UTR (UTR5), 1st exon (EXON1), gene body (GENE BODY), and 3' UTR (UTR3).

**Additional file 7: Table S4.** Results of Spearman correlation analysis of continuous copy number values of the imprinted genes with log(IC50) satisfying *p*_SegmFDR_ < 0.05 and Spearman |ρ| > 0.25. Sample size, number of cell lines with available data used in correlation analysis. Spearman ρ, Spearman correlation coefficient. The results are sorted by the absolute value of |ρ|. *p*0, *p* value prior to FDR adjustment. Segment, bin used for grouping imprinted genes according to their chromosomal location for FDR adjustment of the *p* values. Bin assignment of all imprinted genes is provided in Additional file 3: Table S2. max original segment *p*, maximal *p* value (prior to FDR adjustment) among all imprinted genes in a given chromosomal segment for a given agent. During the FDR adjustment each segment–agent pair was presented once by the maximal *p* value of all genes assigned to that segment in the correlation of their copy number with log(IC50) of that agent. *p*_SegmFDR_, *p* values after FDR adjustment using the maximal *p* values from each chromosomal segment. All correlations presented in the table also satisfied FDR-adjusted *p* < 0.05 if considering all genes independently, without grouping them into segments (*p*_FDR_ < 0.05). Drug response data source, dataset (GDSC or CCLE) from which the drug response values were obtained.

**Additional file 8: Table S5.** Results of Spearman correlation analysis of expression of imprinted genes with log(IC50) satisfying *p*_FDR_ < 0.05 and Spearman |ρ| > 0.3. Spearman ρ, Spearman correlation coefficient. The results are sorted by the absolute value of |ρ|. *p*_0_, *p* value prior to FDR adjustment. *p*_FDR_, *p* value after FDR adjustment. Sample size, number of cell lines with available data used in correlation analysis. Drug response data source, dataset (GDSC or CCLE) from which the drug response values were obtained.

**Additional file 9: Table S6.** Methylation of imprinted gene regions associated with log(IC50) satisfying |ρ| > 0.3 and *p*_FDR_ < 0.05. Spearman ρ, Spearman correlation coefficient. The results are sorted by the absolute value of |ρ|. *p*_0_, *p* value prior to FDR adjustment. *p*_FDR_, *p* value after FDR adjustment. Drug response data source, dataset (GDSC or CCLE) from which the drug response values were obtained. Sample size, number of cell lines with available data used in correlation analysis. Cytoband, chromosomal region location according to the UCSC genome annotation database for the hg19 (GRCh37) assembly of the human genome based on the probe coordinates in the Illumina Infinium HumanMethylation 450K BeadChip annotation. The 6 gene regions include TSS1500, TSS200, 5′ UTR (UTR5), 1st exon (EXON1), gene body (GENE BODY), and 3' UTR (UTR3).

**Additional file 10: Table S7.** Spearman and Pearson correlation among copy number values of imprinted genes in the chromosomal region 20q11-q13 and non-imprinted cancer genes *AURKA*, *MYBL2*, and *ZNF217*. Shown are associations of copy number of non-imprinted and imprinted genes with copy number values of imprinted genes at 20q11-q13 listed in Table 1 whose copy number values were associated with drug response (*p*_SegmFDR_ < 0.05 and Spearman |ρ| > 0.3). ρ, Spearman correlation coefficient; *r*, Pearson correlation coefficient.

**Additional file 11: Table S8.** Correlations of expression of imprinted and non-imprinted genes at 20q11-q13.32 with log(IC50) satisfying Spearman |ρ| > 0.25.All correlations satisfying |ρ| > 0.25 had *p*_FDR_ < 0.1. An asterisk (*) shows correlations satisfying more stringent criteria of Spearman |ρ| > 0.3 and *p*_FDR_ < 0.1. Correlations are highlighted in shades of pink or blue according to the direction of association (positive or negative, respectively). Sample size, number of cell lines with available data used in correlation analysis. Spearman ρ, Spearman correlation coefficient. *p*_0_, *p* value prior to FDR adjustment. *p*_FDR_, *p* value after FDR adjustment.

**Additional file 12: Fig. S4.** Scatterplots of the ex vivo log(IC50) drug response measures from the Beat AML 1.0 cohort vs log_2_ RPKM expression of the genes at 20q11-q13.32 for the correlations listed in Additional file 11:Table S8, which satisfy Spearman |ρ| > 0.25 and *p*_FDR_ < 0.1. Shown are the following gene–drug pairs: A. *SGK2* - nilotinib. B. *L3MBTL1 *- nilotinib. C. *NNAT *- TG101348 (fedratinib). D. *GNAS *- TG101348 (fedratinib). E. *GNAS* - lestaurtinib (CEP-701). F. *SGK2* - panobinostat. G. *GNAS* - nilotinib. H. *ZNF217 *- lenalidomide. I. *GNAS* – lenalidomide. J. *SGK2*- lenalidomide. Samples carrying *ASXL1* mutations are shown in red and are listed as ASXL1 mutation in the figure legend. Samples with a reported cytogenetic loss of all or a part of the 20q11-q13.32 region are shown as diamonds with black borders and are listed as 20q deletion in the figure legend. Spearman ρ, Spearman correlation coefficient; Pearson *r*, Pearson correlation coefficient. The dashed line indicates the linear regression line.

**Additional file 13: Fig. S5.** Boxplots of the distribution, by cancer category, of continuous copy number values of select genes in the 20q11-q13.32 region, for the 623 cell lines with available copy number data.

**Additional file 14: Fig. S6.** Boxplots of the distribution of gene expression measures among cancer categories in the 645 cancer cell lines. Shown are select genes from Additional file 8:Table S5 which were discussed in the text and whose expression was significantly associated drug response. *BLCAP* and *HM13* are located in the 20q11-q13.32 region (20q11.23 and 20q11.21, respectively). The remaining genes shown in the figure are located in other chromosomal regions (*CPA4* at 7q32.2, *DNMT1* at 19p13.2, *PHLDA2* at 11p15.4, and *RB1* at 13q14.2; Additional file 2:Table S1).

**Additional file 15: Fig. S7.** Boxplots of the distribution of logIC50 measures of drug response among cancer categories in the 645 cancer cell lines. Shown are examples of agents from Table 1 and Additional file 8:Table S5 whose expression was significantly associated with copy number and/or expression of imprinted genes.

## Data Availability

All CCLE, GDSC, and Beat AML 1.0 data used in this project are publicly available online. Information about their access is provided in the Methods section. The list of 645 cell lines matched between the CCLE and GDSC datasets, which were analyzed in this study, is publicly available online [79].

## Abbreviations

5-FU: 5-Fluorouracil
ALL: Acute lymphocytic leukemia
AML: Acute myeloid leukemia
ANO1: Anoctamin 1
BLADDER: Bladder cancer
BLCAP: Bladder cancer-associated protein
BREAST: Breast cancer
CESC: Cervical squamous cell carcinoma and endocervical adenocarcinoma
CCLE: Cancer Cell Line Encyclopedia
CDK: Cyclin-dependent kinase
CLLE: Chronic lymphocytic leukemia
COAD/READ: Colon adenocarcinoma and rectum adenocarcinoma
CPA4: Carboxypeptidase A4
DA: Duodenal adenocarcinoma
DepMap: Cancer Dependency Map
DLBCL: Diffuse large B cell lymphoma
DLX5: Distal-less homeobox 5 protein
DNMT1: DNA methyltransferase 1
EC: Esophageal cancer
EXON1: 1st exon
FDR: False discovery rate
GDSC: Genomics of Drug Sensitivity in Cancer
GEO: Gene Expression Omnibus
GLIOMA: Glioma brain tumors
GNAS: Guanine nucleotide-binding protein: alpha-stimulating
HDAC: Histone deacetylase
HL: Hodgkin lymphoma
HNSC: Head and neck squamous cell carcinoma
KSP: Kinesin spindle protein
LAML: Acute myeloid leukemia
LCML: Chronic myelogenous leukemia
LIHC: Liver hepatocellular carcinoma
LXR: Liver X receptor
MATBCL: Mature B cell lymphoma
MB: Medulloblastoma
MEL: Melanoma
MESO: Mesothelioma
MILD: Multi-locus imprinting disturbance
MISC: Other miscellaneous categories of cancer including rare cancers or cancers with unspecified information
MDS: Myelodysplastic syndrome
MM: Multiple myeloma
NB: Neuroblastoma
ncRNA: Noncoding RNA
NCBI: National Center for Biotechnology Information
NNAT: Neuronatin
NLR: Nucleotide-binding and leucine-rich repeat receptor
NLRP2: NLR family pyrin domain containing 2
NSCLC: Non-small cell lung cancer
OCT: Organic cation transporter
OVARIAN: Ovarian cancer
p_0_: *p* value prior to FDR adjustment
PAAD: Pancreatic adenocarcinoma
*p*_FDR_: *p* value after FDR adjustment which considered all imprinted genes independently
PHLDA2: Pleckstrin homology-like domain: family A, member 2
PLAGL1: Pleiomorphic adenoma gene-like 1
PNET: Primitive neuroectodermal tumors
PRAD: Prostate adenocarcinoma
*p*_SegmFDR_: *p* value after FDR adjustment using the highest *p* values from each chromosomal segment
RCC: Renal cell carcinoma
SAR: Sarcoma
SCLC: Small cell lung cancer
Shh: Sonic Hedgehog
STAD: Stomach adenocarcinoma
TCGA: The Cancer Genome Atlas
TCL: T cell lymphoma
THCA: Thyroid carcinoma
UCEC: Uterine corpus endometrial carcinoma
UTR3: 3′ UTR
UTR5: 5′ UTR
