# Supplementary material for: Increased copy number of imprinted genes in the chromosomal region 20q11-q13.32 is associated with resistance to antitumor agents in cancer cell lines
Source: Clin Epigenetics. 2022 Dec 2;14:161. doi: 10.1186/s13148-022-01368-7 (PMC9716673; doi:10.1186/s13148-022-01368-7)
Supplement: Supplementary file 1 — Additional file 1: Fig. S1. The workflow representing the steps of the analysis. Detailed description of each step is provided in the Methods section. CCLE, Cancer Cell Line Encyclopedia. GDSC, Genomics of Drug Sensitivity in Cancer (GDSC1 dataset). pFDR, p value after FDR adjustment in the analyses of expression and DNA methylation data. pSegmFDR, p values after FDR adjustment using the maximal p values from each chromosomal segment in the analysis of copy number data. [file 13148_2022_1368_MOESM1_ESM.pdf]

Data Collection for Imprinted Genes (protein-coding imprinted genes and imprinted ncRNA)

Collection of imprinted genes from the Catalogue of Imprinted Genes, GenImprint and biomedical publications

Included all human genes reported to be imprinted in embryonic or adult somatic tissues, placenta, embryonic stem cells, or iPSCs

198 imprinted genes with matching molecular data

Molecular and drug response data for 645 matched cancer cell lines from CCLE and GDSC

275 log(IC50) measures for 255 agents (CCLE and GDSC) 645 cell lines 23 tumor types

Continuous copy number data (CCLE) 198 imprinted genes 623 cell lines 22 tumor types

RPKM RNA-seq expression data (CCLE) 108 imprinted genes 645 cell lines 23 tumor types

Illumina 450K Beadchip array probe methylation beta-values (GDSC) 113 imprinted genes 645 cell lines 23 tumor types

Gene region-averaged methylation beta-values computed for TSS1500, TSS200, 5' UTR, 1st exon, gene body, and 3'UTR

Distributions of continuous and discreet copy number values and continuous expression and gene region-averaged methylation values were examined.

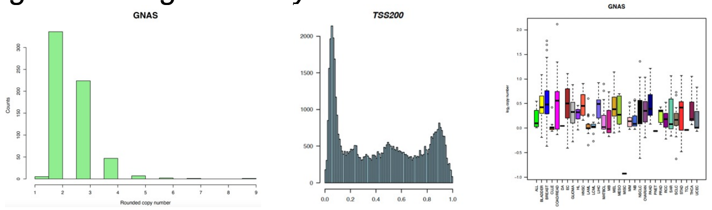

Association Analysis of Drug Response (logIC50) with Molecular Measures of Imprinted Genes

Only significant association results for pancancer data were presented.

No associations of copy number data in individual cancer categories satisfied $p_{SegmFDR} < 0.5$ due to a very large number of tests (169365).

Copy number (continuous values) $p_{SegmFDR} < 0.5$ in Spearman correlation

Only imprinted genes on 20q11-q13.32 satisfied $|\rho| > 0.3$ and $p_{SegmFDR} < 0.5$

Association of molecular measures for 3 additional non-imprinted genes *MYBL2*, *AURKA*, and *ZNF217* on 20q11-q13.32 was examined for possible confounding of associations in that region

Gene expression $p_{FDR} < 0.5$ in Spearman correlation

Imprinted genes on 20q11-q13.32 and in other chromosomal regions satisfied $|\rho| > 0.3$ and $p_{FDR} < 0.5$

DNA methylation of gene regions $p_{FDR} < 0.5$ in Spearman correlation

Imprinted genes on 20q11-q13.32 and in other chromosomal regions satisfied $|\rho| > 0.3$ and $p_{FDR} < 0.5$

Concordance was examined among the top significant association results of drug response with copy number, gene expression, and DNA methylation, and among copy number, gene expression, and gene region-averaged DNA methylation of imprinted genes.

Validation of associations of gene expression at 20q11-q13.32 with *ex vivo drug* response using patient sample data from the Beat AML 1.0 cohort

Fig. S1
