# Supplementary material for: Increased copy number of imprinted genes in the chromosomal region 20q11-q13.32 is associated with resistance to antitumor agents in cancer cell lines
Source: Clin Epigenetics. 2022 Dec 2;14:161. doi: 10.1186/s13148-022-01368-7 (PMC9716673; doi:10.1186/s13148-022-01368-7)
Supplement: Supplementary file 2 — Additional file 2: Table S1. Names of imprinted genes included in the analyses of copy number, methylation, and expression data. [file 13148_2022_1368_MOESM2_ESM.pdf]

Table S1. Names of imprinted genes included in the analyses of copy number, methylation, and expression data

Gene	Copy number analysis	Methylation analysis	Expression analysis	Gene category	Location[a]	References[c]
AIM1	AIM1	AIM1	AIM1	protein-coding	6q21	[1-6]
AIRN	AIRN	Not included	AIRN	ncRNA	6q25	[1-3, 7]
ANO1	ANO1	ANO1	ANO1	protein-coding	11q13.3	[1-5, 8, 9]
ATP10A	ATP10A	ATP10A	ATP10A	protein-coding	15q12	[1-5, 8]
BLCAP	BLCAP	BLCAP	BLCAP	protein-coding	20q11.23	[1-5, 8]
CALCR	CALCR	CALCR	CALCR	protein-coding	7q21.3	[1-4, 8]
CDKN1C	CDKN1C	CDKN1C	CDKN1C	protein-coding	11p15.4	[1-5, 7, 8, 10]
CPA4	CPA4	CPA4	CPA4	protein-coding	7q32.2	[1-5, 8, 11]
DCN	DCN	DCN	DCN	protein-coding	12q21.33	[1-3, 12]
DDC	DDC	DDC	DDC	protein-coding	7p12.2-p12.1	[1-5, 8]
DGCR6	DGCR6	DGCR6	DGCR6	protein-coding	22q11.21	[1-3, 5, 8]
DIO3	DIO3	DIO3	DIO3	protein-coding	14q32.31	[1-4, 12]
DIRAS3	DIRAS3	DIRAS3	DIRAS3	protein-coding	1p31.3	[1-5, 8, 10, 11]
DLGAP2	DLGAP2	DLGAP2	DLGAP2	protein-coding	8p23.3	[1-5, 8, 13]
DLK1	DLK1	DLK1	DLK1	protein-coding	14q32.2	[1-3, 5, 8, 10-12]
DLX5	DLX5	DLX5	DLX5	protein-coding	7q22	[1-3, 5, 8]
DNMT1	DNMT1	DNMT1	DNMT1	protein-coding	19p13.2	[1-3, 5, 6]
FAM50B	FAM50B	FAM50B	FAM50B	protein-coding	6p25.2	[1-3, 5, 8, 11]
GABRA5	GABRA5	GABRA5	GABRA5	protein-coding	15q12	[1-3]
GABRB3	GABRB3	GABRB3	GABRB3	protein-coding	15q12	[1-3, 10, 14]
GABRG3	GABRG3	GABRG3	GABRG3	protein-coding	15q12	[1-3, 15]
GDAP1L1	GDAP1L1	GDAP1L1	GDAP1L1	protein-coding	20q12	[1-3, 5, 16]
GLIS3	GLIS3	GLIS3	GLIS3	protein-coding	9p24.2	[1-3, 5, 8]
GNAS	GNAS	GNAS	GNAS	protein-coding	20q13.32	[1-3, 5, 8, 17]
GNASAS	GNAS-AS1	GNASAS	GNAS-AS1	ncRNA	20q13.32	[1-3, 5, 8, 12, 17]
GPR1	GPR1	GPR1	GPR1	protein-coding	2q33.3	[1-3, 5, 8]
GRB10	GRB10	GRB10	GRB10	protein-coding	7p12.1	[1-3, 5, 8, 10, 11]
H19	H19	H19	H19	ncRNA	11p15.5	[1-3, 5, 8, 10, 11]
HM13	HM13	HM13	HM13	protein-coding	20q11.21	[1-3, 8]
HTR2A	HTR2A	HTR2A	HTR2A	protein-coding	13q14.2	[1-3]
HYMAI	HYMAI	HYMAI	Not included	ncRNA	6q24.2	[1-3, 5]
IGF2	IGF2[b]	IGF2	IGF2	protein-coding	11p15.5	[1-5, 8, 10, 11]
IGF2AS	IGF2-AS	IGF2AS	IGF2-AS	ncRNA	11p15.5	[1-5, 11]
IGF2R	IGF2R	IGF2R	IGF2R	protein-coding	6q25.3	[1-3, 8]
INPP5F	INPP5F	INPP5F	INPP5F	protein-coding	10q26.11	[1-5, 8, 11]
INS	INS[b]	INS	INS	protein-coding	11p15.5	[1-5]
IPW	IPW	IPW	Not included	ncRNA	15q11.2	[1-5, 8]

KCNK9	KCNK9	KCNK9	KCNK9	protein-coding	8q24.3	[1-5, 8, 13]
KCNQ1	KCNQ1	KCNQ1	KCNQ1	protein-coding	11p15.4-p15.5	[1-5, 7, 8, 11]
KCNQ1DN	KCNQ1DN	KCNQ1DN	KCNQ1DN	ncRNA	11p15.4-p15.5	[1-5]
KCNQ1OT1	KCNQ1OT1	KCNQ1OT1	KCNQ1OT1	ncRNA	11p15.5	[1-5, 7, 8, 10, 12]
KLF14	KLF14	KLF14	KLF14	protein-coding	7q32.2	[1-5, 8]
L3MBTL	L3MBTL1	L3MBTL	L3MBTL1	protein-coding	20q13.12	[1-5, 8, 10, 11, 16]
LIN28B	LIN28B	LIN28B	LIN28B	protein-coding	6q16.3-q21	[1-5, 8]
LRRTM1	LRRTM1	LRRTM1	LRRTM1	protein-coding	2p12	[1-5, 8]
MAGEL2	MAGEL2	MAGEL2	MAGEL2	protein-coding	15q11.2	[1-5, 8, 10, 11, 15]
MAGI2	MAGI2	MAGI2	MAGI2	protein-coding	7q21.11	[1-5, 8, 11]
MEG3	MEG3	MEG3	MEG3	ncRNA	14q32.2	[1-5, 8, 10-12]
MEG8	MEG8	MEG8	MEG8	ncRNA	14q32.2-q32.31	[1-5, 11]
MEST	MEST	MEST	MEST	protein-coding	7q32.2	[1-5, 8, 10, 11]
MESTIT1	MESTIT1	MESTIT1	Not included	ncRNA	7q32.2	[1-5, 8]
MIMT1	MIMT1	MIMT1	MIMT1	ncRNA	19q13.43	[1-5, 8]
MIR134	MIR134	MIR134	MIR134	ncRNA	14q32.31	[1-4, 18, 19]
MIR184	MIR184	MIR184	MIR184	ncRNA	15q25.1	[1-3]
MIR296	MIR296	MIR296	MIR296	ncRNA	20q13.32	[1-5, 8]
MIR298	MIR298	MIR298	MIR298	ncRNA	20q13.32	[1-5, 8]
MIR371A	MIR371A	MIR371	MIR371A	ncRNA	19q13.42	[1-5]
MIR379	MIR379	Not included	Not included	ncRNA	14q32.31	[1-3, 19-21]
MIR409	MIR409	Not included	Not included	ncRNA	14q32.31	[1-3, 19-21]
MIR410	MIR410	MIR410	MIR410	ncRNA	14q32.31	[1-3, 19]
MIR483	MIR483	MIR483	MIR483	ncRNA	11p15.5	[4]
MIR487B	MIR487B	Not included	Not included	ncRNA	14q32.31	[1-3, 19-21]
MIR517A	MIR517A	Not included	Not included	ncRNA	19q13.42	[1-3, 18, 22, 23]
MIR656	MIR656	Not included	Not included	ncRNA	14q32.31	[1-3, 19-21]
MIR675	MIR675	MIR675	Not included	ncRNA	11p15.5	[1-4, 24]
MKRN3	MKRN3	MKRN3	MKRN3	protein-coding	15q11.2	[1-5, 8, 10]
NAA60	NAA60	NAT15	NAA60	protein-coding	16p13.3	[1-5, 8]
NAP1L5	NAP1L5	NAP1L5	NAP1L5	protein-coding	4q22.1	[1-5, 8, 10, 11]
NDN	NDN	NDN	NDN	protein-coding	15q11.2	[1-5, 8, 10-12, 15]
NLRP2	NLRP2	NLRP2	NLRP2	protein-coding	19q13.42	[1-5, 8, 10]
NNAT	NNAT	NNAT	NNAT	protein-coding	20q11.23	[1-5, 8, 10]
NPAP1	NPAP1	C15orf2	NPAP1	protein-coding	15q11.2	[1-5]
NTM	NTM	NTM	NTM	protein-coding	11q25	[1-5, 8, 11]
OSBPL5	OSBPL5	OSBPL5	OSBPL5	protein-coding	11p15.4	[1-5, 8]
PAR1	PAR1	Not included	Not included	ncRNA	15q11.2	[1-3, 25, 26]
PAR5	PAR5	Not included	Not included	ncRNA	15q11.2	[1-3, 26]
PEG3	PEG3	PEG3	PEG3	protein-coding	19q13.43	[1-5, 10, 11]

PEG10	PEG10	PEG10	PEG10	protein-coding	7q21.3	[1-5, 8, 10, 11]
PHACTR2	PHACTR2	PHACTR2	PHACTR2	protein-coding	6q24.2	[1-5, 8]
PHLDA2	PHLDA2	PHLDA2	PHLDA2	protein-coding	11p15.4	[1-5, 7, 8]
PLAGL1	PLAGL1	PLAGL1	PLAGL1	protein-coding	6q24.2	[1-5, 8-11]
PPP1R9A	PPP1R9A	PPP1R9A	PPP1R9A	protein-coding	7q21.3	[1-5, 8]
PSIMCT-1	PSIMCT-1	PSIMCT-1	Not included	pseudogene	20q11.21	[1-5]
PWRN1	PWRN1	PWRN1	PWRN1	ncRNA	15q11.2	[1-4, 27]
RASGRF1	RASGRF1	RASGRF1	RASGRF1	protein-coding	15q25.1	[1-3, 28]
RB1	RB1	RB1	RB1	protein-coding	13q14.2	[1-5, 8, 10, 29]
RBP5	RBP5	RBP5	RBP5	protein-coding	12p13.31	[1-5, 8]
RNU5D-1	RNU5D-1	RNU5D	RNU5D-1	ncRNA	1p34.1	[1-5, 8]
RTL1	RTL1	RTL1	RTL1	protein-coding	14q32.2-q32.31	[1-5, 8, 12]
SGCE	SGCE	SGCE	SGCE	protein-coding	7q21.3	[1-5, 8]
SGK2	SGK2	SGK2	SGK2	protein-coding	20q13.12	[1-5, 8, 16]
SLC22A18	SLC22A18	SLC22A18	SLC22A18	protein-coding	11p15.4	[1-5, 7, 8]
SLC22A18AS	SLC22A18AS	SLC22A18AS	SLC22A18AS	ncRNA	11p15.4	[1-3, 7]
SLC22A2	SLC22A2	SLC22A2	SLC22A2	protein-coding	6q25.3	[1-5, 30, 31]
SLC22A3	SLC22A3	SLC22A3	SLC22A3	protein-coding	6q25.3	[1-5, 7, 8, 10, 31]
SNORD107	SNORD107	SNORD107	Not included	ncRNA	15q11.2	[1-5, 8]
SNORD108	SNORD108	SNORD108	SNORD108	ncRNA	15q11.2	[1-5]
SNORD109A	SNORD109A	SNORD109A	SNORD109A	ncRNA	15q11.2	[1-5, 32]
SNORD109B	SNORD109B	SNORD109B	SNORD109B	ncRNA	15q11.2	[1-5, 32]
SNORD115-1	SNORD115-1	Not included	Not included	ncRNA	15q11.2	[1-5, 32]
SNORD115-10	SNORD115-10	Not included	Not included	ncRNA	15q11.2	[1-5, 32]
SNORD115-11	SNORD115-11	Not included	Not included	ncRNA	15q11.2	[1-5, 32]
SNORD115-12	SNORD115-12	Not included	Not included	ncRNA	15q11.2	[1-5, 32]
SNORD115-13	SNORD115-13	Not included	Not included	ncRNA	15q11.2	[1-5, 32]
SNORD115-14	SNORD115-14	Not included	Not included	ncRNA	15q11.2	[1-5, 32]
SNORD115-15	SNORD115-15	Not included	Not included	ncRNA	15q11.2	[1-5, 32]
SNORD115-16	SNORD115-16	Not included	Not included	ncRNA	15q11.2	[1-5, 32]
SNORD115-17	SNORD115-17	Not included	Not included	ncRNA	15q11.2	[1-5, 32]
SNORD115-18	SNORD115-18	Not included	Not included	ncRNA	15q11.2	[1-5, 32]
SNORD115-19	SNORD115-19	Not included	Not included	ncRNA	15q11.2	[1-5, 32]
SNORD115-2	SNORD115-2	Not included	Not included	ncRNA	15q11.2	[1-5, 32]
SNORD115-20	SNORD115-20	Not included	Not included	ncRNA	15q11.2	[1-5, 32]
SNORD115-21	SNORD115-21	Not included	Not included	ncRNA	15q11.2	[1-5, 32]
SNORD115-22	SNORD115-22	Not included	Not included	ncRNA	15q11.2	[1-5, 32]
SNORD115-23	SNORD115-23	Not included	Not included	ncRNA	15q11.2	[1-5, 32]
SNORD115-24	SNORD115-24	Not included	Not included	ncRNA	15q11.2	[1-5, 32]
SNORD115-25	SNORD115-25	Not included	Not included	ncRNA	15q11.2	[1-5, 32]

SNORD115-26	SNORD115-26	Not included	Not included	ncRNA	15q11.2	[1-5, 32]
SNORD115-27	SNORD115-27	Not included	Not included	ncRNA	15q11.2	[1-5, 32]
SNORD115-28	SNORD115-28	Not included	Not included	ncRNA	15q11.2	[1-5, 32]
SNORD115-29	SNORD115-29	Not included	Not included	ncRNA	15q11.2	[1-5, 32]
SNORD115-3	SNORD115-3	Not included	Not included	ncRNA	15q11.2	[1-5, 32]
SNORD115-30	SNORD115-30	Not included	Not included	ncRNA	15q11.2	[1-5, 32]
SNORD115-31	SNORD115-31	Not included	Not included	ncRNA	15q11.2	[1-5, 32]
SNORD115-32	SNORD115-32	Not included	Not included	ncRNA	15q11.2	[1-5, 32]
SNORD115-33	SNORD115-33	Not included	Not included	ncRNA	15q11.2	[1-5, 32]
SNORD115-34	SNORD115-34	Not included	Not included	ncRNA	15q11.2	[1-5, 32]
SNORD115-35	SNORD115-35	Not included	Not included	ncRNA	15q11.2	[1-5, 32]
SNORD115-36	SNORD115-36	Not included	Not included	ncRNA	15q11.2	[1-5, 32]
SNORD115-37	SNORD115-37	Not included	Not included	ncRNA	15q11.2	[1-5, 32]
SNORD115-38	SNORD115-38	Not included	Not included	ncRNA	15q11.2	[1-5, 32]
SNORD115-39	SNORD115-39	Not included	Not included	ncRNA	15q11.2	[1-5, 32]
SNORD115-4	SNORD115-4	Not included	Not included	ncRNA	15q11.2	[1-5, 32]
SNORD115-40	SNORD115-40	Not included	Not included	ncRNA	15q11.2	[1-5, 32]
SNORD115-41	SNORD115-41	Not included	Not included	ncRNA	15q11.2	[1-5, 32]
SNORD115-42	SNORD115-42	Not included	Not included	ncRNA	15q11.2	[1-5, 32]
SNORD115-43	SNORD115-43	Not included	Not included	ncRNA	15q11.2	[1-5, 32]
SNORD115-44	SNORD115-44	Not included	Not included	ncRNA	15q11.2	[1-5, 32]
SNORD115-45	SNORD115-45	Not included	Not included	ncRNA	15q11.2	[1-5, 32]
SNORD115-47	SNORD115-47	Not included	Not included	ncRNA	15q11.2	[1-5, 32]
SNORD115-48	SNORD115-48	SNORD115-48	SNORD115-48	ncRNA	15q11.2	[1-5, 32]
SNORD115-5	SNORD115-5	Not included	Not included	ncRNA	15q11.2	[1-5, 32]
SNORD115-6	SNORD115-6	Not included	Not included	ncRNA	15q11.2	[1-5, 32]
SNORD115-7	SNORD115-7	Not included	Not included	ncRNA	15q11.2	[1-5, 32]
SNORD115-8	SNORD115-8	Not included	Not included	ncRNA	15q11.2	[1-5, 32]
SNORD115-9	SNORD115-9	Not included	Not included	ncRNA	15q11.2	[1-5, 32]
SNORD116-1	SNORD116-1	Not included	Not included	ncRNA	15q11.2	[1-5, 32]
SNORD116-10	SNORD116-10	Not included	Not included	ncRNA	15q11.2	[1-5, 32]
SNORD116-11	SNORD116-11	Not included	Not included	ncRNA	15q11.2	[1-5, 32]
SNORD116-12	SNORD116-12	Not included	Not included	ncRNA	15q11.2	[1-5, 32]
SNORD116-13	SNORD116-13	Not included	Not included	ncRNA	15q11.2	[1-5, 32]
SNORD116-14	SNORD116-14	Not included	Not included	ncRNA	15q11.2	[1-5, 32]
SNORD116-15	SNORD116-15	Not included	Not included	ncRNA	15q11.2	[1-5, 32]
SNORD116-16	SNORD116-16	Not included	Not included	ncRNA	15q11.2	[1-5, 32]
SNORD116-17	SNORD116-17	Not included	Not included	ncRNA	15q11.2	[1-5, 32]
SNORD116-18	SNORD116-18	Not included	Not included	ncRNA	15q11.2	[1-5, 32]
SNORD116-19	SNORD116-19	Not included	Not included	ncRNA	15q11.2	[1-5, 32]

SNORD116-2	SNORD116-2	Not included	Not included	ncRNA	15q11.2	[1-5, 32]
SNORD116-20	SNORD116-20	Not included	Not included	ncRNA	15q11.2	[1-5, 32]
SNORD116-21	SNORD116-21	Not included	Not included	ncRNA	15q11.2	[1-5, 32]
SNORD116-22	SNORD116-22	Not included	Not included	ncRNA	15q11.2	[1-5, 32]
SNORD116-23	SNORD116-23	Not included	Not included	ncRNA	15q11.2	[1-5, 32]
SNORD116-24	SNORD116-24	Not included	Not included	ncRNA	15q11.2	[1-5, 32]
SNORD116-25	SNORD116-25	Not included	Not included	ncRNA	15q11.2	[1-5, 32]
SNORD116-26	SNORD116-26	Not included	Not included	ncRNA	15q11.2	[1-5, 32]
SNORD116-27	SNORD116-27	Not included	Not included	ncRNA	15q11.2	[1-5, 32]
SNORD116-28	SNORD116-28	Not included	Not included	ncRNA	15q11.2	[1-5, 32]
SNORD116-29	SNORD116-29	Not included	Not included	ncRNA	15q11.2	[1-5, 32]
SNORD116-3	SNORD116-3	Not included	Not included	ncRNA	15q11.2	[1-5, 32]
SNORD116-4	SNORD116-4	Not included	Not included	ncRNA	15q11.2	[1-5, 32]
SNORD116-5	SNORD116-5	Not included	Not included	ncRNA	15q11.2	[1-5, 32]
SNORD116-6	SNORD116-6	Not included	Not included	ncRNA	15q11.2	[1-5, 32]
SNORD116-7	SNORD116-7	Not included	Not included	ncRNA	15q11.2	[1-5, 32]
SNORD116-8	SNORD116-8	Not included	Not included	ncRNA	15q11.2	[1-5, 32]
SNORD116-9	SNORD116-9	Not included	Not included	ncRNA	15q11.2	[1-5, 32]
SNORD64	SNORD64	SNORD64	SNORD64	ncRNA	15q11.2	[1-5, 32]
SNRPN	SNRPN	SNRPN	SNRPN	protein-coding	15q11.2	[1-5, 8, 10, 32]
SNURF	SNURF	SNURF	SNURF	protein-coding	15q11.2	[1-5, 8, 32]
TCEB3C	TCEB3C	TCEB3C	TCEB3C	protein-coding	18q21.1	[1-5, 33, 34]
TFPI2	TFPI2	TFPI2	TFPI2	protein-coding	7q21.3	[1-5, 8, 12]
TH	TH	TH	TH	protein-coding	11p15.5	[1-3]
TP73	TP73	TP73	TP73	protein-coding	1p36.32	[1-5, 8]
UBE3A	UBE3A	UBE3A	UBE3A	protein-coding	15q11.2	[1-5, 8]
USP29	USP29	USP29	USP29	protein-coding	19q13.43	[1-3, 10]
VTRNA2-1	VTRNA2-1	MIR886	VTRNA2-1	nc RNA	5q31.1	[1-3, 5, 35]
WIF1	WIF1	WIF1	WIF1	protein-coding	12q14.3	[1-3, 8]
WT1	WT1	WT1	WT1	protein-coding	11p13	[1-5, 8]
WT1AS	WT1-AS	Not included	Not included	ncRNA	11p13	[1-3, 5, 36]
ZC3H12C	ZC3H12C	ZC3H12C	ZC3H12C	protein-coding	11q22.3	[1-5, 8]
ZDBF2	ZDBF2	ZDBF2	ZDBF2	protein-coding	2q33.3	[1-5, 10]
ZFAT	ZFAT	ZFAT	ZFAT	protein-coding	8q24.22	[1-5, 8]
ZFAT-AS1	ZFAT-AS1	ZFATAS	ZFAT-AS1	ncRNA	8q24.22	[1-5, 8]
ZIM2	ZIM2	ZIM2	ZIM2	protein-coding	19q13.43	[1-5, 8]
ZIM3	ZIM3	ZIM3	ZIM3	protein-coding	19q13.43	[1-3, 10]
ZNF215	ZNF215	ZNF215	ZNF215	protein-coding	11p15.4	[1-3]
ZNF331	ZNF331	ZNF331	ZNF331	protein-coding	19q13.42	[1-4, 8]
ZNF597	ZNF597	ZNF597	ZNF597	protein-coding	16p13.3	[1-5, 8]

Listed are the genes with available molecular data from the CCLE and GDSC resources that had been reported to be imprinted in embryonic or adult somatic tissues, placenta, embryonic stem cells, or induced pluripotent stem cells (iPSCs).

[a] Chromosomal location is provided according to the data in the Catalog of Imprinted Genes [1-3], Bonaldi et al. [4], and additional publications listed among the references. For those imprinted genes that had minor discrepancies among their reported chromosomal location from different sources, their chromosomal location was reported according to GeneCards [37].

[b] Copy number of the entire INS-IGF2 locus was also provided in the CCLE dataset and analyzed.

[c] The Geneimprint resource [5] was described in [38].

Not included, indicates that a gene was not included in the analysis of a particular molecular category.

Gene category is provided according to the information reported by Bonaldi et al. [4] and/or GeneCards [37].

References for Supplementary Table 1

1. Morison I. Catalogue of Imprinted Genes. 2021. Available from: http://www.otago.ac.nz/IGC. Accessed: 22 July 2021
2. Morison IM, Paton CJ, Cleverley SD. The imprinted gene and parent-of-origin effect database. Nucleic Acids Res. 2001;29:275-6.
3. Morison IM, Ramsay JP, Spencer HG. A census of mammalian imprinting. Trends Genet. 2005;21:457-65.
4. Bonaldi A, Kashiwabara A, de Araujo ES, Pereira LV, Paschoal AR, Andozia MB et al. Mining novel candidate imprinted genes using genome-wide methylation screening and literature review. Epigenomes. 2017;1.
5. Geneimprint. Available from: http://www.geneimprint.org/. Accessed: 8 April 2019
6. Das R, Lee YK, Strogantsev R, Jin S, Lim YC, Ng PY et al. *DNMT1* and *AIM1* Imprinting in human placenta revealed through a genome-wide screen for allele-specific DNA methylation. BMC Genomics. 2013;14:685.
7. Monk D, Arnaud P, Apostolidou S, Hills FA, Kelsey G, Stanier P et al. Limited evolutionary conservation of imprinting in the human placenta. Proc Natl Acad Sci U S A. 2006;103:6623-8.
8. Babak T, DeVeale B, Tsang EK, Zhou Y, Li X, Smith KS et al. Genetic conflict reflected in tissue-specific maps of genomic imprinting in human and mouse. Nat Genet. 2015;47:544-9.
9. Smeester L, Yosim AE, Nye MD, Hoyo C, Murphy SK, Fry RC. Imprinted genes and the environment: links to the toxic metals arsenic, cadmium, lead and mercury. Genes (Basel). 2014;5:477-96.
10. Anwar SL, Krech T, Hasemeier B, Schipper E, Schweitzer N, Vogel A et al. Loss of DNA methylation at imprinted loci is a frequent event in hepatocellular carcinoma and identifies patients with shortened survival. Clin Epigenetics. 2015;7:110.
11. Baran Y, Subramaniam M, Biton A, Tukiainen T, Tsang EK, Rivas MA et al. The landscape of genomic imprinting across diverse adult human tissues. Genome Res. 2015;25:927-36.
12. Ribarska T, Bastian KM, Koch A, Schulz WA. Specific changes in the expression of imprinted genes in prostate cancer--implications for cancer progression and epigenetic regulation. Asian J Androl. 2012;14:436-50.
13. Luedi PP, Dietrich FS, Weidman JR, Bosko JM, Jirtle RL, Hartemink AJ. Computational and experimental identification of novel human imprinted genes. Genome Res. 2007;17:1723-30.
14. Bird LM. Angelman syndrome: review of clinical and molecular aspects. Appl Clin Genet. 2014;7:93-104.
15. Sharp AJ, Migliavacca E, Dupre Y, Stathaki E, Sailani MR, Baumer A et al. Methylation profiling in individuals with uniparental disomy identifies novel differentially methylated regions on chromosome 15. Genome Res. 2010;20:1271-8.

16. Aziz A, Baxter EJ, Edwards C, Cheong CY, Ito M, Bench A et al. Cooperativity of imprinted genes inactivated by acquired chromosome 20q deletions. J Clin Invest. 2013;123:2169-82.
17. Turan S, Bastepe M. The *GNAS* complex locus and human diseases associated with loss-of-function mutations or epimutations within this imprinted gene. Horm Res Paediatr. 2013;80:229-41.
18. Girardot M, Cavaille J, Feil R. Small regulatory RNAs controlled by genomic imprinting and their contribution to human disease. Epigenetics. 2012;7:1341-8.
19. Zehavi L, Avraham R, Barzilai A, Bar-Ilan D, Navon R, Sidi Y et al. Silencing of a large microRNA cluster on human chromosome 14q32 in melanoma: biological effects of mir-376a and mir-376c on insulin growth factor 1 receptor. Mol Cancer. 2012;11:44.
20. Kumar A, Nayak S, Pathak P, Purkait S, Malgulawar PB, Sharma MC et al. Identification of miR-379/miR-656 (C14MC) cluster downregulation and associated epigenetic and transcription regulatory mechanism in oligodendrogliomas. J Neurooncol. 2018;139:23-31.
21. Greife A, Knievel J, Ribarska T, Niegisch G, Schulz WA. Concomitant downregulation of the imprinted genes *DLK1* and *MEG3* at 14q32.2 by epigenetic mechanisms in urothelial carcinoma. Clin Epigenetics. 2014;6:29.
22. Mong EF, Yang Y, Akat KM, Canfield J, VanWye J, Lockhart J et al. Chromosome 19 microRNA cluster enhances cell reprogramming by inhibiting epithelial-to-mesenchymal transition. Sci Rep. 2020;10:3029.
23. Vaira V, Elli F, Forno I, Guarnieri V, Verdelli C, Ferrero S et al. The microRNA cluster C19MC is deregulated in parathyroid tumours. J Mol Endocrinol. 2012;49:115-24.
24. Dey BK, Pfeifer K, Dutta A. The *H19* long noncoding RNA gives rise to microRNAs miR-675-3p and miR-675-5p to promote skeletal muscle differentiation and regeneration. Genes Dev. 2014;28:491-501.
25. Morcos L, Ge B, Koka V, Lam KC, Pokholok DK, Gunderson KL et al. Genome-wide assessment of imprinted expression in human cells. Genome Biol. 2011;12:R25.
26. Pfeifer K. Mechanisms of genomic imprinting. Am J Hum Genet. 2000;67:777-87.
27. Wawrzik M, Spiess AN, Herrmann R, Buiting K, Horsthemke B. Expression of *SNURF-SNRPN* upstream transcripts and epigenetic regulatory genes during human spermatogenesis. Eur J Hum Genet. 2009;17:1463-70.
28. Yuen RK, Jiang R, Penaherrera MS, McFadden DE, Robinson WP. Genome-wide mapping of imprinted differentially methylated regions by DNA methylation profiling of human placentas from triploidies. Epigenetics Chromatin. 2011;4:10.
29. Kanber D, Berulava T, Ammerpohl O, Mitter D, Richter J, Siebert R et al. The human retinoblastoma gene is imprinted. PLoS Genet. 2009;5:e1000790.
30. Frost JM, Moore GE. The importance of imprinting in the human placenta. PLoS Genet. 2010;6:e1001015.
31. Zwart R, Sleutels F, Wutz A, Schinkel AH, Barlow DP. Bidirectional action of the Igf2r imprint control element on upstream and downstream imprinted genes. Genes Dev. 2001;15:2361-6.
32. Hassan M, Butler MG. Prader-Willi syndrome and atypical submicroscopic 15q11-q13 deletions with or without imprinting defects. Eur J Med Genet. 2016;59:584-9.
33. Jacobs DI, Mao Y, Fu A, Kelly WK, Zhu Y. Dysregulated methylation at imprinted genes in prostate tumor tissue detected by methylation microarray. BMC Urol. 2013;13:37.
34. Li SS, Yu SL, Singh S. Epigenetic states and expression of imprinted genes in human embryonic stem cells. World J Stem Cells. 2010;2:97-102.
35. Silver MJ, Kessler NJ, Hennig BJ, Dominguez-Salas P, Laritsky E, Baker MS et al. Independent genomewide screens identify the tumor suppressor *VTRNA2-1* as a human epiallele responsive to periconceptional environment. Genome Biol. 2015;16:118.
36. Haruta M, Arai Y, Sugawara W, Watanabe N, Honda S, Ohshima J et al. Duplication of paternal *IGF2* or loss of maternal *IGF2 i*mprinting occurs in half of Wilms tumors with various structural *WT1* abnormalities. Genes Chromosomes Cancer. 2008;47:712-27.
37. GeneCards®: Human Gene Database. Available from: https://www.genecards.org/. Accessed: 18 November 2021
38. Skaar DA, Li Y, Bernal AJ, Hoyo C, Murphy SK, Jirtle RL. The human imprintome: regulatory mechanisms, methods of ascertainment, and roles in disease susceptibility. ILAR J. 2012;53:341-58.
