# Supplementary material for: Increased copy number of imprinted genes in the chromosomal region 20q11-q13.32 is associated with resistance to antitumor agents in cancer cell lines
Source: Clin Epigenetics. 2022 Dec 2;14:161. doi: 10.1186/s13148-022-01368-7 (PMC9716673; doi:10.1186/s13148-022-01368-7)
Supplement: Supplementary file 3 — Additional file 3: Table S2. Chromosomal segment (bin) assignment of imprinted genes used for false discovery rate adjustment of the p values in the association analysis of copy number data with drug response. [file 13148_2022_1368_MOESM3_ESM.pdf]

Table S2. Chromosomal segment (bin) assignment of imprinted genes used for false discovery rate adjustment of the *p* values in the association analysis of copy number data with drug response

Gene	Gene name in copy number analysis	Gene Category	Chromosomal location	Segment
INPP5F	*INPP5F*	protein-coding	10q26.11	1
WT1	*WT1*	protein-coding	11p13	2
WT1AS	*WT1-AS*	ncRNA	11p13	2
CDKN1C	*CDKN1C*	protein-coding	11p15.4	3
OSBPL5	*OSBPL5*	protein-coding	11p15.4	3
PHLDA2	*PHLDA2*	protein-coding	11p15.4	3
SLC22A18	*SLC22A18*	protein-coding	11p15.4	3
SLC22A18AS	*SLC22A18AS*	ncRNA	11p15.4	3
ZNF215	*ZNF215*	protein-coding	11p15.4	3
KCNQ1	*KCNQ1*	protein-coding	11p15.4-p15.5	3
KCNQ1DN	*KCNQ1DN*	ncRNA	11p15.4-p15.5	3
H19	*H19*	ncRNA	11p15.5	3
INS-IGF2	*INS-IGF2*	Combined region	11p15.5	3
IGF2	*IGF2*	protein-coding	11p15.5	3
IGF2AS	*IGF2-AS*	ncRNA	11p15.5	3
INS	*INS*	protein-coding	11p15.5	3
KCNQ1OT1	*KCNQ1OT1*	ncRNA	11p15.5	3
MIR483	*MIR483*	ncRNA	11p15.5	3
MIR675	*MIR675*	ncRNA	11p15.5	3
TH	*TH*	protein-coding	11p15.5	3
ANO1	*ANO1*	protein-coding	11q13.3	4
ZC3H12C	*ZC3H12C*	protein-coding	11q22.3	5
NTM	*NTM*	protein-coding	11q25	6
RBP5	*RBP5*	protein-coding	12p13.31	7
WIF1	*WIF1*	protein-coding	12q14.3	8
DCN	*DCN*	protein-coding	12q21.33	9
HTR2A	*HTR2A*	protein-coding	13q14.2	10
RB1	*RB1*	protein-coding	13q14.2	10
DLK1	*DLK1*	protein-coding	14q32.2	11
MEG3	*MEG3*	ncRNA	14q32.2	11
MEG8	*MEG8*	ncRNA	14q32.2-q32.31	11
RTL1	*RTL1*	protein-coding	14q32.2-q32.31	11
DIO3	*DIO3*	protein-coding	14q32.31	11
MIR134	*MIR134*	ncRNA	14q32.31	11
MIR379	*MIR379*	ncRNA	14q32.31	11
MIR409	*MIR409*	ncRNA	14q32.31	11
MIR410	*MIR410*	ncRNA	14q32.31	11

MIR487B	*MIR487B*	ncRNA	14q32.31	11
MIR656	*MIR656*	ncRNA	14q32.31	11
PAR1	*PAR1*	ncRNA	15q11.2	12
PAR5	*PAR5*	ncRNA	15q11.2	12
IPW	*IPW*	ncRNA	15q11.2	12
MAGEL2	*MAGEL2*	protein-coding	15q11.2	12
MKRN3	*MKRN3*	protein-coding	15q11.2	12
NDN	*NDN*	protein-coding	15q11.2	12
NPAP1	*NPAP1*	protein-coding	15q11.2	12
PWRN1	*PWRN1*	ncRNA	15q11.2	12
SNORD107	*SNORD107*	ncRNA	15q11.2	12
SNORD108	*SNORD108*	ncRNA	15q11.2	12
UBE3A	*UBE3A*	protein-coding	15q11.2	12
SNORD109A	*SNORD109A*	ncRNA	15q11.2	12
SNORD109B	*SNORD109B*	ncRNA	15q11.2	12
SNORD115-1	*SNORD115-1*	ncRNA	15q11.2	12
SNORD115-10	*SNORD115-10*	ncRNA	15q11.2	12
SNORD115-11	*SNORD115-11*	ncRNA	15q11.2	12
SNORD115-12	*SNORD115-12*	ncRNA	15q11.2	12
SNORD115-13	*SNORD115-13*	ncRNA	15q11.2	12
SNORD115-14	*SNORD115-14*	ncRNA	15q11.2	12
SNORD115-15	*SNORD115-15*	ncRNA	15q11.2	12
SNORD115-16	*SNORD115-16*	ncRNA	15q11.2	12
SNORD115-17	*SNORD115-17*	ncRNA	15q11.2	12
SNORD115-18	*SNORD115-18*	ncRNA	15q11.2	12
SNORD115-19	*SNORD115-19*	ncRNA	15q11.2	12
SNORD115-2	*SNORD115-2*	ncRNA	15q11.2	12
SNORD115-20	*SNORD115-20*	ncRNA	15q11.2	12
SNORD115-21	*SNORD115-21*	ncRNA	15q11.2	12
SNORD115-22	*SNORD115-22*	ncRNA	15q11.2	12
SNORD115-23	*SNORD115-23*	ncRNA	15q11.2	12
SNORD115-24	*SNORD115-24*	ncRNA	15q11.2	12
SNORD115-25	*SNORD115-25*	ncRNA	15q11.2	12
SNORD115-26	*SNORD115-26*	ncRNA	15q11.2	12
SNORD115-27	*SNORD115-27*	ncRNA	15q11.2	12
SNORD115-28	*SNORD115-28*	ncRNA	15q11.2	12
SNORD115-29	*SNORD115-29*	ncRNA	15q11.2	12
SNORD115-3	*SNORD115-3*	ncRNA	15q11.2	12
SNORD115-30	*SNORD115-30*	ncRNA	15q11.2	12
SNORD115-31	*SNORD115-31*	ncRNA	15q11.2	12
SNORD115-32	*SNORD115-32*	ncRNA	15q11.2	12
SNORD115-33	*SNORD115-33*	ncRNA	15q11.2	12
SNORD115-34	*SNORD115-34*	ncRNA	15q11.2	12
SNORD115-35	*SNORD115-35*	ncRNA	15q11.2	12
SNORD115-36	*SNORD115-36*	ncRNA	15q11.2	12

SNORD115-37	*SNORD115-37*	ncRNA	15q11.2	12
SNORD115-38	*SNORD115-38*	ncRNA	15q11.2	12
SNORD115-39	*SNORD115-39*	ncRNA	15q11.2	12
SNORD115-4	*SNORD115-4*	ncRNA	15q11.2	12
SNORD115-40	*SNORD115-40*	ncRNA	15q11.2	12
SNORD115-41	*SNORD115-41*	ncRNA	15q11.2	12
SNORD115-42	*SNORD115-42*	ncRNA	15q11.2	12
SNORD115-43	*SNORD115-43*	ncRNA	15q11.2	12
SNORD115-44	*SNORD115-44*	ncRNA	15q11.2	12
SNORD115-45	*SNORD115-45*	ncRNA	15q11.2	12
SNORD115-47	*SNORD115-47*	ncRNA	15q11.2	12
SNORD115-48	*SNORD115-48*	ncRNA	15q11.2	12
SNORD115-5	*SNORD115-5*	ncRNA	15q11.2	12
SNORD115-6	*SNORD115-6*	ncRNA	15q11.2	12
SNORD115-7	*SNORD115-7*	ncRNA	15q11.2	12
SNORD115-8	*SNORD115-8*	ncRNA	15q11.2	12
SNORD115-9	*SNORD115-9*	ncRNA	15q11.2	12
SNORD116-1	*SNORD116-1*	ncRNA	15q11.2	12
SNORD116-10	*SNORD116-10*	ncRNA	15q11.2	12
SNORD116-11	*SNORD116-11*	ncRNA	15q11.2	12
SNORD116-12	*SNORD116-12*	ncRNA	15q11.2	12
SNORD116-13	*SNORD116-13*	ncRNA	15q11.2	12
SNORD116-14	*SNORD116-14*	ncRNA	15q11.2	12
SNORD116-15	*SNORD116-15*	ncRNA	15q11.2	12
SNORD116-16	*SNORD116-16*	ncRNA	15q11.2	12
SNORD116-17	*SNORD116-17*	ncRNA	15q11.2	12
SNORD116-18	*SNORD116-18*	ncRNA	15q11.2	12
SNORD116-19	*SNORD116-19*	ncRNA	15q11.2	12
SNORD116-2	*SNORD116-2*	ncRNA	15q11.2	12
SNORD116-20	*SNORD116-20*	ncRNA	15q11.2	12
SNORD116-21	*SNORD116-21*	ncRNA	15q11.2	12
SNORD116-22	*SNORD116-22*	ncRNA	15q11.2	12
SNORD116-23	*SNORD116-23*	ncRNA	15q11.2	12
SNORD116-24	*SNORD116-24*	ncRNA	15q11.2	12
SNORD116-25	*SNORD116-25*	ncRNA	15q11.2	12
SNORD116-26	*SNORD116-26*	ncRNA	15q11.2	12
SNORD116-27	*SNORD116-27*	ncRNA	15q11.2	12
SNORD116-28	*SNORD116-28*	ncRNA	15q11.2	12
SNORD116-29	*SNORD116-29*	ncRNA	15q11.2	12
SNORD116-3	*SNORD116-3*	ncRNA	15q11.2	12
SNORD116-4	*SNORD116-4*	ncRNA	15q11.2	12
SNORD116-5	*SNORD116-5*	ncRNA	15q11.2	12
SNORD116-6	*SNORD116-6*	ncRNA	15q11.2	12
SNORD116-7	*SNORD116-7*	ncRNA	15q11.2	12
SNORD116-8	*SNORD116-8*	ncRNA	15q11.2	12

SNORD116-9	*SNORD116-9*	ncRNA	15q11.2	12
SNORD64	*SNORD64*	ncRNA	15q11.2	12
SNRPN	*SNRPN*	protein-coding	15q11.2	12
SNURF	*SNURF*	protein-coding	15q11.2	12
GABRA5	*GABRA5*	protein-coding	15q12	12
GABRB3	*GABRB3*	protein-coding	15q12	12
ATP10A	*ATP10A*	protein-coding	15q12	12
GABRG3	*GABRG3*	protein-coding	15q12	12
MIR184	*MIR184*	ncRNA	15q25.1	13
RASGRF1	*RASGRF1*	protein-coding	15q25.1	13
NAA60	*NAA60*	protein-coding	16p13.3	14
ZNF597	*ZNF597*	protein-coding	16p13.3	14
TCEB3C	*TCEB3C*	protein-coding	18q21.1	15
DNMT1	*DNMT1*	protein-coding	19p13.2	16
MIR517A	*MIR517A*	ncRNA	19q13.42	17
MIR371A	*MIR371A*	ncRNA	19q13.42	17
NLRP2	*NLRP2*	protein-coding	19q13.42	17
ZNF331	*ZNF331*	protein-coding	19q13.42	17
MIMT1	*MIMT1*	ncRNA	19q13.43	17
PEG3	*PEG3*	protein-coding	19q13.43	17
USP29	*USP29*	protein-coding	19q13.43	17
ZIM2	*ZIM2*	protein-coding	19q13.43	17
ZIM3	*ZIM3*	protein-coding	19q13.43	17
DIRAS3	*DIRAS3*	protein-coding	1p31.3	18
RNU5D-1	*RNU5D-1*	ncRNA	1p34.1	19
TP73	*TP73*	protein-coding	1p36.32	20
HM13	*HM13*	protein-coding	20q11.21	21
PSIMCT-1	*PSIMCT-1*	pseudogene	20q11.21	21
BLCAP	*BLCAP*	protein-coding	20q11.23	21
NNAT	*NNAT*	protein-coding	20q11.23	21
GDAP1L1	*GDAP1L1*	protein-coding	20q12	21
SGK2	*SGK2*	protein-coding	20q13.12	21
L3MBTL	*L3MBTL1*	protein-coding	20q13.12	21
MIR298	*MIR298*	ncRNA	20q13.32	21
GNAS	*GNAS*	protein-coding	20q13.32	21
GNASAS	*GNAS-AS1*	ncRNA	20q13.32	21
MIR296	*MIR296*	ncRNA	20q13.32	21
DGCR6	*DGCR6*	protein-coding	22q11.21	22
LRRTM1	*LRRTM1*	protein-coding	2p12	23
GPR1	*GPR1*	protein-coding	2q33.3	24
ZDBF2	*ZDBF2*	protein-coding	2q33.3	24
NAP1L5	*NAP1L5*	protein-coding	4q22.1	25
VTRNA2-1	*VTRNA2-1*	nc RNA	5q31.1	26
FAM50B	*FAM50B*	protein-coding	6p25.2	27
LIN28B	*LIN28B*	protein-coding	6q16.3-q21	28

AIM1	*AIM1*	protein-coding	6q21	28
HYMAI	*HYMAI*	ncRNA	6q24.2	29
PHACTR2	*PHACTR2*	protein-coding	6q24.2	29
PLAGL1	*PLAGL1*	protein-coding	6q24.2	29
AIRN	*AIRN*	ncRNA	6q25	29
SLC22A2	*SLC22A2*	protein-coding	6q25.3	29
SLC22A3	*SLC22A3*	protein-coding	6q25.3	29
IGF2R	*IGF2R*	protein-coding	6q25.3	29
GRB10	*GRB10*	protein-coding	7p12.1	30
DDC	*DDC*	protein-coding	7p12.2-p12.1	30
MAGI2	*MAGI2*	protein-coding	7q21.11	31
PEG10	*PEG10*	protein-coding	7q21.3	31
CALCR	*CALCR*	protein-coding	7q21.3	31
PPP1R9A	*PPP1R9A*	protein-coding	7q21.3	31
SGCE	*SGCE*	protein-coding	7q21.3	31
TFPI2	*TFPI2*	protein-coding	7q21.3	31
DLX5	*DLX5*	protein-coding	7q22	31
MESTIT1	*MESTIT1*	ncRNA	7q32.2	32
CPA4	*CPA4*	protein-coding	7q32.2	32
MEST	*MEST*	protein-coding	7q32.2	32
KLF14	*KLF14*	protein-coding	7q32.2	32
DLGAP2	*DLGAP2*	protein-coding	8p23.3	33
ZFAT-AS1	*ZFAT-AS1*	ncRNA	8q24.22	34
ZFAT	*ZFAT*	protein-coding	8q24.22	34
KCNK9	*KCNK9*	protein-coding	8q24.3	34
GLIS3	*GLIS3*	protein-coding	9p24.2	35
