# Supplementary material for: Increased copy number of imprinted genes in the chromosomal region 20q11-q13.32 is associated with resistance to antitumor agents in cancer cell lines
Source: Clin Epigenetics. 2022 Dec 2;14:161. doi: 10.1186/s13148-022-01368-7 (PMC9716673; doi:10.1186/s13148-022-01368-7)
Supplement: Supplementary file 5 — Additional file 5: Table S3. Median and range of the rounded copy number of imprinted genes in the 623 cell lines with available CCLE copy number data and GDSC drug response data. [file 13148_2022_1368_MOESM5_ESM.pdf]

Table S3. Median and range of the rounded copy number of imprinted genes in the 623 cell lines with available CCLE copy number data and GDSC drug response data

Gene	Median	Min	Max
SNORD116-1	2	1	5
SNORD116-2	2	1	5
SNORD116-3	2	1	5
SNORD116-4	2	1	5
SNORD116-5	2	1	5
SNORD116-6	2	1	5
SNORD116-7	2	1	5
SNORD116-8	2	1	5
SNORD116-9	2	1	5
SNORD116-10	2	1	5
SNORD116-11	2	1	5
SNORD116-12	2	1	5
SNORD116-13	2	1	5
SNORD116-14	2	1	5
SNORD116-15	2	1	5
SNORD116-16	2	1	5
SNORD116-17	2	1	5
SNORD116-18	2	1	5
SNORD116-20	2	1	5
SNORD116-21	2	1	5
SNORD116-22	2	1	5
SNORD116-23	2	1	5
SNORD116-24	2	1	5
SNORD116-25	2	1	5
SNORD115-2	2	1	5
SNORD116-26	2	1	5
SNORD116-27	2	1	5
SNORD115-3	2	1	5
SNORD115-4	2	0	5
SNORD115-5	2	0	5
SNORD115-6	2	0	5
SNORD115-7	2	0	5
SNORD115-8	2	0	5
SNORD115-9	2	0	5
SNORD115-10	2	0	5
SNORD115-11	2	0	5
SNORD115-12	2	0	5
SNORD115-13	2	0	5
SNORD115-14	2	0	5
SNORD115-15	2	0	5
SNORD115-16	2	0	5

SNORD115-17	2	0	5
SNORD115-18	2	0	5
SNORD115-19	2	0	5
SNORD115-20	2	0	5
SNORD115-21	2	0	5
SNORD115-22	2	1	5
SNORD115-23	2	1	5
SNORD115-25	2	1	5
SNORD115-26	2	1	5
SNORD115-29	2	0	5
SNORD115-30	2	1	5
SNORD115-31	2	1	5
SNORD115-32	2	1	5
SNORD115-33	2	1	5
SNORD115-34	2	1	5
SNORD115-35	2	1	5
SNORD115-36	2	0	5
SNORD115-37	2	1	5
SNORD115-38	2	1	5
SNORD115-39	2	1	5
SNORD115-40	2	1	5
SNORD115-41	2	1	5
SNORD115-42	2	1	5
SNORD115-43	2	0	5
SNORD115-44	2	1	5
MIR675	2	0	3
SNORD116-28	2	1	5
SNORD116-29	2	1	5
SNORD115-48	2	1	5
SNORD115-24	2	1	5
SNORD115-27	2	1	5
SNORD115-28	2	1	5
SNORD115-45	2	1	5
SNORD115-47	2	1	5
MIMT1	2	1	5
PSIMCT-1	2	1	14
MIR298	2	1	9
VTRNA2-1	2	1	3
AIRN	2	1	5
SGK2	2	1	7
CDKN1C	2	1	3
BLCAP	2	1	5
KCNQ1OT1	2	1	3
WIF1	2	1	11
ZIM3	2	1	5

OSBPL5	2	0	3
KLF14	2	1	5
PAR1	2	1	5
ZNF597	2	1	4
GNAS-AS1	2	1	9
TCEB3C	2	1	5
DCN	2	1	8
DDC	2	1	11
GLIS3	2	0	12
DIO3	2	1	5
DLX5	2	1	7
DNMT1	2	1	5
AIM1	2	1	14
INPP5F	2	1	4
PEG10	2	1	9
ZIM2	2	1	5
NPAP1	2	1	5
GABRA5	2	1	6
GABRB3	2	0	5
GABRG3	2	0	11
L3MBTL1	2	1	7
FAM50B	2	1	4
NAP1L5	2	1	4
RNU5D-1	2	1	5
GNAS	2	1	9
GPR1	2	1	4
H19	2	0	3
GRB10	2	0	11
MESTIT1	2	1	5
HTR2A	2	0	4
SNORD108	2	1	5
SNORD109A	2	0	5
SNORD109B	2	0	5
SNORD115-1	2	1	5
SNORD64	2	1	5
LRRTM1	2	1	4
IGF2	2	1	4
IGF2R	2	1	5
INS	2	1	4
IPW	2	1	5
KCNQ1	2	0	3
RTL1	2	1	5
LIN28B	2	0	4
MIR134	2	1	5
MIR184	2	1	4

MIR296	2	1	9
MEST	2	1	5
MIR371A	2	1	11
NDN	2	1	6
NNAT	2	1	5
MIR379	2	1	5
SLC22A18	2	1	3
SLC22A18AS	2	1	3
NTM	2	0	15
CPA4	2	1	5
IGF2-AS	2	1	4
KCNK9	2	0	7
WT1-AS	2	0	6
PEG3	2	1	5
PLAGL1	2	0	5
MAGEL2	2	1	6
ANO1	2	0	17
MEG3	2	1	5
ZNF331	2	1	11
KCNQ1DN	2	1	3
PPP1R9A	2	1	9
NLRP2	2	0	4
HYMAI	2	1	5
ATP10A	2	0	4
MIR409	2	1	5
MIR410	2	1	5
MIR517A	2	1	11
ZFAT	2	0	14
USP29	2	1	5
ZDBF2	2	1	4
RASGRF1	2	1	4
RB1	2	0	7
ZFAT-AS1	2	0	8
MIR483	2	1	4
SLC22A3	2	1	5
SLC22A2	2	0	5
SNRPN	2	1	8
MIR487B	2	1	5
TH	2	1	4
TP73	2	0	4
INS-IGF2	2	1	4
MIR656	2	1	5
PHLDA2	2	1	3
SNORD116-19	2	1	5
UBE3A	2	1	4

WT1	2	0	6
MKRN3	2	1	5
ZNF215	2	0	4
GDAP1L1	2	1	5
MEG8	2	1	5
PWRN1	2	0	5
TFPI2	2	1	9
CALCR	2	1	9
NAA60	2	1	4
PAR5	2	1	5
HM13	2	1	14
DGCR6	2	0	6
RBP5	2	1	8
ZC3H12C	2	0	10
DLK1	2	1	5
SGCE	2	1	9
SNURF	2	1	5
DIRAS3	2	1	5
SNORD107	2	1	5
DLGAP2	2	0	5
PHACTR2	2	1	5
MAGI2	2	0	8
