# Supplementary material for: Increased copy number of imprinted genes in the chromosomal region 20q11-q13.32 is associated with resistance to antitumor agents in cancer cell lines
Source: Clin Epigenetics. 2022 Dec 2;14:161. doi: 10.1186/s13148-022-01368-7 (PMC9716673; doi:10.1186/s13148-022-01368-7)
Supplement: Supplementary file 6 — Additional file 6: Fig. S3. Distribution of gene-averaged methylation beta values among 515 imprinted gene regions in 645 cell lines in the pancancer dataset. Shown is the combined distribution of all six gene regions and separate distribution plots for each imprinted gene region category. Horizontal axis represents gene region-averaged methylation beta values, whereas the vertical axis represents gene region counts. The 6 gene regions include TSS1500, TSS200, 5′ UTR (UTR5), 1st exon (EXON1), gene body (GENE BODY), and 3' UTR (UTR3). [file 13148_2022_1368_MOESM6_ESM.pdf]

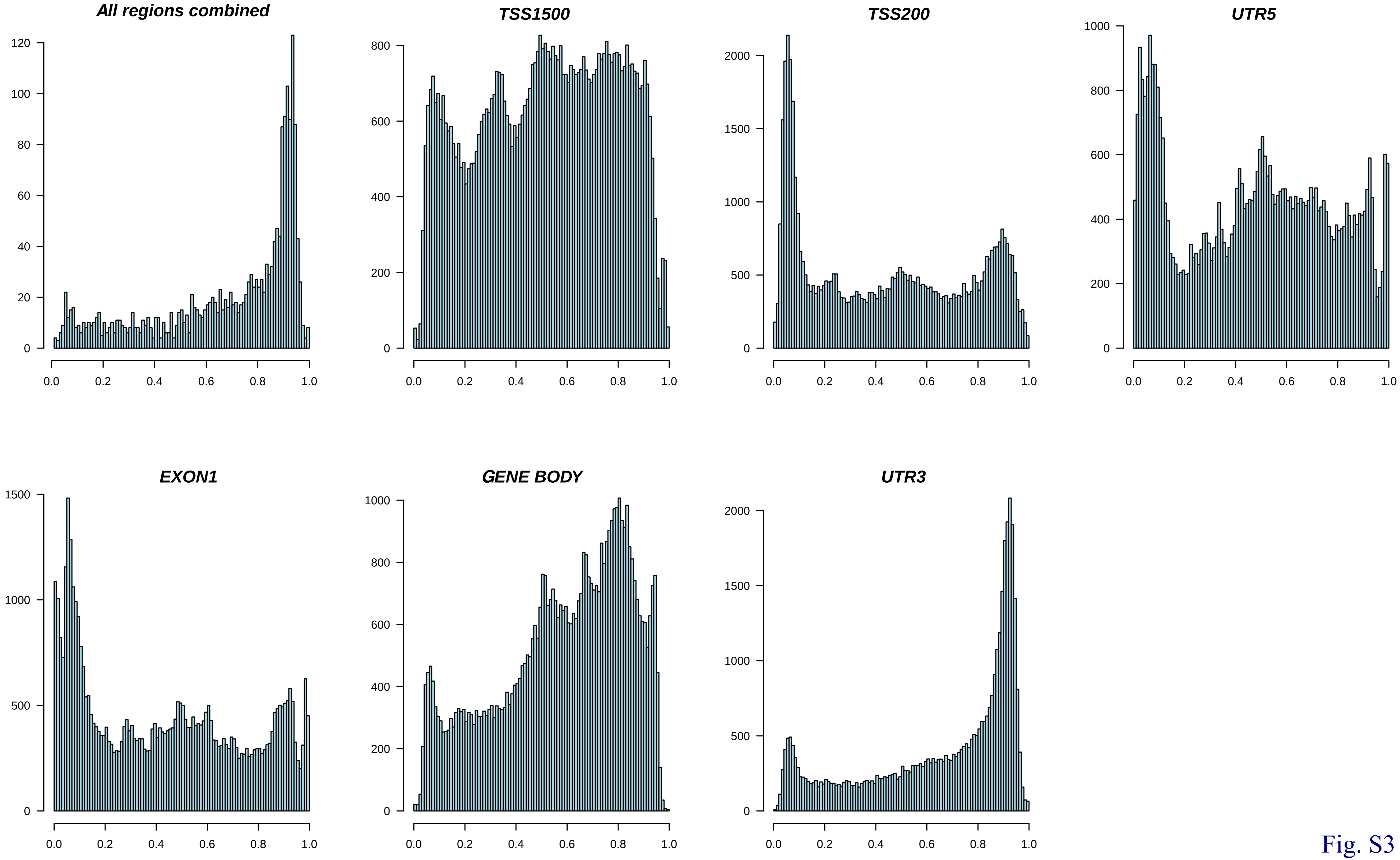
All regions combined
TSS1500
TSS200
UTR5
EXON1
GENE BODY
UTR3
0
20
40
60
80
100
120
200
400
600
800
500
1000
1500
2000
0.0
0.2
0.4
0.6
0.8
1.0

Fig. S3

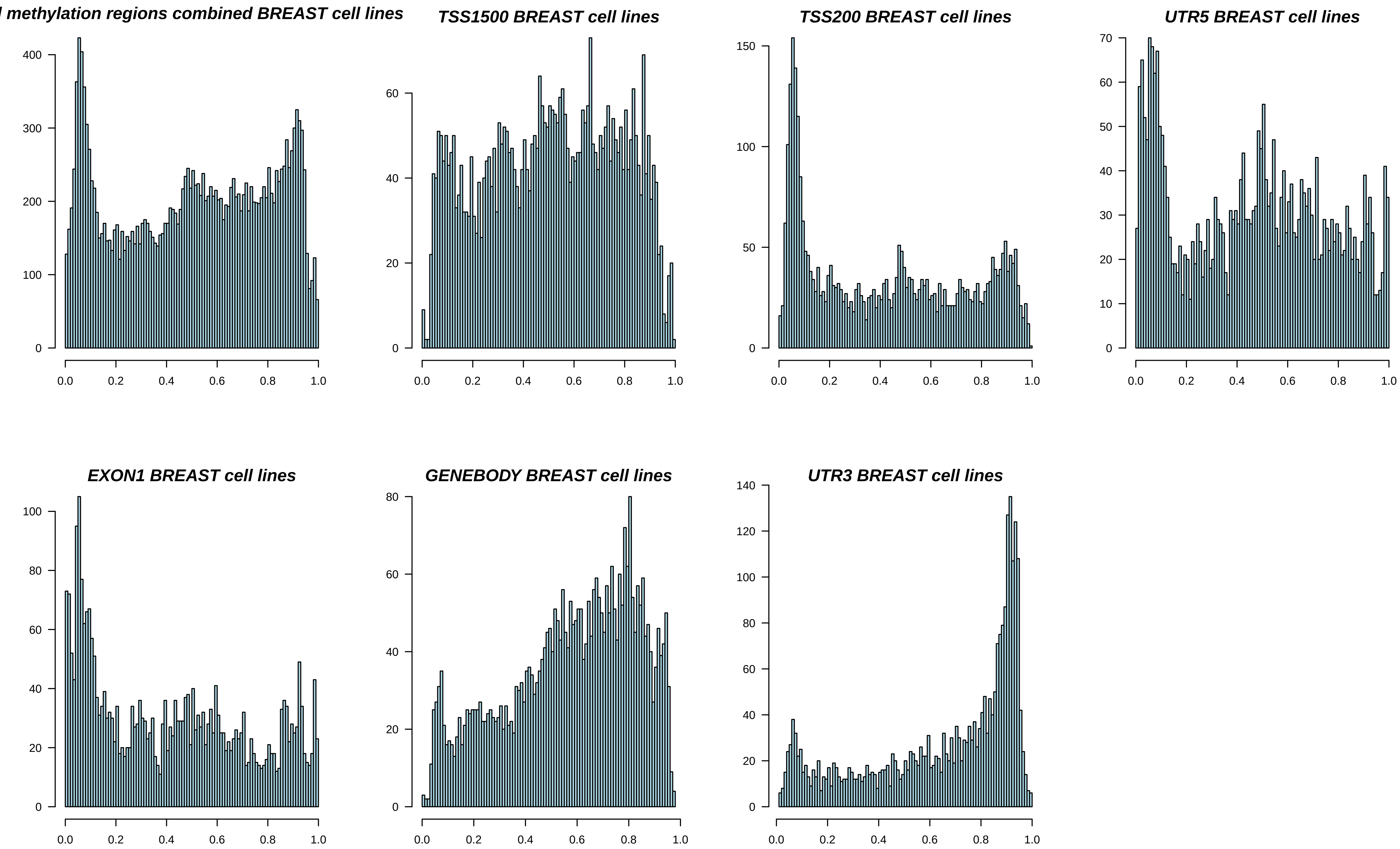
methylation regions combined BREAST cell lines
TSS1500 BREAST cell lines
TSS200 BREAST cell lines
UTR5 BREAST cell lines
EXON1 BREAST cell lines
GENEBODY BREAST cell lines
UTR3 BREAST cell lines

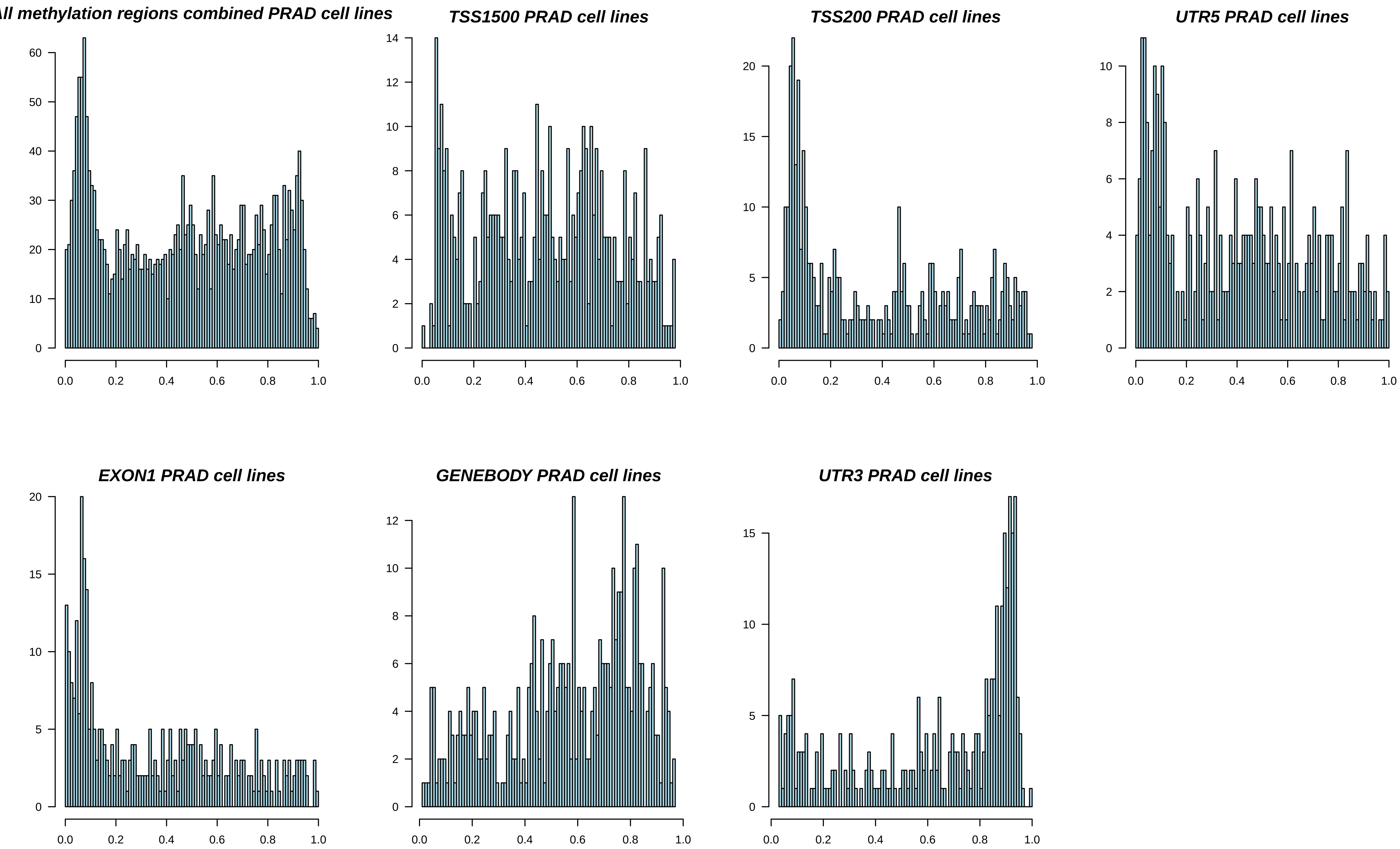
All methylation regions combined PRAD cell lines
TSS1500 PRAD cell lines
TSS200 PRAD cell lines
UTR5 PRAD cell lines
EXON1 PRAD cell lines
GENEBODY PRAD cell lines
UTR3 PRAD cell lines
0
10
20
30
40
50
60
0.0
0.2
0.4
0.6
0.8
1.0

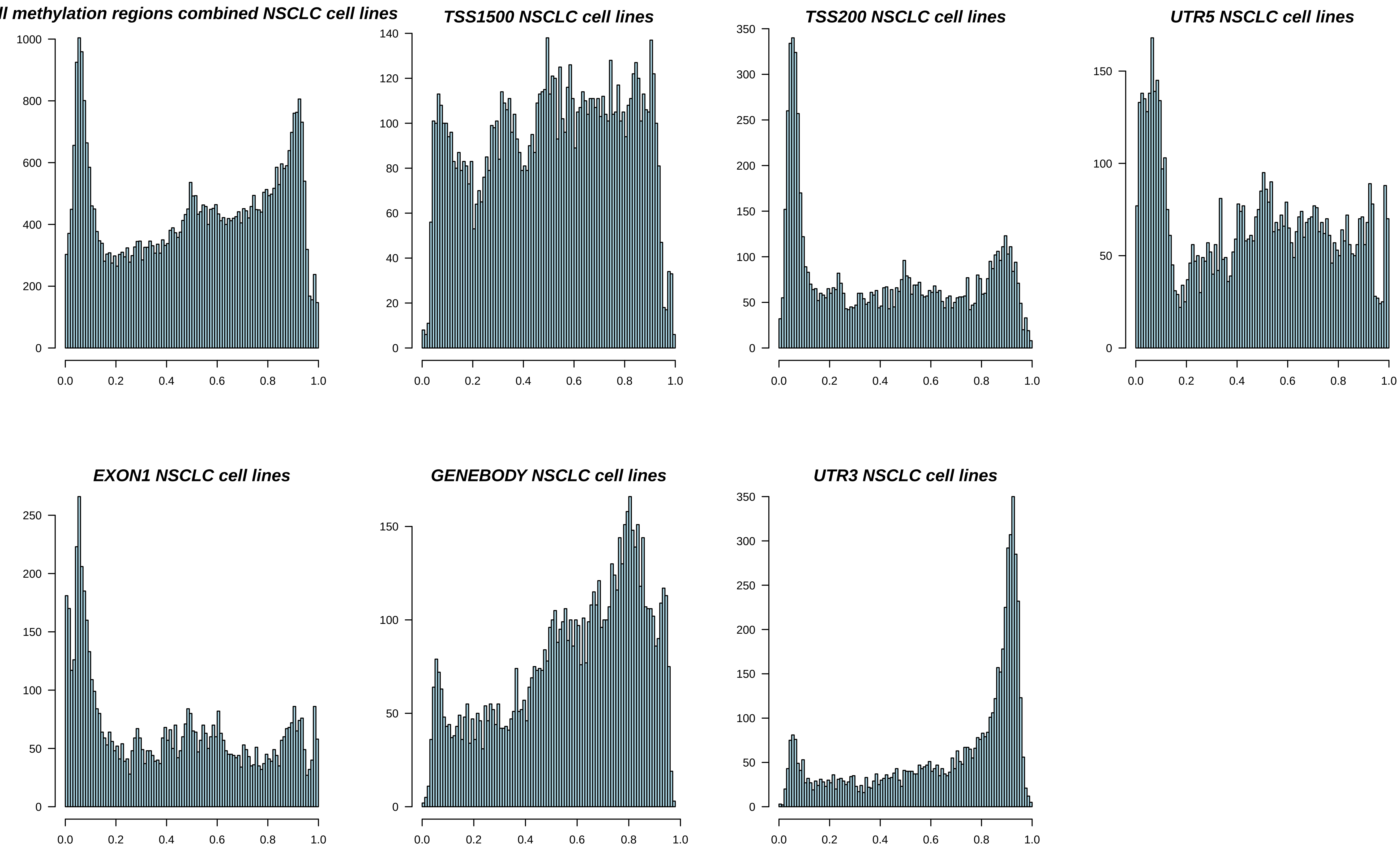
ll methylation regions combined NSCLC cell lines
TSS1500 NSCLC cell lines
TSS200 NSCLC cell lines
UTR5 NSCLC cell lines
EXON1 NSCLC cell lines
GENEBODY NSCLC cell lines
UTR3 NSCLC cell lines

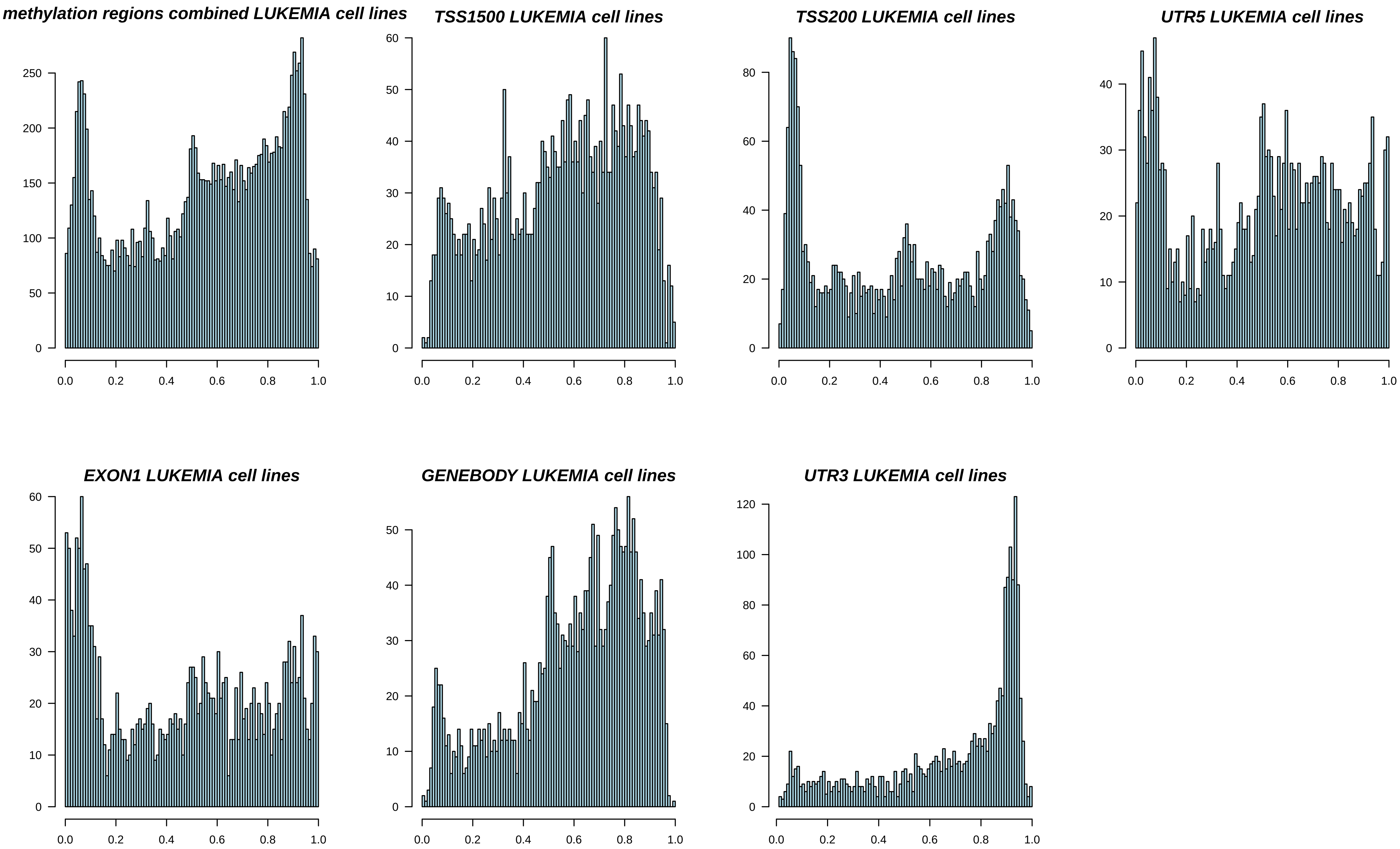
methylation regions combined LUKEMIA cell lines
0
50
100
150
200
250
0.0 0.2 0.4 0.6 0.8 1.0
TSS1500 LUKEMIA cell lines
0
10
20
30
40
50
60
0.0 0.2 0.4 0.6 0.8 1.0
TSS200 LUKEMIA cell lines
0
20
40
60
80
0.0 0.2 0.4 0.6 0.8 1.0
UTR5 LUKEMIA cell lines
0
10
20
30
40
0.0 0.2 0.4 0.6 0.8 1.0
EXON1 LUKEMIA cell lines
0
10
20
30
40
50
60
0.0 0.2 0.4 0.6 0.8 1.0
GENEBODY LUKEMIA cell lines
0
10
20
30
40
50
0.0 0.2 0.4 0.6 0.8 1.0
UTR3 LUKEMIA cell lines
0
20
40
60
80
100
120
0.0 0.2 0.4 0.6 0.8 1.0
