# Supplementary material for: Increased copy number of imprinted genes in the chromosomal region 20q11-q13.32 is associated with resistance to antitumor agents in cancer cell lines
Source: Clin Epigenetics. 2022 Dec 2;14:161. doi: 10.1186/s13148-022-01368-7 (PMC9716673; doi:10.1186/s13148-022-01368-7)
Supplement: Supplementary file 7 — Additional file 7: Table S4. Results of Spearman correlation analysis of continuous copy number values of the imprinted genes with log(IC50) satisfying pSegmFDR < 0.05 and Spearman |ρ| > 0.25. Sample size, number of cell lines with available data used in correlation analysis. Spearman ρ, Spearman correlation coefficient. The results are sorted by the absolute value of |ρ|. p0, p value prior to FDR adjustment. Segment, bin used for grouping imprinted genes according to their chromosomal location for FDR adjustment of the p values. Bin assignment of all imprinted genes is provided in Additional file 3: Table S2. max original segment p, maximal p value (prior to FDR adjustment) among all imprinted genes in a given chromosomal segment for a given agent. During the FDR adjustment each segment–agent pair was presented once by the maximal p value of all genes assigned to that segment in the correlation of their copy number with log(IC50) of that agent. pSegmFDR, p values after FDR adjustment using the maximal p values from each chromosomal segment. All correlations presented in the table also satisfied FDR-adjusted p < 0.05 if considering all genes independently, without grouping them into segments (pFDR < 0.05). Drug response data source, dataset (GDSC or CCLE) from which the drug response values were obtained. [file 13148_2022_1368_MOESM7_ESM.pdf]

Table S4. Results of Spearman correlation analysis of continuous copy number values of the imprinted genes with log(IC50) satisfying $p_{SegmFDR} < 0.05$ and Spearman $|\rho| > 0.25$

Gene	Agent	Sample size	Spearman ρ	p_O	Gene category	Chromosomal location	Segment	max original segment p	$p_{SegmFDR}$	Drug response data source
GNAS-AS1	Ispinesib mesylate	567	0.3393	9.54E-17	ncRNA	20q13.32	21	4.24E-12	3.74E-08	GDSC
MIR296	Ispinesib mesylate	567	0.3393	9.54E-17	ncRNA	20q13.32	21	4.24E-12	3.74E-08	GDSC
MIR298	Ispinesib mesylate	567	0.3393	9.54E-17	ncRNA	20q13.32	21	4.24E-12	3.74E-08	GDSC
GNAS	Ispinesib mesylate	567	0.3390	1.03E-16	protein-coding	20q13.32	21	4.24E-12	3.74E-08	GDSC
GNAS-AS1	T0901317	565	0.3333	4.03E-16	ncRNA	20q13.32	21	1.41E-09	9.08E-07	GDSC
MIR296	T0901317	565	0.3333	4.03E-16	ncRNA	20q13.32	21	1.41E-09	9.08E-07	GDSC
MIR298	T0901317	565	0.3333	4.03E-16	ncRNA	20q13.32	21	1.41E-09	9.08E-07	GDSC
GNAS	T0901317	565	0.3315	5.83E-16	protein-coding	20q13.32	21	1.41E-09	9.08E-07	GDSC
PSIMCT-1	Axitinib	506	0.3295	2.83E-14	pseudogene	20q11.21	21	1.04E-08	2.48E-06	GDSC
HM13	Axitinib	506	0.3295	2.83E-14	protein-coding	20q11.21	21	1.04E-08	2.48E-06	GDSC
HM13	T0901317	565	0.3239	2.86E-15	protein-coding	20q11.21	21	1.41E-09	9.08E-07	GDSC
PSIMCT-1	T0901317	565	0.3239	2.90E-15	pseudogene	20q11.21	21	1.41E-09	9.08E-07	GDSC
GNAS-AS1	XMD14-99	569	0.3172	9.10E-15	ncRNA	20q13.32	21	1.75E-08	3.18E-06	GDSC
MIR296	XMD14-99	569	0.3172	9.10E-15	ncRNA	20q13.32	21	1.75E-08	3.18E-06	GDSC
MIR298	XMD14-99	569	0.3172	9.10E-15	ncRNA	20q13.32	21	1.75E-08	3.18E-06	GDSC
HM13	Imatinib	227	0.3150	1.27E-06	protein-coding	20q11.21	21	0.00157	0.01754	GDSC
PSIMCT-1	Imatinib	227	0.3150	1.27E-06	pseudogene	20q11.21	21	0.00157	0.01754	GDSC
GNAS	XMD14-99	569	0.3150	1.43E-14	protein-coding	20q13.32	21	1.75E-08	3.18E-06	GDSC
GNAS-AS1	Cyclopamine	217	0.3149	2.22E-06	ncRNA	20q13.32	21	0.00110	0.01338	GDSC
MIR296	Cyclopamine	217	0.3149	2.22E-06	ncRNA	20q13.32	21	0.00110	0.01338	GDSC
MIR298	Cyclopamine	217	0.3149	2.22E-06	ncRNA	20q13.32	21	0.00110	0.01338	GDSC
GNAS-AS1	BX-912	569	0.3129	2.17E-14	ncRNA	20q13.32	21	2.21E-08	3.87E-06	GDSC
MIR296	BX-912	569	0.3129	2.17E-14	ncRNA	20q13.32	21	2.21E-08	3.87E-06	GDSC
MIR298	BX-912	569	0.3129	2.17E-14	ncRNA	20q13.32	21	2.21E-08	3.87E-06	GDSC
GNAS	XMD13-2	569	0.3120	2.62E-14	protein-coding	20q13.32	21	1.10E-08	2.48E-06	GDSC
GNAS	BX-912	569	0.3114	2.90E-14	protein-coding	20q13.32	21	2.21E-08	3.87E-06	GDSC
GNAS	Cyclopamine	217	0.3109	3.01E-06	protein-coding	20q13.32	21	0.00110	0.01338	GDSC
HM13	S-Trityl-L-cysteine	221	0.3108	2.46E-06	protein-coding	20q11.21	21	0.00029	0.00495	GDSC
PSIMCT-1	S-Trityl-L-cysteine	221	0.3108	2.46E-06	pseudogene	20q11.21	21	0.00029	0.00495	GDSC
GNAS	QL-XI-92	569	0.3106	3.41E-14	protein-coding	20q13.32	21	3.34E-09	1.57E-06	GDSC
GNAS-AS1	XMD13-2	569	0.3093	4.41E-14	ncRNA	20q13.32	21	1.10E-08	2.48E-06	GDSC
MIR296	XMD13-2	569	0.3093	4.41E-14	ncRNA	20q13.32	21	1.10E-08	2.48E-06	GDSC
MIR298	XMD13-2	569	0.3093	4.41E-14	ncRNA	20q13.32	21	1.10E-08	2.48E-06	GDSC
GNAS-AS1	QL-XI-92	569	0.3092	4.53E-14	ncRNA	20q13.32	21	3.34E-09	1.57E-06	GDSC
MIR296	QL-XI-92	569	0.3092	4.53E-14	ncRNA	20q13.32	21	3.34E-09	1.57E-06	GDSC
MIR298	QL-XI-92	569	0.3092	4.53E-14	ncRNA	20q13.32	21	3.34E-09	1.57E-06	GDSC
GNAS-AS1	GSK1070916	555	0.3072	1.37E-13	ncRNA	20q13.32	21	1.35E-08	2.76E-06	GDSC
MIR296	GSK1070916	555	0.3072	1.37E-13	ncRNA	20q13.32	21	1.35E-08	2.76E-06	GDSC
MIR298	GSK1070916	555	0.3072	1.37E-13	ncRNA	20q13.32	21	1.35E-08	2.76E-06	GDSC
GNAS	GSK1070916	555	0.3057	1.81E-13	protein-coding	20q13.32	21	1.35E-08	2.76E-06	GDSC
GNAS-AS1	UNC1215	553	0.3054	2.13E-13	ncRNA	20q13.32	21	3.40E-08	5.28E-06	GDSC
MIR296	UNC1215	553	0.3054	2.13E-13	ncRNA	20q13.32	21	3.40E-08	5.28E-06	GDSC
MIR298	UNC1215	553	0.3054	2.13E-13	ncRNA	20q13.32	21	3.40E-08	5.28E-06	GDSC
GNAS-AS1	GSK429286A	569	0.3052	9.81E-14	ncRNA	20q13.32	21	2.97E-09	1.50E-06	GDSC
MIR296	GSK429286A	569	0.3052	9.81E-14	ncRNA	20q13.32	21	2.97E-09	1.50E-06	GDSC
MIR298	GSK429286A	569	0.3052	9.81E-14	ncRNA	20q13.32	21	2.97E-09	1.50E-06	GDSC

BLCAP	Ispinesib mesylate	567	0.3044	1.28E-13	protein-coding	20q11.23	21	4.24E-12	3.74E-08	GDSC
NNAT	Ispinesib mesylate	567	0.3044	1.28E-13	protein-coding	20q11.23	21	4.24E-12	3.74E-08	GDSC
GNAS	UNC1215	553	0.3037	2.91E-13	protein-coding	20q13.32	21	3.40E-08	5.28E-06	GDSC
HM13	TL-2-105	569	0.3034	1.40E-13	protein-coding	20q11.21	21	1.11E-10	1.34E-07	GDSC
GNAS	GSK429286A	569	0.3028	1.59E-13	protein-coding	20q13.32	21	2.97E-09	1.50E-06	GDSC
GNAS	Masitinib	568	0.3023	1.81E-13	protein-coding	20q13.32	21	8.45E-09	2.31E-06	GDSC
PSIMCT-1	TL-2-105	569	0.3021	1.79E-13	pseudogene	20q11.21	21	1.11E-10	1.34E-07	GDSC
PSIMCT-1	UNC1215	553	0.3019	4.05E-13	pseudogene	20q11.21	21	3.40E-08	5.28E-06	GDSC
HM13	Ispinesib mesylate	567	0.3004	2.74E-13	protein-coding	20q11.21	21	4.24E-12	3.74E-08	GDSC
HM13	UNC1215	553	0.2994	6.43E-13	protein-coding	20q11.21	21	3.40E-08	5.28E-06	GDSC
GNAS-AS1	Masitinib	568	0.2993	3.21E-13	ncRNA	20q13.32	21	8.45E-09	2.31E-06	GDSC
MIR296	Masitinib	568	0.2993	3.21E-13	ncRNA	20q13.32	21	8.45E-09	2.31E-06	GDSC
MIR298	Masitinib	568	0.2993	3.21E-13	ncRNA	20q13.32	21	8.45E-09	2.31E-06	GDSC
PSIMCT-1	Ispinesib mesylate	567	0.2989	3.65E-13	pseudogene	20q11.21	21	4.24E-12	3.74E-08	GDSC
GNAS	TL-2-105	569	0.2988	3.37E-13	protein-coding	20q13.32	21	1.11E-10	1.34E-07	GDSC
GDAP1L1	Ispinesib mesylate	567	0.2983	4.06E-13	protein-coding	20q12	21	4.24E-12	3.74E-08	GDSC
GNAS-AS1	Veliparib	508	0.2965	9.01E-12	ncRNA	20q13.32	21	9.00E-07	6.51E-05	GDSC
MIR296	Veliparib	508	0.2965	9.01E-12	ncRNA	20q13.32	21	9.00E-07	6.51E-05	GDSC
MIR298	Veliparib	508	0.2965	9.01E-12	ncRNA	20q13.32	21	9.00E-07	6.51E-05	GDSC
GNAS-AS1	TL-2-105	569	0.2964	5.30E-13	ncRNA	20q13.32	21	1.11E-10	1.34E-07	GDSC
MIR296	TL-2-105	569	0.2964	5.30E-13	ncRNA	20q13.32	21	1.11E-10	1.34E-07	GDSC
MIR298	TL-2-105	569	0.2964	5.30E-13	ncRNA	20q13.32	21	1.11E-10	1.34E-07	GDSC
GNAS	Veliparib	508	0.2941	1.35E-11	protein-coding	20q13.32	21	9.00E-07	6.51E-05	GDSC
PSIMCT-1	Veliparib	508	0.2936	1.48E-11	pseudogene	20q11.21	21	9.00E-07	6.51E-05	GDSC
GNAS-AS1	Tivozanib	568	0.2933	9.92E-13	ncRNA	20q13.32	21	4.15E-09	1.66E-06	GDSC
MIR296	Tivozanib	568	0.2933	9.92E-13	ncRNA	20q13.32	21	4.15E-09	1.66E-06	GDSC
MIR298	Tivozanib	568	0.2933	9.92E-13	ncRNA	20q13.32	21	4.15E-09	1.66E-06	GDSC
GNAS	Tivozanib	568	0.2930	1.05E-12	protein-coding	20q13.32	21	4.15E-09	1.66E-06	GDSC
HM13	Salubrinal	218	0.2919	1.18E-05	protein-coding	20q11.21	21	0.00335	0.03152	GDSC
PSIMCT-1	Salubrinal	218	0.2919	1.18E-05	pseudogene	20q11.21	21	0.00335	0.03152	GDSC
GNAS-AS1	Y-39983	570	0.2916	1.24E-12	ncRNA	20q13.32	21	4.68E-09	1.73E-06	GDSC
MIR296	Y-39983	570	0.2916	1.24E-12	ncRNA	20q13.32	21	4.68E-09	1.73E-06	GDSC
MIR298	Y-39983	570	0.2916	1.24E-12	ncRNA	20q13.32	21	4.68E-09	1.73E-06	GDSC
GNAS	Y-39983	570	0.2914	1.27E-12	protein-coding	20q13.32	21	4.68E-09	1.73E-06	GDSC
PSIMCT-1	Quizartinib	568	0.2902	1.76E-12	pseudogene	20q11.21	21	1.51E-09	9.09E-07	GDSC
HM13	Quizartinib	568	0.2899	1.83E-12	protein-coding	20q11.21	21	1.51E-09	9.09E-07	GDSC
HM13	Veliparib	508	0.2898	2.77E-11	protein-coding	20q11.21	21	9.00E-07	6.51E-05	GDSC
GNAS-AS1	STF-62247	566	0.2895	2.17E-12	ncRNA	20q13.32	21	2.43E-08	4.18E-06	GDSC
MIR296	STF-62247	566	0.2895	2.17E-12	ncRNA	20q13.32	21	2.43E-08	4.18E-06	GDSC
MIR298	STF-62247	566	0.2895	2.17E-12	ncRNA	20q13.32	21	2.43E-08	4.18E-06	GDSC
HM13	Masitinib	568	0.2882	2.52E-12	protein-coding	20q11.21	21	8.45E-09	2.31E-06	GDSC
GNAS-AS1	VX-702	506	0.2881	3.95E-11	ncRNA	20q13.32	21	5.66E-06	0.00025	GDSC
MIR296	VX-702	506	0.2881	3.95E-11	ncRNA	20q13.32	21	5.66E-06	0.00025	GDSC
MIR298	VX-702	506	0.2881	3.95E-11	ncRNA	20q13.32	21	5.66E-06	0.00025	GDSC
BLCAP	T0901317	565	0.2876	3.20E-12	protein-coding	20q11.23	21	1.41E-09	9.08E-07	GDSC
NNAT	T0901317	565	0.2876	3.20E-12	protein-coding	20q11.23	21	1.41E-09	9.08E-07	GDSC
PSIMCT-1	Masitinib	568	0.2875	2.88E-12	pseudogene	20q11.21	21	8.45E-09	2.31E-06	GDSC
GNAS-AS1	TKI258	362	0.2874	2.60E-08	ncRNA	20q13.32	21	1.88E-05	0.00062	CCLE

MIR296	TKI258	362	0.2874	2.60E-08	ncRNA	20q13.32	21	1.88E-05	0.00062	CCLE
MIR298	TKI258	362	0.2874	2.60E-08	ncRNA	20q13.32	21	1.88E-05	0.00062	CCLE
PPP1R9A	Imatinib	227	0.2873	1.09E-05	protein-coding	7q21.3	31	0.00273	0.02710	GDSC
GNAS	STF-62247	566	0.2872	3.31E-12	protein-coding	20q13.32	21	2.43E-08	4.18E-06	GDSC
GNAS-AS1	Axitinib	506	0.2870	4.77E-11	ncRNA	20q13.32	21	1.04E-08	2.48E-06	GDSC
MIR296	Axitinib	506	0.2870	4.77E-11	ncRNA	20q13.32	21	1.04E-08	2.48E-06	GDSC
MIR298	Axitinib	506	0.2870	4.77E-11	ncRNA	20q13.32	21	1.04E-08	2.48E-06	GDSC
GNAS-AS1	XMD15-27	569	0.2858	3.69E-12	ncRNA	20q13.32	21	1.28E-07	1.44E-05	GDSC
MIR296	XMD15-27	569	0.2858	3.69E-12	ncRNA	20q13.32	21	1.28E-07	1.44E-05	GDSC
MIR298	XMD15-27	569	0.2858	3.69E-12	ncRNA	20q13.32	21	1.28E-07	1.44E-05	GDSC
GNAS	VX-702	506	0.2858	5.73E-11	protein-coding	20q13.32	21	5.66E-06	0.00025	GDSC
SGK2	Ispinesib mesylate	567	0.2857	4.13E-12	protein-coding	20q13.12	21	4.24E-12	3.74E-08	GDSC
PEG10	Imatinib	227	0.2857	1.23E-05	protein-coding	7q21.3	31	0.00273	0.02710	GDSC
SGCE	Imatinib	227	0.2857	1.23E-05	protein-coding	7q21.3	31	0.00273	0.02710	GDSC
GNAS	KIN001-260	570	0.2856	3.68E-12	protein-coding	20q13.32	21	2.13E-10	2.28E-07	GDSC
L3MBTL1	Ispinesib mesylate	567	0.2855	4.24E-12	protein-coding	20q13.12	21	4.24E-12	3.74E-08	GDSC
PSIMCT-1	Zibotentan	570	0.2851	4.01E-12	pseudogene	20q11.21	21	1.64E-07	1.78E-05	GDSC
GNAS-AS1	TPCA-1	570	0.2848	4.22E-12	ncRNA	20q13.32	21	1.16E-07	1.34E-05	GDSC
MIR296	TPCA-1	570	0.2848	4.22E-12	ncRNA	20q13.32	21	1.16E-07	1.34E-05	GDSC
MIR298	TPCA-1	570	0.2848	4.22E-12	ncRNA	20q13.32	21	1.16E-07	1.34E-05	GDSC
GNAS-AS1	SGC0946	552	0.2848	9.32E-12	ncRNA	20q13.32	21	9.71E-08	1.17E-05	GDSC
MIR296	SGC0946	552	0.2848	9.32E-12	ncRNA	20q13.32	21	9.71E-08	1.17E-05	GDSC
MIR298	SGC0946	552	0.2848	9.32E-12	ncRNA	20q13.32	21	9.71E-08	1.17E-05	GDSC
GNAS	TKI258	362	0.2847	3.54E-08	protein-coding	20q13.32	21	1.88E-05	0.00062	CCLE
GNAS	Axitinib	506	0.2843	7.35E-11	protein-coding	20q13.32	21	1.04E-08	2.48E-06	GDSC
GNAS-AS1	NPK76-II-72-1	570	0.2839	4.99E-12	ncRNA	20q13.32	21	1.11E-08	2.48E-06	GDSC
MIR296	NPK76-II-72-1	570	0.2839	4.99E-12	ncRNA	20q13.32	21	1.11E-08	2.48E-06	GDSC
MIR298	NPK76-II-72-1	570	0.2839	4.99E-12	ncRNA	20q13.32	21	1.11E-08	2.48E-06	GDSC
GNAS	XMD15-27	569	0.2838	5.32E-12	protein-coding	20q13.32	21	1.28E-07	1.44E-05	GDSC
HM13	Zibotentan	570	0.2833	5.58E-12	protein-coding	20q11.21	21	1.64E-07	1.78E-05	GDSC
GNAS-AS1	KIN001-260	570	0.2831	5.72E-12	ncRNA	20q13.32	21	2.13E-10	2.28E-07	GDSC
MIR296	KIN001-260	570	0.2831	5.72E-12	ncRNA	20q13.32	21	2.13E-10	2.28E-07	GDSC
MIR298	KIN001-260	570	0.2831	5.72E-12	ncRNA	20q13.32	21	2.13E-10	2.28E-07	GDSC
GNAS	SGC0946	552	0.2831	1.25E-11	protein-coding	20q13.32	21	9.71E-08	1.17E-05	GDSC
GNAS	TAK-715	569	0.2830	6.11E-12	protein-coding	20q13.32	21	8.10E-09	2.31E-06	GDSC
GNAS	NPK76-II-72-1	570	0.2827	6.14E-12	protein-coding	20q13.32	21	1.11E-08	2.48E-06	GDSC
GNAS	TPCA-1	570	0.2826	6.28E-12	protein-coding	20q13.32	21	1.16E-07	1.34E-05	GDSC
LRRTM1	GSK690693	567	0.2821	7.77E-12	protein-coding	2p12	23	7.77E-12	3.74E-08	GDSC
GDAP1L1	TL-2-105	569	0.2820	7.34E-12	protein-coding	20q12	21	1.11E-10	1.34E-07	GDSC
TFPI2	Imatinib	227	0.2817	1.64E-05	protein-coding	7q21.3	31	0.00273	0.02710	GDSC
GNAS-AS1	Fedratinib	569	0.2806	9.34E-12	ncRNA	20q13.32	21	6.20E-05	0.00156	GDSC
MIR296	Fedratinib	569	0.2806	9.34E-12	ncRNA	20q13.32	21	6.20E-05	0.00156	GDSC
MIR298	Fedratinib	569	0.2806	9.34E-12	ncRNA	20q13.32	21	6.20E-05	0.00156	GDSC
SGK2	TL-2-105	569	0.2804	9.66E-12	protein-coding	20q13.12	21	1.11E-10	1.34E-07	GDSC
L3MBTL1	TL-2-105	569	0.2802	9.98E-12	protein-coding	20q13.12	21	1.11E-10	1.34E-07	GDSC
GNAS-AS1	TAK-715	569	0.2801	1.01E-11	ncRNA	20q13.32	21	8.10E-09	2.31E-06	GDSC
MIR296	TAK-715	569	0.2801	1.01E-11	ncRNA	20q13.32	21	8.10E-09	2.31E-06	GDSC
MIR298	TAK-715	569	0.2801	1.01E-11	ncRNA	20q13.32	21	8.10E-09	2.31E-06	GDSC

DLK1	AZ628	220	0.2798	2.54E-05	protein-coding	14q32.2	11	2.55E-05	0.00078	GDSC
MEG3	AZ628	220	0.2798	2.54E-05	ncRNA	14q32.2	11	2.55E-05	0.00078	GDSC
MEG8	AZ628	220	0.2798	2.54E-05	ncRNA	14q32.2-q32.31	11	2.55E-05	0.00078	GDSC
MIR134	AZ628	220	0.2798	2.54E-05	ncRNA	14q32.31	11	2.55E-05	0.00078	GDSC
MIR379	AZ628	220	0.2798	2.54E-05	ncRNA	14q32.31	11	2.55E-05	0.00078	GDSC
MIR409	AZ628	220	0.2798	2.54E-05	ncRNA	14q32.31	11	2.55E-05	0.00078	GDSC
MIR410	AZ628	220	0.2798	2.54E-05	ncRNA	14q32.31	11	2.55E-05	0.00078	GDSC
MIR487B	AZ628	220	0.2798	2.54E-05	ncRNA	14q32.31	11	2.55E-05	0.00078	GDSC
MIR656	AZ628	220	0.2798	2.54E-05	ncRNA	14q32.31	11	2.55E-05	0.00078	GDSC
RTL1	AZ628	220	0.2798	2.54E-05	protein-coding	14q32.2-q32.31	11	2.55E-05	0.00078	GDSC
DIO3	AZ628	220	0.2798	2.55E-05	protein-coding	14q32.31	11	2.55E-05	0.00078	GDSC
GNAS	Fedratinib	569	0.2789	1.26E-11	protein-coding	20q13.32	21	6.20E-05	0.00156	GDSC
PSIMCT-1	SGC0946	552	0.2788	2.60E-11	pseudogene	20q11.21	21	9.71E-08	1.17E-05	GDSC
BLCAP	TAK-715	569	0.2783	1.40E-11	protein-coding	20q11.23	21	8.10E-09	2.31E-06	GDSC
NNAT	TAK-715	569	0.2783	1.40E-11	protein-coding	20q11.23	21	8.10E-09	2.31E-06	GDSC
HM13	KIN001-236	570	0.2777	1.49E-11	protein-coding	20q11.21	21	9.88E-08	1.17E-05	GDSC
HM13	SGC0946	552	0.2775	3.21E-11	protein-coding	20q11.21	21	9.71E-08	1.17E-05	GDSC
GNAS	CP466722	568	0.2775	1.68E-11	protein-coding	20q13.32	21	3.60E-09	1.57E-06	GDSC
GDAP1L1	CP466722	568	0.2774	1.70E-11	protein-coding	20q12	21	3.60E-09	1.57E-06	GDSC
GNAS	OSI-930	568	0.2774	1.72E-11	protein-coding	20q13.32	21	1.88E-07	1.97E-05	GDSC
GDAP1L1	T0901317	565	0.2773	1.97E-11	protein-coding	20q12	21	1.41E-09	9.08E-07	GDSC
PSIMCT-1	KIN001-236	570	0.2771	1.64E-11	pseudogene	20q11.21	21	9.88E-08	1.17E-05	GDSC
GNAS-AS1	CP466722	568	0.2764	2.02E-11	ncRNA	20q13.32	21	3.60E-09	1.57E-06	GDSC
MIR296	CP466722	568	0.2764	2.02E-11	ncRNA	20q13.32	21	3.60E-09	1.57E-06	GDSC
MIR298	CP466722	568	0.2764	2.02E-11	ncRNA	20q13.32	21	3.60E-09	1.57E-06	GDSC
PSIMCT-1	Tivozanib	568	0.2760	2.16E-11	pseudogene	20q11.21	21	4.15E-09	1.66E-06	GDSC
GDAP1L1	Imatinib	227	0.2758	2.50E-05	protein-coding	20q12	21	0.00157	0.01754	GDSC
HM13	STF-62247	566	0.2754	2.61E-11	protein-coding	20q11.21	21	2.43E-08	4.18E-06	GDSC
PSIMCT-1	STF-62247	566	0.2754	2.62E-11	pseudogene	20q11.21	21	2.43E-08	4.18E-06	GDSC
PSIMCT-1	GSK429286A	569	0.2754	2.32E-11	pseudogene	20q11.21	21	2.97E-09	1.50E-06	GDSC
HM13	Tivozanib	568	0.2753	2.45E-11	protein-coding	20q11.21	21	4.15E-09	1.66E-06	GDSC
HM13	GSK429286A	569	0.2746	2.66E-11	protein-coding	20q11.21	21	2.97E-09	1.50E-06	GDSC
DLX5	Imatinib	227	0.2746	2.73E-05	protein-coding	7q22	31	0.00273	0.02710	GDSC
GNAS-AS1	OSI-930	568	0.2744	2.89E-11	ncRNA	20q13.32	21	1.88E-07	1.97E-05	GDSC
MIR296	OSI-930	568	0.2744	2.89E-11	ncRNA	20q13.32	21	1.88E-07	1.97E-05	GDSC
MIR298	OSI-930	568	0.2744	2.89E-11	ncRNA	20q13.32	21	1.88E-07	1.97E-05	GDSC
PSIMCT-1	Linifanib	569	0.2743	2.81E-11	pseudogene	20q11.21	21	6.10E-06	0.00027	GDSC
GRB10	Imatinib	227	0.2740	2.83E-05	protein-coding	7p12.1	30	0.00010	0.00229	GDSC
HM13	Linifanib	569	0.2737	3.09E-11	protein-coding	20q11.21	21	6.10E-06	0.00027	GDSC
PSIMCT-1	Vorinostat	509	0.2733	3.61E-10	pseudogene	20q11.21	21	1.72E-05	0.00058	GDSC
GDAP1L1	S-Trityl-L-cysteine	221	0.2731	3.87E-05	protein-coding	20q12	21	0.00029	0.00495	GDSC
PSIMCT-1	VX-702	506	0.2729	4.32E-10	pseudogene	20q11.21	21	5.66E-06	0.00025	GDSC
GNAS-AS1	Genentech-Cpd-10	569	0.2727	3.66E-11	ncRNA	20q13.32	21	1.23E-06	8.10E-05	GDSC
MIR296	Genentech-Cpd-10	569	0.2727	3.66E-11	ncRNA	20q13.32	21	1.23E-06	8.10E-05	GDSC
MIR298	Genentech-Cpd-10	569	0.2727	3.66E-11	ncRNA	20q13.32	21	1.23E-06	8.10E-05	GDSC
GDAP1L1	KIN001-260	570	0.2727	3.53E-11	protein-coding	20q12	21	2.13E-10	2.28E-07	GDSC
GNAS	Genentech-Cpd-10	569	0.2721	4.11E-11	protein-coding	20q13.32	21	1.23E-06	8.10E-05	GDSC
SGK2	CP466722	568	0.2717	4.51E-11	protein-coding	20q13.12	21	3.60E-09	1.57E-06	GDSC

PSIMCT-1	Nilotinib	486	0.2716	1.15E-09	pseudogene	20q11.21	21	4.81E-06	0.00023	GDSC
GNAS-AS1	Nilotinib	486	0.2714	1.18E-09	ncRNA	20q13.32	21	4.81E-06	0.00023	GDSC
MIR296	Nilotinib	486	0.2714	1.18E-09	ncRNA	20q13.32	21	4.81E-06	0.00023	GDSC
MIR298	Nilotinib	486	0.2714	1.18E-09	ncRNA	20q13.32	21	4.81E-06	0.00023	GDSC
L3MBTL1	CP466722	568	0.2707	5.41E-11	protein-coding	20q13.12	21	3.60E-09	1.57E-06	GDSC
GDAP1L1	NPK76-II-72-1	570	0.2704	5.24E-11	protein-coding	20q12	21	1.11E-08	2.48E-06	GDSC
HM13	Nilotinib	486	0.2696	1.53E-09	protein-coding	20q11.21	21	4.81E-06	0.00023	GDSC
SGK2	KIN001-260	570	0.2693	6.33E-11	protein-coding	20q13.12	21	2.13E-10	2.28E-07	GDSC
GNAS	Nilotinib	486	0.2688	1.72E-09	protein-coding	20q13.32	21	4.81E-06	0.00023	GDSC
GNAS	PHA-793887	569	0.2687	7.19E-11	protein-coding	20q13.32	21	1.61E-06	0.00010	GDSC
ANO1	Crizotinib	226	0.2679	4.51E-05	protein-coding	11q13.3	4	4.51E-05	0.00119	GDSC
GNAS-AS1	PHA-793887	569	0.2678	8.42E-11	ncRNA	20q13.32	21	1.61E-06	0.00010	GDSC
MIR296	PHA-793887	569	0.2678	8.42E-11	ncRNA	20q13.32	21	1.61E-06	0.00010	GDSC
MIR298	PHA-793887	569	0.2678	8.42E-11	ncRNA	20q13.32	21	1.61E-06	0.00010	GDSC
BLCAP	UNC1215	553	0.2677	1.57E-10	protein-coding	20q11.23	21	3.40E-08	5.28E-06	GDSC
NNAT	UNC1215	553	0.2677	1.57E-10	protein-coding	20q11.23	21	3.40E-08	5.28E-06	GDSC
L3MBTL1	KIN001-260	570	0.2676	8.41E-11	protein-coding	20q13.12	21	2.13E-10	2.28E-07	GDSC
HM13	VX-702	506	0.2670	1.05E-09	protein-coding	20q11.21	21	5.66E-06	0.00025	GDSC
HM13	Vorinostat	509	0.2669	9.45E-10	protein-coding	20q11.21	21	1.72E-05	0.00058	GDSC
HM13	QL-XI-92	569	0.2667	1.01E-10	protein-coding	20q11.21	21	3.34E-09	1.57E-06	GDSC
BLCAP	TL-2-105	569	0.2662	1.11E-10	protein-coding	20q11.23	21	1.11E-10	1.34E-07	GDSC
NNAT	TL-2-105	569	0.2662	1.11E-10	protein-coding	20q11.23	21	1.11E-10	1.34E-07	GDSC
HM13	Amuvatinib	568	0.2660	1.18E-10	protein-coding	20q11.21	21	3.44E-07	3.16E-05	GDSC
HM13	Y-39983	570	0.2658	1.13E-10	protein-coding	20q11.21	21	4.68E-09	1.73E-06	GDSC
GDAP1L1	Cyclopamine	217	0.2657	7.40E-05	protein-coding	20q12	21	0.00110	0.01338	GDSC
BLCAP	Amuvatinib	568	0.2653	1.33E-10	protein-coding	20q11.23	21	3.44E-07	3.16E-05	GDSC
NNAT	Amuvatinib	568	0.2653	1.33E-10	protein-coding	20q11.23	21	3.44E-07	3.16E-05	GDSC
PSIMCT-1	Y-39983	570	0.2650	1.28E-10	pseudogene	20q11.21	21	4.68E-09	1.73E-06	GDSC
PSIMCT-1	TAK-715	569	0.2650	1.34E-10	pseudogene	20q11.21	21	8.10E-09	2.31E-06	GDSC
L3MBTL1	Axitinib	506	0.2650	1.41E-09	protein-coding	20q13.12	21	1.04E-08	2.48E-06	GDSC
HM13	Tubastatin-A	566	0.2648	1.55E-10	protein-coding	20q11.21	21	6.44E-07	5.21E-05	GDSC
GDAP1L1	Tivozanib	568	0.2646	1.50E-10	protein-coding	20q12	21	4.15E-09	1.66E-06	GDSC
HM13	OSI-930	568	0.2643	1.56E-10	protein-coding	20q11.21	21	1.88E-07	1.97E-05	GDSC
PSIMCT-1	QL-XI-92	569	0.2643	1.51E-10	pseudogene	20q11.21	21	3.34E-09	1.57E-06	GDSC
PSIMCT-1	KIN001-260	570	0.2643	1.46E-10	pseudogene	20q11.21	21	2.13E-10	2.28E-07	GDSC
PSIMCT-1	Amuvatinib	568	0.2642	1.59E-10	pseudogene	20q11.21	21	3.44E-07	3.16E-05	GDSC
GNAS-AS1	Lestaurtinib	508	0.2640	1.50E-09	ncRNA	20q13.32	21	0.00061	0.00884	GDSC
MIR296	Lestaurtinib	508	0.2640	1.50E-09	ncRNA	20q13.32	21	0.00061	0.00884	GDSC
MIR298	Lestaurtinib	508	0.2640	1.50E-09	ncRNA	20q13.32	21	0.00061	0.00884	GDSC
GNAS-AS1	BX795	508	0.2640	1.52E-09	ncRNA	20q13.32	21	4.04E-06	0.00020	GDSC
MIR296	BX795	508	0.2640	1.52E-09	ncRNA	20q13.32	21	4.04E-06	0.00020	GDSC
MIR298	BX795	508	0.2640	1.52E-09	ncRNA	20q13.32	21	4.04E-06	0.00020	GDSC
GNAS	Amuvatinib	568	0.2639	1.67E-10	protein-coding	20q13.32	21	3.44E-07	3.16E-05	GDSC
HM13	TAK-715	569	0.2638	1.64E-10	protein-coding	20q11.21	21	8.10E-09	2.31E-06	GDSC
PSIMCT-1	PD173074	507	0.2637	1.64E-09	pseudogene	20q11.21	21	5.57E-06	0.00025	GDSC
HM13	KIN001-260	570	0.2636	1.62E-10	protein-coding	20q11.21	21	2.13E-10	2.28E-07	GDSC
HM13	PD173074	507	0.2635	1.69E-09	protein-coding	20q11.21	21	5.57E-06	0.00025	GDSC
PSIMCT-1	OSI-930	568	0.2632	1.87E-10	pseudogene	20q11.21	21	1.88E-07	1.97E-05	GDSC

GDAP1L1	BX-912	569	0.2631	1.83E-10	protein-coding	20q12	21	2.21E-08	3.87E-06	GDSC
BLCAP	S-Trityl-L-cysteine	221	0.2630	7.55E-05	protein-coding	20q11.23	21	0.00029	0.00495	GDSC
NNAT	S-Trityl-L-cysteine	221	0.2630	7.55E-05	protein-coding	20q11.23	21	0.00029	0.00495	GDSC
PSIMCT-1	PFI-3	524	0.2630	9.72E-10	pseudogene	20q11.21	21	1.15E-05	0.00042	GDSC
PSIMCT-1	Tubastatin-A	566	0.2630	2.09E-10	pseudogene	20q11.21	21	6.44E-07	5.21E-05	GDSC
GNAS-AS1	AZD7762	508	0.2629	1.78E-09	ncRNA	20q13.32	21	7.84E-05	0.00184	GDSC
MIR296	AZD7762	508	0.2629	1.78E-09	ncRNA	20q13.32	21	7.84E-05	0.00184	GDSC
MIR298	AZD7762	508	0.2629	1.78E-09	ncRNA	20q13.32	21	7.84E-05	0.00184	GDSC
PSIMCT-1	XMD14-99	569	0.2628	1.91E-10	pseudogene	20q11.21	21	1.75E-08	3.18E-06	GDSC
GNAS	KIN001-270	570	0.2625	1.96E-10	protein-coding	20q13.32	21	8.54E-07	6.44E-05	GDSC
GNAS	Lestaurtinib	508	0.2623	1.93E-09	protein-coding	20q13.32	21	0.00061	0.00884	GDSC
PSIMCT-1	Daporinad	549	0.2622	4.38E-10	pseudogene	20q11.21	21	4.94E-07	4.28E-05	GDSC
BLCAP	KIN001-260	570	0.2620	2.13E-10	protein-coding	20q11.23	21	2.13E-10	2.28E-07	GDSC
NNAT	KIN001-260	570	0.2620	2.13E-10	protein-coding	20q11.23	21	2.13E-10	2.28E-07	GDSC
GNAS	BX795	508	0.2609	2.38E-09	protein-coding	20q13.32	21	4.04E-06	0.00020	GDSC
BLCAP	XMD13-2	569	0.2608	2.67E-10	protein-coding	20q11.23	21	1.10E-08	2.48E-06	GDSC
NNAT	XMD13-2	569	0.2608	2.67E-10	protein-coding	20q11.23	21	1.10E-08	2.48E-06	GDSC
HM13	XMD14-99	569	0.2607	2.71E-10	protein-coding	20q11.21	21	1.75E-08	3.18E-06	GDSC
HM13	Daporinad	549	0.2607	5.60E-10	protein-coding	20q11.21	21	4.94E-07	0.00004	GDSC
GDAP1L1	TKI258	362	0.2606	4.94E-07	protein-coding	20q12	21	1.88E-05	0.00062	CCLE
HM13	Panobinostat	358	0.2606	5.75E-07	protein-coding	20q11.21	21	0.00066	0.00930	CCLE
PSIMCT-1	Panobinostat	358	0.2606	5.75E-07	pseudogene	20q11.21	21	0.00066	0.00930	CCLE
GNAS-AS1	Amuvatinib	568	0.2605	2.92E-10	ncRNA	20q13.32	21	3.44E-07	3.16E-05	GDSC
MIR296	Amuvatinib	568	0.2605	2.92E-10	ncRNA	20q13.32	21	3.44E-07	3.16E-05	GDSC
MIR298	Amuvatinib	568	0.2605	2.92E-10	ncRNA	20q13.32	21	3.44E-07	3.16E-05	GDSC
BLCAP	QL-XII-61	270	0.2604	1.46E-05	protein-coding	20q11.23	21	0.00161	0.01785	GDSC
NNAT	QL-XII-61	270	0.2604	1.46E-05	protein-coding	20q11.23	21	0.00161	0.01785	GDSC
GNAS-AS1	Ruxolitinib	568	0.2603	2.98E-10	ncRNA	20q13.32	21	5.33E-07	4.54E-05	GDSC
MIR296	Ruxolitinib	568	0.2603	2.98E-10	ncRNA	20q13.32	21	5.33E-07	4.54E-05	GDSC
MIR298	Ruxolitinib	568	0.2603	2.98E-10	ncRNA	20q13.32	21	5.33E-07	4.54E-05	GDSC
CALCR	Imatinib	227	0.2603	7.25E-05	protein-coding	7q21.3	31	0.00273	0.02710	GDSC
HM13	PFI-3	524	0.2601	1.51E-09	protein-coding	20q11.21	21	1.15E-05	0.00042	GDSC
GNAS-AS1	KIN001-270	570	0.2601	2.89E-10	ncRNA	20q13.32	21	8.54E-07	6.44E-05	GDSC
MIR296	KIN001-270	570	0.2601	2.89E-10	ncRNA	20q13.32	21	8.54E-07	6.44E-05	GDSC
MIR298	KIN001-270	570	0.2601	2.89E-10	ncRNA	20q13.32	21	8.54E-07	6.44E-05	GDSC
HM13	XMD13-2	569	0.2597	3.18E-10	protein-coding	20q11.21	21	1.10E-08	2.48E-06	GDSC
GNAS	AZD7762	508	0.2593	3.01E-09	protein-coding	20q13.32	21	7.84E-05	0.00184	GDSC
VTRNA2-1	Panobinostat	358	0.2591	6.69E-07	nc RNA	5q31.1	26	6.69E-07	5.37E-05	CCLE
GDAP1L1	GSK429286A	569	0.2589	3.61E-10	protein-coding	20q12	21	2.97E-09	1.50E-06	GDSC
GDAP1L1	QL-XI-92	569	0.2589	3.62E-10	protein-coding	20q12	21	3.34E-09	1.57E-06	GDSC
BLCAP	BX-912	569	0.2588	3.66E-10	protein-coding	20q11.23	21	2.21E-08	3.87E-06	GDSC
NNAT	BX-912	569	0.2588	3.66E-10	protein-coding	20q11.23	21	2.21E-08	3.87E-06	GDSC
PSIMCT-1	VNLG-124	565	0.2587	4.30E-10	pseudogene	20q11.21	21	5.06E-05	0.00131	GDSC
PSIMCT-1	KIN001-270	570	0.2586	3.69E-10	pseudogene	20q11.21	21	8.54E-07	6.44E-05	GDSC
HM13	CP466722	568	0.2586	3.96E-10	protein-coding	20q11.21	21	3.60E-09	1.57E-06	GDSC
GNAS	Ruxolitinib	568	0.2583	4.14E-10	protein-coding	20q13.32	21	5.33E-07	4.54E-05	GDSC
GNAS-AS1	CX-5461	566	0.2583	4.45E-10	ncRNA	20q13.32	21	5.65E-08	7.90E-06	GDSC
MIR296	CX-5461	566	0.2583	4.45E-10	ncRNA	20q13.32	21	5.65E-08	7.90E-06	GDSC

MIR298	CX-5461	566	0.2583	4.45E-10	ncRNA	20q13.32	21	5.65E-08	7.90E-06	GDSC
HM13	VNLG-124	565	0.2581	4.73E-10	protein-coding	20q11.21	21	5.06E-05	0.00131	GDSC
SGK2	BX-912	569	0.2581	4.11E-10	protein-coding	20q13.12	21	2.21E-08	3.87E-06	GDSC
GNAS	CX-5461	566	0.2581	4.60E-10	protein-coding	20q13.32	21	5.65E-08	7.90E-06	GDSC
GDAP1L1	Axitinib	506	0.2579	3.91E-09	protein-coding	20q12	21	1.04E-08	2.48E-06	GDSC
L3MBTL1	Quizartinib	568	0.2579	4.40E-10	protein-coding	20q13.12	21	1.51E-09	9.09E-07	GDSC
GNAS	Selisistat	565	0.2579	4.92E-10	protein-coding	20q13.32	21	2.79E-05	0.00084	GDSC
GRB10	Daporinad	549	0.2576	8.97E-10	protein-coding	7p12.1	30	3.98E-09	1.66E-06	GDSC
GDAP1L1	XMD13-2	569	0.2575	4.55E-10	protein-coding	20q12	21	1.10E-08	2.48E-06	GDSC
PSIMCT-1	CP466722	568	0.2573	4.84E-10	pseudogene	20q11.21	21	3.60E-09	1.57E-06	GDSC
GNAS-AS1	FMK	514	0.2573	3.23E-09	ncRNA	20q13.32	21	6.90E-05	0.00169	GDSC
MIR296	FMK	514	0.2573	3.23E-09	ncRNA	20q13.32	21	6.90E-05	0.00169	GDSC
MIR298	FMK	514	0.2573	3.23E-09	ncRNA	20q13.32	21	6.90E-05	0.00169	GDSC
PSIMCT-1	GW-2580	569	0.2572	4.75E-10	pseudogene	20q11.21	21	2.02E-06	0.00012	GDSC
HM13	CX-5461	566	0.2572	5.30E-10	protein-coding	20q11.21	21	5.65E-08	7.90E-06	GDSC
L3MBTL1	BX-912	569	0.2571	4.86E-10	protein-coding	20q13.12	21	2.21E-08	3.87E-06	GDSC
HM13	Topotecan	362	0.2570	7.18E-07	protein-coding	20q11.21	21	0.00113	0.01359	CCLE
PSIMCT-1	Topotecan	362	0.2570	7.18E-07	pseudogene	20q11.21	21	0.00113	0.01359	CCLE
BLCAP	OSI-930	568	0.2570	5.10E-10	protein-coding	20q11.23	21	1.88E-07	1.97E-05	GDSC
NNAT	OSI-930	568	0.2570	5.10E-10	protein-coding	20q11.23	21	1.88E-07	1.97E-05	GDSC
PSIMCT-1	XMD13-2	569	0.2568	5.04E-10	pseudogene	20q11.21	21	1.10E-08	2.48E-06	GDSC
HM13	KIN001-270	570	0.2566	5.07E-10	protein-coding	20q11.21	21	8.54E-07	6.44E-05	GDSC
HM13	Cyclopamine	217	0.2560	0.000137	protein-coding	20q11.21	21	0.00110	0.01338	GDSC
PSIMCT-1	Cyclopamine	217	0.2560	0.000137	pseudogene	20q11.21	21	0.00110	0.01338	GDSC
BLCAP	NPK76-II-72-1	570	0.2557	5.86E-10	protein-coding	20q11.23	21	1.11E-08	2.48E-06	GDSC
NNAT	NPK76-II-72-1	570	0.2557	5.86E-10	protein-coding	20q11.23	21	1.11E-08	2.48E-06	GDSC
BLCAP	GSK1070916	555	0.2555	1.02E-09	protein-coding	20q11.23	21	1.35E-08	2.76E-06	GDSC
NNAT	GSK1070916	555	0.2555	1.02E-09	protein-coding	20q11.23	21	1.35E-08	2.76E-06	GDSC
GNAS	Quizartinib	568	0.2555	6.51E-10	protein-coding	20q13.32	21	1.51E-09	9.09E-07	GDSC
SGK2	Axitinib	506	0.2549	6.04E-09	protein-coding	20q13.12	21	1.04E-08	2.48E-06	GDSC
DDC	Imatinib	227	0.2548	0.000104	protein-coding	7p12.2-p12.1	30	0.00010	0.00229	GDSC
SGK2	Quizartinib	568	0.2548	7.26E-10	protein-coding	20q13.12	21	1.51E-09	9.09E-07	GDSC
GNAS-AS1	Selisistat	565	0.2546	8.27E-10	ncRNA	20q13.32	21	2.79E-05	0.00084	GDSC
MIR296	Selisistat	565	0.2546	8.27E-10	ncRNA	20q13.32	21	2.79E-05	0.00084	GDSC
MIR298	Selisistat	565	0.2546	8.27E-10	ncRNA	20q13.32	21	2.79E-05	0.00084	GDSC
PSIMCT-1	CX-5461	566	0.2545	8.07E-10	pseudogene	20q11.21	21	5.65E-08	7.90E-06	GDSC
L3MBTL1	T0901317	565	0.2545	8.41E-10	protein-coding	20q13.12	21	1.41E-09	9.08E-07	GDSC
GNAS-AS1	Quizartinib	568	0.2542	7.96E-10	ncRNA	20q13.32	21	1.51E-09	9.09E-07	GDSC
MIR296	Quizartinib	568	0.2542	7.96E-10	ncRNA	20q13.32	21	1.51E-09	9.09E-07	GDSC
MIR298	Quizartinib	568	0.2542	7.96E-10	ncRNA	20q13.32	21	1.51E-09	9.09E-07	GDSC
GNAS	FMK	514	0.2540	5.21E-09	protein-coding	20q13.32	21	6.90E-05	0.00169	GDSC
L3MBTL1	Tivozanib	568	0.2540	8.22E-10	protein-coding	20q13.12	21	4.15E-09	1.66E-06	GDSC
GNAS-AS1	BIX02189	570	0.2539	7.72E-10	ncRNA	20q13.32	21	9.37E-07	6.67E-05	GDSC
MIR296	BIX02189	570	0.2539	7.72E-10	ncRNA	20q13.32	21	9.37E-07	6.67E-05	GDSC
MIR298	BIX02189	570	0.2539	7.72E-10	ncRNA	20q13.32	21	9.37E-07	6.67E-05	GDSC
PSIMCT-1	Ruxolitinib	568	0.2538	8.42E-10	pseudogene	20q11.21	21	5.33E-07	4.54E-05	GDSC
L3MBTL1	S-Trityl-L-cysteine	221	0.2537	0.000138	protein-coding	20q13.12	21	0.00029	0.00495	GDSC
HM13	AZD8055	503	0.2533	8.38E-09	protein-coding	20q11.21	21	0.00049	0.00740	GDSC

ANO1	Seliciclib	216	0.2527	0.000174	protein-coding	11q13.3	4	0.00017	0.00335	GDSC
BLCAP	Salubrinal	218	0.2526	0.000164	protein-coding	20q11.23	21	0.00335	0.03152	GDSC
NNAT	Salubrinal	218	0.2526	0.000164	protein-coding	20q11.23	21	0.00335	0.03152	GDSC
GNAS	BIX02189	570	0.2526	9.59E-10	protein-coding	20q13.32	21	9.37E-07	6.67E-05	GDSC
PSIMCT-1	Lenalidomide	509	0.2522	8.01E-09	pseudogene	20q11.21	21	0.00014	0.00288	GDSC
GDAP1L1	STF-62247	566	0.2514	1.31E-09	protein-coding	20q12	21	2.43E-08	4.18E-06	GDSC
L3MBTL1	QL-XI-92	569	0.2514	1.20E-09	protein-coding	20q13.12	21	3.34E-09	1.57E-06	GDSC
HM13	GW-2580	569	0.2513	1.22E-09	protein-coding	20q11.21	21	2.02E-06	0.00012	GDSC
SGK2	T0901317	565	0.2511	1.41E-09	protein-coding	20q13.12	21	1.41E-09	9.08E-07	GDSC
BLCAP	Axitinib	506	0.2510	1.04E-08	protein-coding	20q11.23	21	1.04E-08	2.48E-06	GDSC
NNAT	Axitinib	506	0.2510	1.04E-08	protein-coding	20q11.23	21	1.04E-08	2.48E-06	GDSC
GDAP1L1	Masitinib	568	0.2510	1.32E-09	protein-coding	20q12	21	8.45E-09	2.31E-06	GDSC
GNAS-AS1	Temozolomide	552	0.2509	2.26E-09	ncRNA	20q13.32	21	2.01E-05	0.00065	GDSC
MIR296	Temozolomide	552	0.2509	2.26E-09	ncRNA	20q13.32	21	2.01E-05	0.00065	GDSC
MIR298	Temozolomide	552	0.2509	2.26E-09	ncRNA	20q13.32	21	2.01E-05	0.00065	GDSC
HM13	Ruxolitinib	568	0.2509	1.34E-09	protein-coding	20q11.21	21	5.33E-07	4.54E-05	GDSC
GDAP1L1	GSK1070916	555	0.2508	2.07E-09	protein-coding	20q12	21	1.35E-08	2.76E-06	GDSC
L3MBTL1	GSK429286A	569	0.2508	1.31E-09	protein-coding	20q13.12	21	2.97E-09	1.50E-06	GDSC
GDAP1L1	Y-39983	570	0.2503	1.37E-09	protein-coding	20q12	21	4.68E-09	1.73E-06	GDSC
PSIMCT-1	IOX2	569	0.2502	1.43E-09	pseudogene	20q11.21	21	1.82E-05	0.00061	GDSC
BLCAP	Quizartinib	568	0.2502	1.48E-09	protein-coding	20q11.23	21	1.51E-09	9.09E-07	GDSC
NNAT	Quizartinib	568	0.2502	1.48E-09	protein-coding	20q11.23	21	1.51E-09	9.09E-07	GDSC
GDAP1L1	Quizartinib	568	0.2501	1.51E-09	protein-coding	20q12	21	1.51E-09	9.09E-07	GDSC
DLGAP2	PF2341066	362	-0.2551	8.70E-07	protein-coding	8p23.3	33	8.70E-07	6.46E-05	CCLE
DNMT1	PI-103	564	-0.2557	7.21E-10	protein-coding	19p13.2	16	7.21E-10	4.96E-07	GDSC
TCEB3C	PI-103	564	-0.2590	4.29E-10	protein-coding	18q21.1	15	4.29E-10	3.18E-07	GDSC
DLGAP2	GSK429286A	569	-0.2593	3.41E-10	protein-coding	8p23.3	33	3.41E-10	2.74E-07	GDSC
TCEB3C	UNC0638	597	-0.2601	1.10E-10	protein-coding	18q21.1	15	1.10E-10	1.34E-07	GDSC
TCEB3C	GSK429286A	569	-0.2609	2.61E-10	protein-coding	18q21.1	15	2.61E-10	2.51E-07	GDSC
TCEB3C	Daporinad	549	-0.2643	3.14E-10	protein-coding	18q21.1	15	3.14E-10	2.74E-07	GDSC
TCEB3C	TL-2-105	569	-0.2671	9.50E-11	protein-coding	18q21.1	15	9.50E-11	1.34E-07	GDSC
DNMT1	THZ-2-102-1	563	-0.2675	1.12E-10	protein-coding	19p13.2	16	1.12E-10	1.34E-07	GDSC
TCEB3C	Quizartinib	568	-0.2681	8.34E-11	protein-coding	18q21.1	15	8.34E-11	1.34E-07	GDSC
TCEB3C	I-BET-762	566	-0.2697	6.86E-11	protein-coding	18q21.1	15	6.86E-11	1.34E-07	GDSC

Correlation results for the chromosomal region 20q11-q13.2 are highlighted in yellow.

Sample size, number of cell lines with available data used in correlation analysis
Spearman ρ, Spearman correlation coefficient**.** The results are sorted by the absolute value of |ρ|.
p_0, *p*- value prior to FDR adjustment

Segment, bin number used for grouping imprinted genes according to their chromosomal location for FDR adjustment of the *p*- values.
Bin assignment of all imprinted genes is provided in Table S2.

max original segment *p* , highest *p* -value (prior to FDR adjustment) among all imprinted genes in a given chromosomal segment for a given agent**.**
During the FDR adjustment each segment-agent pair was presented once by the maximal *p* -value of all genes assigned to that segment in the correlation of their copy number with log(IC50) of that agent.

$p_{SegmFDR}$, *p* -values after FDR adjustment using maximal *p* -values from each chromosomal segment.
All correlations presented in the table also satisfied FDR adjusted p <0.05 if considering all genes independently, without grouping them into segments ($p_{FDR} < 0.05$).

Drug response data source, dataset (GDSC or CCLE) from which the drug response values were obtained
