# Supplementary material for: Increased copy number of imprinted genes in the chromosomal region 20q11-q13.32 is associated with resistance to antitumor agents in cancer cell lines
Source: Clin Epigenetics. 2022 Dec 2;14:161. doi: 10.1186/s13148-022-01368-7 (PMC9716673; doi:10.1186/s13148-022-01368-7)
Supplement: Supplementary file 8 — Additional file 8: Table S5. Results of Spearman correlation analysis of expression of imprinted genes with log(IC50) satisfying pFDR < 0.05 and Spearman |ρ| > 0.3. Spearman ρ, Spearman correlation coefficient. The results are sorted by the absolute value of |ρ|. p0, p value prior to FDR adjustment. pFDR, p value after FDR adjustment. Sample size, number of cell lines with available data used in correlation analysis. Drug response data source, dataset (GDSC or CCLE) from which the drug response values were obtained. [file 13148_2022_1368_MOESM8_ESM.pdf]

**Table S5.** Results of Spearman correlation analysis of expression of imprinted genes with log(IC50) satisfying $p_{FDR} < 0.05$ and Spearman $|\rho| > 0.3$

| Gene | Agent | Spearman ρ | $p_0$ | $p_{FDR}$ | Sample size | Drug response data source |
|---|---|---|---|---|---|---|
| ***CPA4*** | Panobinostat | 0.4244 | 1.45E-17 | 6.70E-15 | 369 | CCLE |
| ***DNMT1*** | Zibotentan | -0.4214 | 7.74E-27 | 2.18E-22 | 591 | GDSC |
| ***DNMT1*** | XMD13-2 | -0.4043 | 1.31E-24 | 1.84E-20 | 590 | GDSC |
| ***DNMT1*** | Daporinad | -0.4036 | 1.06E-23 | 4.90E-20 | 569 | GDSC |
| ***PHLDA2*** | BX-912 | 0.4017 | 2.72E-24 | 2.55E-20 | 590 | GDSC |
| ***DNMT1*** | Tivozanib | -0.3991 | 6.28E-24 | 4.42E-20 | 589 | GDSC |
| ***DNMT1*** | Tubastatin A | -0.3974 | 1.22E-23 | 4.90E-20 | 587 | GDSC |
| ***DNMT1*** | KIN001-270 | -0.3965 | 1.10E-23 | 4.90E-20 | 591 | GDSC |
| ***DGCR6*** | Cyclopamine | 0.3960 | 1.03E-09 | 4.61E-08 | 221 | GDSC |
| ***PHLDA2*** | NPK76-II-72-1 | 0.3942 | 2.09E-23 | 7.35E-20 | 591 | GDSC |
| ***DNMT1*** | GSK429286A | -0.3935 | 2.73E-23 | 8.54E-20 | 590 | GDSC |
| ***PHLDA2*** | Vorinostat | 0.3920 | 8.30E-21 | 1.17E-17 | 527 | GDSC |
| ***DNMT1*** | NPK76-II-72-1 | -0.3914 | 4.52E-23 | 1.27E-19 | 591 | GDSC |
| ***DNMT1*** | TL-2-105 | -0.3868 | 1.71E-22 | 4.36E-19 | 590 | GDSC |
| ***TFPI2*** | Panobinostat | 0.3862 | 1.43E-14 | 2.57E-12 | 369 | CCLE |
| ***DNMT1*** | QL-XI-92 | -0.3847 | 3.04E-22 | 7.14E-19 | 590 | GDSC |
| ***DNMT1*** | BX-912 | -0.3830 | 4.76E-22 | 1.03E-18 | 590 | GDSC |
| ***BLCAP*** | Crizotinib | 0.3823 | 2.03E-09 | 8.44E-08 | 230 | GDSC |
| ***DNMT1*** | Selisistat | -0.3806 | 1.22E-21 | 2.35E-18 | 586 | GDSC |
| ***DNMT1*** | T0901317 | -0.3805 | 1.25E-21 | 2.35E-18 | 586 | GDSC |
| ***DNMT1*** | Irinotecan | -0.3802 | 3.77E-09 | 1.41E-07 | 225 | CCLE |
| ***GLIS3*** | Panobinostat | 0.3801 | 3.98E-14 | 6.37E-12 | 369 | CCLE |
| ***PHLDA2*** | Trametinib | -0.3773 | 1.61E-20 | 1.97E-17 | 564 | GDSC |
| ***DNMT1*** | GSK1070916 | -0.3771 | 6.69E-21 | 1.04E-17 | 576 | GDSC |
| ***DNMT1*** | PI-103 | -0.3766 | 3.75E-21 | 6.21E-18 | 585 | GDSC |
| ***DNMT1*** | Quizartinib | -0.3757 | 3.51E-21 | 6.18E-18 | 589 | GDSC |
| ***PHLDA2*** | Tanespimycin | -0.3748 | 5.07E-19 | 3.32E-16 | 527 | GDSC |
| ***PPP1R9A*** | XMD8-85 | 0.3739 | 9.71E-09 | 3.20E-07 | 221 | GDSC |
| ***DNMT1*** | Ispinesib Mesylate | -0.3733 | 7.04E-21 | 1.04E-17 | 588 | GDSC |
| ***SLC22A18*** | BX-912 | 0.3700 | 1.42E-20 | 1.90E-17 | 590 | GDSC |
| ***ANO1*** | GSK429286A | 0.3695 | 1.60E-20 | 1.97E-17 | 590 | GDSC |
| ***CPA4*** | XMD14-99 | 0.3692 | 1.70E-20 | 1.99E-17 | 590 | GDSC |
| ***CPA4*** | Amuvatinib | 0.3684 | 2.29E-20 | 2.58E-17 | 589 | GDSC |
| ***DNMT1*** | UNC1215 | -0.3674 | 8.76E-20 | 7.25E-17 | 574 | GDSC |
| ***PHLDA2*** | Fedratinib | 0.3673 | 2.78E-20 | 3.01E-17 | 590 | GDSC |
| ***DNMT1*** | GSK319347A | -0.3669 | 1.64E-08 | 5.07E-07 | 223 | GDSC |
| ***DNMT1*** | Y-39983 | -0.3667 | 3.03E-20 | 3.15E-17 | 591 | GDSC |
| ***DNMT1*** | CX-5461 | -0.3655 | 5.41E-20 | 5.25E-17 | 587 | GDSC |
| ***CPA4*** | TL-2-105 | 0.3650 | 4.94E-20 | 4.96E-17 | 590 | GDSC |
| ***GPR1*** | Panobinostat | 0.3645 | 4.86E-13 | 5.59E-11 | 369 | CCLE |
| ***DNMT1*** | STF-62247 | -0.3639 | 8.01E-20 | 7.07E-17 | 587 | GDSC |
| ***DNMT1*** | KIN001-260 | -0.3633 | 7.04E-20 | 6.60E-17 | 591 | GDSC |
| ***DNMT1*** | XMD14-99 | -0.3631 | 8.04E-20 | 7.07E-17 | 590 | GDSC |
| ***DNMT1*** | BMS-345541 | -0.3628 | 8.67E-20 | 7.25E-17 | 590 | GDSC |
| ***DNMT1*** | Ruxolitinib | -0.3619 | 1.14E-19 | 9.17E-17 | 589 | GDSC |
| ***DNMT1*** | VNLG/124 | -0.3612 | 1.68E-19 | 1.31E-16 | 586 | GDSC |
| ***PHLDA2*** | THZ-2-102-1 | 0.3605 | 2.66E-19 | 1.97E-16 | 582 | GDSC |
| ***HM13*** | Methotrexate | 0.3600 | 1.64E-17 | 7.45E-15 | 525 | GDSC |

| | | | | | | |
|---|---|---|---|---|---|---|
| ***DNMT1*** | WZ3105 | -0.3593 | 2.04E-19 | 1.55E-16 | 590 | GDSC |
| ***HM13*** | T0901317 | 0.3589 | 2.95E-19 | 2.13E-16 | 586 | GDSC |
| ***HM13*** | UNC1215 | 0.3588 | 7.08E-19 | 4.43E-16 | 574 | GDSC |
| ***GABRB3*** | Sunitinib | 0.3573 | 3.55E-08 | 9.78E-07 | 225 | GDSC |
| ***HM13*** | Tivozanib | 0.3569 | 3.88E-19 | 2.71E-16 | 589 | GDSC |
| ***PHLDA2*** | GSK1070916 | 0.3569 | 9.59E-19 | 5.74E-16 | 576 | GDSC |
| ***PHLDA2*** | I-BET-762 | 0.3568 | 4.58E-19 | 3.07E-16 | 587 | GDSC |
| ***PHLDA2*** | TL-2-105 | 0.3566 | 3.94E-19 | 2.71E-16 | 590 | GDSC |
| ***DNMT1*** | GSK690693 | -0.3556 | 5.77E-19 | 3.69E-16 | 588 | GDSC |
| ***HM13*** | Topotecan | 0.3547 | 1.68E-12 | 1.60E-10 | 373 | CCLE |
| ***CPA4*** | Vorinostat | 0.3547 | 4.59E-17 | 1.66E-14 | 527 | GDSC |
| ***PPP1R9A*** | Sunitinib | 0.3543 | 4.69E-08 | 1.23E-06 | 225 | GDSC |
| ***DLX5*** | XMD8-85 | 0.3540 | 6.33E-08 | 1.59E-06 | 221 | GDSC |
| ***CALCR*** | GSK319347A | 0.3534 | 5.82E-08 | 1.47E-06 | 223 | GDSC |
| ***TH*** | Sunitinib | 0.3534 | 5.09E-08 | 1.32E-06 | 225 | GDSC |
| ***DNMT1*** | XMD15-27 | -0.3521 | 1.17E-18 | 6.87E-16 | 590 | GDSC |
| ***PHLDA2*** | Refametinib | -0.3521 | 7.34E-19 | 4.49E-16 | 597 | GDSC |
| ***GLIS3*** | Imatinib | 0.3499 | 4.68E-08 | 1.23E-06 | 231 | GDSC |
| ***PHLDA2*** | BMS-345541 | 0.3494 | 2.19E-18 | 1.26E-15 | 590 | GDSC |
| ***SGCE*** | Imatinib | 0.3491 | 5.06E-08 | 1.31E-06 | 231 | GDSC |
| ***PHLDA2*** | Daporinad | 0.3487 | 1.03E-17 | 5.15E-15 | 569 | GDSC |
| ***DIRAS3*** | Imatinib | 0.3485 | 5.32E-08 | 1.37E-06 | 231 | GDSC |
| ***TH*** | Cyclopamine | 0.3473 | 1.16E-07 | 2.66E-06 | 221 | GDSC |
| ***SLC22A18*** | Irinotecan | 0.3470 | 9.12E-08 | 2.18E-06 | 225 | CCLE |
| ***PHLDA2*** | CAY10603 | 0.3466 | 5.50E-18 | 3.04E-15 | 586 | GDSC |
| ***GPR1*** | TAK-715 | 0.3462 | 4.67E-18 | 2.63E-15 | 590 | GDSC |
| ***TH*** | XMD8-85 | 0.3462 | 1.28E-07 | 2.89E-06 | 221 | GDSC |
| ***PHLDA2*** | XMD8-85 | 0.3454 | 1.37E-07 | 3.06E-06 | 221 | GDSC |
| ***CPA4*** | KIN001-260 | 0.3450 | 5.85E-18 | 3.17E-15 | 591 | GDSC |
| ***DGCR6*** | XMD8-85 | 0.3448 | 1.45E-07 | 3.22E-06 | 221 | GDSC |
| ***PHLDA2*** | JW-7-24-1 | 0.3446 | 6.84E-18 | 3.63E-15 | 590 | GDSC |
| ***GABRB3*** | Z-LLNle-CHO | 0.3443 | 1.16E-07 | 2.67E-06 | 225 | GDSC |
| ***DNMT1*** | KIN001-236 | -0.3434 | 8.40E-18 | 4.38E-15 | 591 | GDSC |
| ***GPR1*** | KIN001-236 | 0.3426 | 1.01E-17 | 5.15E-15 | 591 | GDSC |
| ***PHLDA2*** | TAK-715 | 0.3426 | 1.08E-17 | 5.32E-15 | 590 | GDSC |
| ***DNMT1*** | VX-702 | -0.3423 | 8.05E-16 | 2.10E-13 | 523 | GDSC |
| ***CPA4*** | MPS-1-IN-1 | 0.3421 | 1.14E-17 | 5.50E-15 | 591 | GDSC |
| ***GLIS3*** | Vorinostat | 0.3421 | 6.53E-16 | 1.77E-13 | 527 | GDSC |
| ***CPA4*** | NPK76-II-72-1 | 0.3420 | 1.17E-17 | 5.50E-15 | 591 | GDSC |
| ***PHLDA2*** | BIX02189 | 0.3420 | 1.17E-17 | 5.50E-15 | 591 | GDSC |
| ***DNMT1*** | Methotrexate | -0.3417 | 7.92E-16 | 2.08E-13 | 525 | GDSC |
| ***GPR1*** | T0901317 | 0.3411 | 1.95E-17 | 8.58E-15 | 586 | GDSC |
| ***PHLDA2*** | Crizotinib | 0.3405 | 1.19E-07 | 2.71E-06 | 230 | GDSC |
| ***DNMT1*** | Temozolomide | -0.3405 | 5.10E-17 | 1.77E-14 | 573 | GDSC |
| ***PHLDA2*** | YM201636 | 0.3405 | 1.67E-17 | 7.46E-15 | 591 | GDSC |
| ***DNMT1*** | CAY10603 | -0.3398 | 2.67E-17 | 1.14E-14 | 586 | GDSC |
| ***GLIS3*** | WZ3105 | 0.3394 | 2.24E-17 | 9.72E-15 | 590 | GDSC |
| ***DNMT1*** | Vorinostat | -0.3394 | 1.14E-15 | 2.84E-13 | 527 | GDSC |
| ***PPP1R9A*** | Imatinib | 0.3392 | 1.26E-07 | 2.84E-06 | 231 | GDSC |
| ***PEG10*** | AZ628 | 0.3392 | 1.96E-07 | 4.18E-06 | 224 | GDSC |
| ***CPA4*** | T0901317 | 0.3389 | 3.24E-17 | 1.32E-14 | 586 | GDSC |
| ***PHLDA2*** | XMD13-2 | 0.3384 | 2.85E-17 | 1.20E-14 | 590 | GDSC |

| | | | | | | |
|---|---|---|---|---|---|---|
| ***PHLDA2*** | OSI-027 | 0.3383 | 3.29E-17 | 1.32E-14 | 588 | GDSC |
| ***DNMT1*** | QL-X-138 | -0.3382 | 4.32E-17 | 1.60E-14 | 584 | GDSC |
| ***CPA4*** | KIN001-236 | 0.3380 | 2.95E-17 | 1.22E-14 | 591 | GDSC |
| ***CPA4*** | I-BET-762 | 0.3379 | 3.79E-17 | 1.48E-14 | 587 | GDSC |
| ***CPA4*** | TAK-715 | 0.3371 | 3.82E-17 | 1.48E-14 | 590 | GDSC |
| ***HM13*** | XMD14-99 | 0.3371 | 3.83E-17 | 1.48E-14 | 590 | GDSC |
| ***PHLDA2*** | L-685458 | 0.3369 | 5.30E-11 | 3.38E-09 | 360 | CCLE |
| ***DNMT1*** | GNF-2 | -0.3368 | 2.26E-07 | 4.74E-06 | 225 | GDSC |
| ***PHLDA2*** | GSK429286A | 0.3366 | 4.30E-17 | 1.60E-14 | 590 | GDSC |
| ***PHLDA2*** | KIN001-260 | 0.3362 | 4.44E-17 | 1.62E-14 | 591 | GDSC |
| ***PHLDA2*** | GSK690693 | 0.3360 | 5.50E-17 | 1.87E-14 | 588 | GDSC |
| ***DNMT1*** | TAK-715 | -0.3359 | 5.03E-17 | 1.77E-14 | 590 | GDSC |
| ***GPR1*** | Tubastatin A | 0.3357 | 6.29E-17 | 2.06E-14 | 587 | GDSC |
| ***CPA4*** | BMS-345541 | 0.3357 | 5.28E-17 | 1.81E-14 | 590 | GDSC |
| ***HM13*** | NPK76-II-72-1 | 0.3356 | 4.99E-17 | 1.77E-14 | 591 | GDSC |
| ***BLCAP*** | Salubrinal | 0.3356 | 3.03E-07 | 6.07E-06 | 222 | GDSC |
| ***CPA4*** | OSI-930 | 0.3355 | 5.79E-17 | 1.94E-14 | 589 | GDSC |
| ***GPR1*** | TL-2-105 | 0.3350 | 6.08E-17 | 2.01E-14 | 590 | GDSC |
| ***DNMT1*** | GW-2580 | -0.3346 | 6.75E-17 | 2.18E-14 | 590 | GDSC |
| ***SGCE*** | Crizotinib | 0.3342 | 2.10E-07 | 4.44E-06 | 230 | GDSC |
| ***PHLDA2*** | TPCA-1 | 0.3341 | 7.08E-17 | 2.26E-14 | 591 | GDSC |
| ***DNMT1*** | Topotecan | -0.3334 | 3.92E-11 | 2.60E-09 | 373 | CCLE |
| ***BLCAP*** | Cyclopamine | 0.3332 | 3.95E-07 | 7.66E-06 | 221 | GDSC |
| ***HM13*** | PFI-3 | 0.3328 | 1.66E-15 | 3.99E-13 | 543 | GDSC |
| ***DNMT1*** | Vismodegib | -0.3327 | 4.60E-15 | 1.01E-12 | 526 | GDSC |
| ***GPR1*** | XMD14-99 | 0.3322 | 1.15E-16 | 3.63E-14 | 590 | GDSC |
| ***DNMT1*** | Linifanib | -0.3316 | 1.31E-16 | 4.10E-14 | 590 | GDSC |
| ***PHLDA2*** | T0901317 | 0.3315 | 1.68E-16 | 5.10E-14 | 586 | GDSC |
| ***HM13*** | Axitinib | 0.3314 | 7.09E-15 | 1.44E-12 | 523 | GDSC |
| ***DNMT1*** | Masitinib | -0.3314 | 1.45E-16 | 4.49E-14 | 589 | GDSC |
| ***HM13*** | Tubastatin A | 0.3313 | 1.66E-16 | 5.09E-14 | 587 | GDSC |
| ***PHLDA2*** | Navitoclax | 0.3310 | 6.15E-15 | 1.28E-12 | 527 | GDSC |
| ***PPP1R9A*** | AICA Ribonucleotide | 0.3301 | 1.17E-14 | 2.17E-12 | 519 | GDSC |
| ***CALCR*** | Cyclopamine | 0.3300 | 5.18E-07 | 9.68E-06 | 221 | GDSC |
| ***DNMT1*** | SNX-2112 | -0.3298 | 2.18E-16 | 6.46E-14 | 588 | GDSC |
| ***CPA4*** | Methotrexate | 0.3295 | 9.16E-15 | 1.75E-12 | 525 | GDSC |
| ***PHLDA2*** | Tubastatin A | 0.3295 | 2.46E-16 | 7.21E-14 | 587 | GDSC |
| ***DGCR6*** | Sunitinib | 0.3295 | 4.26E-07 | 8.19E-06 | 225 | GDSC |
| ***DNMT1*** | UNC0638 | -0.3295 | 4.09E-17 | 1.56E-14 | 618 | GDSC |
| ***DNMT1*** | PHA-793887 | -0.3293 | 2.17E-16 | 6.46E-14 | 590 | GDSC |
| ***DNMT1*** | I-BET-762 | -0.3292 | 2.65E-16 | 7.68E-14 | 587 | GDSC |
| ***PHLDA2*** | PD0325901 | -0.3287 | 1.29E-14 | 2.37E-12 | 522 | GDSC |
| ***PHLDA2*** | Panobinostat | 0.3283 | 1.01E-10 | 5.97E-09 | 369 | CCLE |
| ***PHLDA2*** | Axitinib | 0.3269 | 1.71E-14 | 3.01E-12 | 523 | GDSC |
| ***CALCR*** | Crizotinib | 0.3268 | 4.00E-07 | 7.74E-06 | 230 | GDSC |
| ***DNMT1*** | OSI-027 | -0.3267 | 4.28E-16 | 1.22E-13 | 588 | GDSC |
| ***DDC*** | Dasatinib | 0.3266 | 6.13E-07 | 1.12E-05 | 223 | GDSC |
| ***SGCE*** | PHA-665752 | 0.3265 | 3.87E-07 | 7.52E-06 | 231 | GDSC |
| ***GPR1*** | KIN001-260 | 0.3265 | 3.80E-16 | 1.09E-13 | 591 | GDSC |
| ***TH*** | Salubrinal | 0.3265 | 6.56E-07 | 1.18E-05 | 222 | GDSC |
| ***DNMT1*** | THZ-2-102-1 | -0.3263 | 6.69E-16 | 1.79E-13 | 582 | GDSC |
| ***SGCE*** | Sunitinib | 0.3261 | 5.66E-07 | 1.04E-05 | 225 | GDSC |

| | | | | | | |
|---|---|---|---|---|---|---|
| ***PHLDA2*** | PFI-3 | 0.3259 | 6.66E-15 | 1.38E-12 | 543 | GDSC |
| ***PHLDA2*** | Quizartinib | 0.3255 | 5.24E-16 | 1.46E-13 | 589 | GDSC |
| ***PHLDA2*** | CP466722 | 0.3255 | 5.25E-16 | 1.46E-13 | 589 | GDSC |
| ***HM13*** | Quizartinib | 0.3253 | 5.48E-16 | 1.51E-13 | 589 | GDSC |
| ***DNMT1*** | PFI-3 | -0.3252 | 7.70E-15 | 1.52E-12 | 543 | GDSC |
| ***TFPI2*** | I-BET-762 | 0.3251 | 6.53E-16 | 1.77E-13 | 587 | GDSC |
| ***HM13*** | GSK429286A | 0.3239 | 7.04E-16 | 1.87E-13 | 590 | GDSC |
| ***CPA4*** | Crizotinib | 0.3239 | 5.12E-07 | 9.59E-06 | 230 | GDSC |
| ***GPR1*** | Methotrexate | 0.3238 | 2.79E-14 | 4.70E-12 | 525 | GDSC |
| ***GPR1*** | GSK319347A | 0.3233 | 8.05E-07 | 1.41E-05 | 223 | GDSC |
| ***GPR1*** | OSI-930 | 0.3233 | 8.51E-16 | 2.18E-13 | 589 | GDSC |
| ***PHLDA2*** | PIK-93 | 0.3230 | 8.13E-16 | 2.10E-13 | 591 | GDSC |
| ***GPR1*** | Masitinib | 0.3229 | 9.36E-16 | 2.37E-13 | 589 | GDSC |
| ***SGCE*** | Vorinostat | 0.3221 | 3.47E-14 | 5.68E-12 | 527 | GDSC |
| ***CPA4*** | XMD13-2 | 0.3220 | 1.07E-15 | 2.68E-13 | 590 | GDSC |
| ***GPR1*** | Amuvatinib | 0.3216 | 1.24E-15 | 3.03E-13 | 589 | GDSC |
| ***HM13*** | XMD13-2 | 0.3215 | 1.17E-15 | 2.89E-13 | 590 | GDSC |
| ***AIRN*** | Crizotinib | -0.3213 | 6.39E-07 | 1.15E-05 | 230 | GDSC |
| ***GPR1*** | Crizotinib | 0.3210 | 6.55E-07 | 1.18E-05 | 230 | GDSC |
| ***DNMT1*** | TL-1-85 | -0.3208 | 1.31E-15 | 3.17E-13 | 591 | GDSC |
| ***HM13*** | Panobinostat | 0.3205 | 2.91E-10 | 1.55E-08 | 369 | CCLE |
| ***TH*** | AZD7762 | 0.3205 | 4.95E-14 | 7.62E-12 | 526 | GDSC |
| ***GPR1*** | Vorinostat | 0.3205 | 4.71E-14 | 7.28E-12 | 527 | GDSC |
| ***CPA4*** | Tubastatin A | 0.3202 | 1.86E-15 | 4.43E-13 | 587 | GDSC |
| ***GABRB3*** | WZ-1-84 | 0.3201 | 9.38E-07 | 1.60E-05 | 225 | GDSC |
| ***DLX5*** | Z-LLNle-CHO | 0.3199 | 9.50E-07 | 1.62E-05 | 225 | GDSC |
| ***DGCR6*** | Crizotinib | 0.3198 | 7.27E-07 | 1.29E-05 | 230 | GDSC |
| ***GABRB3*** | XMD8-85 | 0.3194 | 1.24E-06 | 2.06E-05 | 221 | GDSC |
| ***HM13*** | Ruxolitinib | 0.3194 | 1.96E-15 | 4.59E-13 | 589 | GDSC |
| ***PHLDA2*** | PHA-793887 | 0.3193 | 1.89E-15 | 4.47E-13 | 590 | GDSC |
| ***PPP1R9A*** | Z-LLNle-CHO | 0.3193 | 1.00E-06 | 1.70E-05 | 225 | GDSC |
| ***HM13*** | TL-2-105 | 0.3188 | 2.10E-15 | 4.85E-13 | 590 | GDSC |
| ***PHLDA2*** | Methotrexate | 0.3186 | 7.49E-14 | 1.11E-11 | 525 | GDSC |
| ***HM13*** | Zibotentan | 0.3186 | 2.09E-15 | 4.85E-13 | 591 | GDSC |
| ***PHLDA2*** | KIN001-270 | 0.3184 | 2.16E-15 | 4.94E-13 | 591 | GDSC |
| ***PEG10*** | Crizotinib | 0.3182 | 8.29E-07 | 1.45E-05 | 230 | GDSC |
| ***TH*** | Crizotinib | 0.3181 | 8.40E-07 | 1.46E-05 | 230 | GDSC |
| ***DLK1*** | Z-LLNle-CHO | 0.3178 | 1.14E-06 | 1.90E-05 | 225 | GDSC |
| ***PHLDA2*** | Belinostat | 0.3174 | 7.01E-15 | 1.43E-12 | 573 | GDSC |
| ***PHLDA2*** | PD-0332991 | 0.3174 | 8.36E-09 | 2.82E-07 | 315 | CCLE |
| ***DNMT1*** | Cyclopamine | -0.3174 | 1.46E-06 | 2.36E-05 | 221 | GDSC |
| ***PHLDA2*** | AR-42 | 0.3173 | 4.18E-15 | 9.27E-13 | 583 | GDSC |
| ***GPR1*** | STF-62247 | 0.3172 | 3.43E-15 | 7.78E-13 | 587 | GDSC |
| ***TH*** | Lestaurtinib | 0.3172 | 9.72E-14 | 1.39E-11 | 525 | GDSC |
| ***DNMT1*** | Alectinib | -0.3170 | 3.59E-15 | 8.08E-13 | 587 | GDSC |
| ***PPP1R9A*** | Cyclopamine | 0.3169 | 1.52E-06 | 2.44E-05 | 221 | GDSC |
| ***SGCE*** | Methotrexate | 0.3169 | 1.04E-13 | 1.48E-11 | 525 | GDSC |
| ***DDC*** | Sunitinib | 0.3166 | 1.25E-06 | 2.07E-05 | 225 | GDSC |
| ***PHLDA2*** | Parthenolide | 0.3166 | 1.47E-06 | 2.37E-05 | 222 | GDSC |
| ***PHLDA2*** | QL-X-138 | 0.3166 | 4.62E-15 | 1.01E-12 | 584 | GDSC |
| ***GLIS3*** | Methotrexate | 0.3165 | 1.12E-13 | 1.57E-11 | 525 | GDSC |
| ***DNMT1*** | SGC0946 | -0.3164 | 8.71E-15 | 1.69E-12 | 573 | GDSC |

| | | | | | | |
|---|---|---|---|---|---|---|
| ***GPR1*** | Imatinib | 0.3164 | 9.15E-07 | 1.57E-05 | 231 | GDSC |
| ***PHLDA2*** | NG-25 | 0.3154 | 4.05E-15 | 9.05E-13 | 591 | GDSC |
| ***CPA4*** | VX-702 | 0.3153 | 1.55E-13 | 2.07E-11 | 523 | GDSC |
| ***DNMT1*** | S-Trityl-L-cysteine | -0.3151 | 1.41E-06 | 2.28E-05 | 225 | GDSC |
| ***DNMT1*** | FMK | -0.3150 | 1.13E-13 | 1.58E-11 | 530 | GDSC |
| ***PHLDA2*** | PI-103 | 0.3150 | 6.15E-15 | 1.28E-12 | 585 | GDSC |
| ***HM13*** | BX-912 | 0.3149 | 4.72E-15 | 1.02E-12 | 590 | GDSC |
| ***GPR1*** | NVP-BHG712 | 0.3147 | 4.75E-15 | 1.02E-12 | 591 | GDSC |
| ***DNMT1*** | JW-7-24-1 | -0.3144 | 5.27E-15 | 1.12E-12 | 590 | GDSC |
| ***GABRB3*** | CGP-60474 | 0.3143 | 1.50E-06 | 2.40E-05 | 225 | GDSC |
| ***DNMT1*** | Nilotinib | -0.3143 | 5.35E-13 | 6.10E-11 | 503 | GDSC |
| ***DNMT1*** | NSC-207895 | -0.3143 | 6.71E-15 | 1.38E-12 | 586 | GDSC |
| ***SLC22A18*** | NPK76-II-72-1 | 0.3142 | 5.28E-15 | 1.12E-12 | 591 | GDSC |
| ***DNMT1*** | QL-XII-61 | -0.3141 | 8.83E-08 | 2.12E-06 | 278 | GDSC |
| ***DNMT1*** | Belinostat | -0.3137 | 1.51E-14 | 2.67E-12 | 573 | GDSC |
| ***DDC*** | QL-VIII-58 | 0.3135 | 3.57E-08 | 9.83E-07 | 296 | GDSC |
| ***DDC*** | TGX221 | 0.3133 | 1.82E-06 | 2.85E-05 | 223 | GDSC |
| ***SGCE*** | TAK-715 | 0.3129 | 7.27E-15 | 1.46E-12 | 590 | GDSC |
| ***DNMT1*** | OSI-930 | -0.3128 | 7.73E-15 | 1.52E-12 | 589 | GDSC |
| ***SGCE*** | 5-Fluorouracil | 0.3127 | 9.20E-15 | 1.75E-12 | 586 | GDSC |
| ***SLC22A18AS*** | KIN001-270 | 0.3124 | 7.63E-15 | 1.52E-12 | 591 | GDSC |
| ***GPR1*** | BMS-345541 | 0.3121 | 8.59E-15 | 1.68E-12 | 590 | GDSC |
| ***PHLDA2*** | WZ3105 | 0.3119 | 8.81E-15 | 1.70E-12 | 590 | GDSC |
| ***HM13*** | Vorinostat | 0.3118 | 2.39E-13 | 3.01E-11 | 527 | GDSC |
| ***HM13*** | VX-702 | 0.3116 | 3.06E-13 | 3.76E-11 | 523 | GDSC |
| ***GPR1*** | I-BET-762 | 0.3115 | 1.13E-14 | 2.14E-12 | 587 | GDSC |
| ***CPA4*** | AR-42 | 0.3112 | 1.49E-14 | 2.65E-12 | 583 | GDSC |
| ***HM13*** | SGC0946 | 0.3110 | 2.59E-14 | 4.45E-12 | 573 | GDSC |
| ***GPR1*** | Quizartinib | 0.3109 | 1.16E-14 | 2.17E-12 | 589 | GDSC |
| ***H19*** | KIN001-260 | 0.3103 | 1.18E-14 | 2.18E-12 | 591 | GDSC |
| ***DGCR6*** | S-Trityl-L-cysteine | 0.3102 | 2.09E-06 | 3.18E-05 | 225 | GDSC |
| ***TFPI2*** | Methotrexate | 0.3102 | 3.57E-13 | 4.28E-11 | 525 | GDSC |
| ***TFPI2*** | Amuvatinib | 0.3099 | 1.40E-14 | 2.56E-12 | 589 | GDSC |
| ***CPA4*** | Quizartinib | 0.3099 | 1.42E-14 | 2.57E-12 | 589 | GDSC |
| ***HM13*** | Imatinib | 0.3098 | 1.57E-06 | 2.51E-05 | 231 | GDSC |
| ***MAGI2*** | WZ-1-84 | 0.3092 | 2.26E-06 | 3.40E-05 | 225 | GDSC |
| ***PHLDA2*** | TL-1-85 | 0.3092 | 1.47E-14 | 2.64E-12 | 591 | GDSC |
| ***SLC22A18AS*** | PI-103 | 0.3086 | 2.25E-14 | 3.91E-12 | 585 | GDSC |
| ***DGCR6*** | CGP-082996 | 0.3086 | 2.37E-06 | 3.53E-05 | 225 | GDSC |
| ***PHLDA2*** | QL-XI-92 | 0.3083 | 1.85E-14 | 3.23E-12 | 590 | GDSC |
| ***HM13*** | Linifanib | 0.3073 | 2.27E-14 | 3.92E-12 | 590 | GDSC |
| ***PEG10*** | Imatinib | 0.3073 | 1.93E-06 | 2.99E-05 | 231 | GDSC |
| ***PHLDA2*** | VNLG/124 | 0.3069 | 3.00E-14 | 5.00E-12 | 586 | GDSC |
| ***HM13*** | Daporinad | 0.3069 | 7.11E-14 | 1.06E-11 | 569 | GDSC |
| ***PHLDA2*** | CUDC-101 | 0.3068 | 4.39E-14 | 6.87E-12 | 579 | GDSC |
| ***DNMT1*** | Axitinib | -0.3065 | 7.72E-13 | 8.42E-11 | 523 | GDSC |
| ***SLC22A18*** | Fedratinib | 0.3065 | 2.69E-14 | 4.59E-12 | 590 | GDSC |
| ***BLCAP*** | Lestaurtinib | 0.3063 | 7.29E-13 | 8.04E-11 | 525 | GDSC |
| ***GLIS3*** | XMD13-2 | 0.3062 | 2.82E-14 | 4.73E-12 | 590 | GDSC |
| ***CPA4*** | TPCA-1 | 0.3060 | 2.79E-14 | 4.70E-12 | 591 | GDSC |
| ***RB1*** | Palbociclib | -0.3060 | 1.79E-12 | 1.68E-10 | 508 | GDSC |
| ***PHLDA2*** | Amuvatinib | 0.3059 | 3.19E-14 | 5.24E-12 | 589 | GDSC |

| | | | | | | |
|---|---|---|---|---|---|---|
| ***SLC22A18*** | QL-XI-92 | 0.3058 | 3.10E-14 | 5.13E-12 | 590 | GDSC |
| ***HM13*** | AR-42 | 0.3056 | 4.51E-14 | 7.01E-12 | 583 | GDSC |
| ***HM13*** | Ispinesib Mesylate | 0.3056 | 3.52E-14 | 5.73E-12 | 588 | GDSC |
| ***DDC*** | JW-7-52-1 | 0.3056 | 3.49E-06 | 4.92E-05 | 222 | GDSC |
| ***CPA4*** | Phenformin | 0.3054 | 5.01E-14 | 7.66E-12 | 582 | GDSC |
| ***DNMT1*** | AR-42 | -0.3049 | 5.24E-14 | 7.94E-12 | 583 | GDSC |
| ***TFPI2*** | BMS-345541 | 0.3049 | 3.71E-14 | 6.00E-12 | 590 | GDSC |
| ***SLC22A18*** | PD173074 | 0.3048 | 9.11E-13 | 9.56E-11 | 526 | GDSC |
| ***TFPI2*** | XMD14-99 | 0.3047 | 3.83E-14 | 6.15E-12 | 590 | GDSC |
| ***GLIS3*** | Amuvatinib | 0.3046 | 4.13E-14 | 6.55E-12 | 589 | GDSC |
| ***ANO1*** | Ruxolitinib | 0.3046 | 4.14E-14 | 6.55E-12 | 589 | GDSC |
| ***CPA4*** | Imatinib | 0.3046 | 2.40E-06 | 3.57E-05 | 231 | GDSC |
| ***PEG10*** | Sunitinib | 0.3043 | 3.32E-06 | 4.73E-05 | 225 | GDSC |
| ***BLCAP*** | PD173074 | 0.3043 | 9.91E-13 | 1.03E-10 | 526 | GDSC |
| ***PHLDA2*** | Entinostat | 0.3041 | 3.73E-06 | 5.18E-05 | 223 | GDSC |
| ***GPR1*** | KIN001-244 | 0.3038 | 4.35E-14 | 6.84E-12 | 591 | GDSC |
| ***PHLDA2*** | AZD8055 | 0.3037 | 1.33E-12 | 1.30E-10 | 522 | GDSC |
| ***ANO1*** | KIN001-260 | 0.3030 | 5.10E-14 | 7.76E-12 | 591 | GDSC |
| ***BLCAP*** | BX795 | 0.3030 | 1.19E-12 | 1.18E-10 | 527 | GDSC |
| ***SLC22A18AS*** | QL-X-138 | 0.3028 | 7.51E-14 | 1.11E-11 | 584 | GDSC |
| ***CPA4*** | GSK1070916 | 0.3027 | 1.15E-13 | 1.59E-11 | 576 | GDSC |
| ***DNMT1*** | CP466722 | -0.3026 | 6.11E-14 | 9.20E-12 | 589 | GDSC |
| ***PHLDA2*** | PD173074 | 0.3025 | 1.37E-12 | 1.34E-10 | 526 | GDSC |
| ***GABRB3*** | Cyclopamine | 0.3021 | 4.82E-06 | 6.49E-05 | 221 | GDSC |
| ***HM13*** | QL-XI-92 | 0.3019 | 6.77E-14 | 1.01E-11 | 590 | GDSC |
| ***CPA4*** | Masitinib | 0.3015 | 7.67E-14 | 1.12E-11 | 589 | GDSC |
| ***PHLDA2*** | ZG-10 | 0.3014 | 1.31E-07 | 2.95E-06 | 295 | GDSC |
| ***PEG10*** | PHA-665752 | 0.3012 | 3.12E-06 | 4.48E-05 | 231 | GDSC |
| ***SLC22A18*** | Tubastatin A | 0.3010 | 9.35E-14 | 1.34E-11 | 587 | GDSC |
| ***DNMT1*** | Navitoclax | -0.3008 | 1.74E-12 | 1.65E-10 | 527 | GDSC |
| ***ANO1*** | Cyclopamine | 0.3007 | 5.35E-06 | 7.09E-05 | 221 | GDSC |
| ***PHLDA2*** | Y-39983 | 0.3006 | 8.23E-14 | 1.20E-11 | 591 | GDSC |
| ***HM13*** | XMD15-27 | 0.3006 | 8.65E-14 | 1.25E-11 | 590 | GDSC |
| ***TH*** | TL-1-85 | 0.3005 | 8.41E-14 | 1.22E-11 | 591 | GDSC |

Correlation results for the genes *BLCAP* and *HM13* located on 20q11-q13.3 are highlighted in yellow

**Sample size,** number of cell lines with available data used in correlation analysis
**Spearman ρ,** Spearman correlation coefficient
$p_0$, *p*- value prior to FDR adjustment
$p_{FDR}$, *p* -value after FDR adjustment
**Drug response data source,** dataset (GDSC or CCLE) from which the drug response values were obtained
