# Supplementary material for: Increased copy number of imprinted genes in the chromosomal region 20q11-q13.32 is associated with resistance to antitumor agents in cancer cell lines
Source: Clin Epigenetics. 2022 Dec 2;14:161. doi: 10.1186/s13148-022-01368-7 (PMC9716673; doi:10.1186/s13148-022-01368-7)
Supplement: Supplementary file 9 — Additional file 9: Table S6. Methylation of imprinted gene regions associated with log(IC50) satisfying |ρ| > 0.3 and pFDR < 0.05. Spearman ρ, Spearman correlation coefficient. The results are sorted by the absolute value of |ρ|. p0, p value prior to FDR adjustment. pFDR, p value after FDR adjustment. Drug response data source, dataset (GDSC or CCLE) from which the drug response values were obtained. Sample size, number of cell lines with available data used in correlation analysis. Cytoband, chromosomal region location according to the UCSC genome annotation database for the hg19 (GRCh37) assembly of the human genome based on the probe coordinates in the Illumina Infinium HumanMethylation 450K BeadChip annotation. The 6 gene regions include TSS1500, TSS200, 5′ UTR (UTR5), 1st exon (EXON1), gene body (GENE BODY), and 3' UTR (UTR3). [file 13148_2022_1368_MOESM9_ESM.pdf]

Table S6. Methylation of imprinted gene regions associated with log(IC50) satisfying $|\rho| > 0.3$ and $p_{FDR} < 0.05$

Gene	Methylation	Agent	Spearman ρ	p_0	p_{FDR}	Cytoband	Sample size	Drug response data source
ANO1	EXON1	XMD8-85	-0.4021	5.40E-10	1.69E-07	11q13.3	221	GDSC
DLX5	UTR5	XMD8-85	-0.3865	2.74E-09	5.65E-07	7q21.3	221	GDSC
NLRP2	UTR5	WZ-1-84	-0.3844	2.46E-09	5.22E-07	19q13.42	225	GDSC
DIO3	TSS200	XMD8-85	-0.3744	9.18E-09	1.39E-06	14q32.31	221	GDSC
DLX5	TSS200	XMD8-85	-0.3744	9.21E-09	1.39E-06	7q21.3	221	GDSC
NLRP2	UTR5	XMD8-85	-0.3729	1.06E-08	1.55E-06	19q13.42	221	GDSC
MAGEL2	UTR3	XMD8-85	-0.3719	1.17E-08	1.67E-06	15q11.2	221	GDSC
PLAGL1	UTR3	XMD8-85	-0.3707	1.32E-08	1.82E-06	6q24.2	221	GDSC
DLX5	EXON1	XMD8-85	-0.3697	1.46E-08	1.97E-06	7q21.3	221	GDSC
DDC	UTR3	Temsirolimus	0.3682	4.18E-18	2.96E-13	7p12.1	519	GDSC
ZIM3	UTR3	XMD8-85	-0.3660	2.23E-08	2.73E-06	19q13.43	220	GDSC
SNRPN	UTR5	WZ-1-84	-0.3655	1.62E-08	2.15E-06	15q11.2	225	GDSC
DDC	TSS1500	Temsirolimus	0.3613	1.90E-17	3.84E-13	7p12.1	519	GDSC
GABRG3	UTR5	Z-LLNle-CHO	-0.3602	2.68E-08	3.16E-06	15q12	225	GDSC
GABRG3	EXON1	Z-LLNle-CHO	-0.3602	2.68E-08	3.16E-06	15q12	225	GDSC
MIR371	TSS200	WZ-1-84	-0.3600	2.74E-08	3.20E-06	19q13.42	225	GDSC
ANO1	EXON1	Sunitinib	-0.3597	2.81E-08	3.27E-06	11q13.3	225	GDSC
PHLDA2	TSS200	CGP-082996	-0.3594	2.89E-08	3.34E-06	11p15.4	225	GDSC
ZIM3	GENE BODY	XMD8-85	-0.3589	4.34E-08	4.54E-06	19q13.43	220	GDSC
PPP1R9A	UTR5	Imatinib	-0.3579	2.19E-08	2.70E-06	7q21.3	231	GDSC
DIO3	TSS200	Cyclopamine	-0.3576	4.57E-08	4.74E-06	14q32.31	221	GDSC
ANO1	EXON1	Cyclopamine	-0.3572	4.71E-08	4.85E-06	11q13.3	221	GDSC
NTM	UTR5	WZ-1-84	-0.3560	3.99E-08	4.25E-06	11q25	225	GDSC
NTM	EXON1	WZ-1-84	-0.3560	3.99E-08	4.25E-06	11q25	225	GDSC
NTM	UTR5	XMD8-85	-0.3559	5.31E-08	5.31E-06	11q25	221	GDSC
NTM	EXON1	XMD8-85	-0.3559	5.31E-08	5.31E-06	11q25	221	GDSC
AIM1	GENE BODY	Sunitinib	0.3534	5.07E-08	5.17E-06	6q21	225	GDSC
PLAGL1	UTR3	GNF-2	-0.3529	5.31E-08	5.31E-06	6q24.2	225	GDSC
CDKN1C	EXON1	Cyclopamine	-0.3529	7.03E-08	6.62E-06	11p15.4	221	GDSC
PHLDA2	UTR5	CGP-082996	-0.3520	5.80E-08	5.67E-06	11p15.4	225	GDSC
CALCR	TSS200	XMD8-85	-0.3499	9.20E-08	8.17E-06	7q21.3	221	GDSC
GABRB3	EXON1	Z-LLNle-CHO	-0.3495	7.28E-08	6.81E-06	15q12	225	GDSC
PHLDA2	UTR3	BX-912	-0.3488	2.53E-18	2.96E-13	11p15.4	590	GDSC
MAGI2	UTR3	Nutlin-3a (-)	-0.3476	2.06E-16	2.08E-12	7q21.11	527	GDSC
LRRTM1	EXON1	WZ-1-84	-0.3470	9.16E-08	8.15E-06	2p12	225	GDSC
CPA4	UTR5	Panobinostat	-0.3456	8.59E-12	7.24E-09	7q32.2	369	CCLE
CPA4	EXON1	Panobinostat	-0.3456	8.59E-12	7.24E-09	7q32.2	369	CCLE
GABRG3	TSS200	WZ-1-84	-0.3445	1.15E-07	9.67E-06	15q12	225	GDSC
GABRG3	UTR5	WZ-1-84	-0.3445	1.15E-07	9.67E-06	15q12	225	GDSC
GABRG3	EXON1	WZ-1-84	-0.3445	1.15E-07	9.67E-06	15q12	225	GDSC
DIO3	UTR5	Cyclopamine	-0.3441	1.54E-07	1.21E-05	14q32.31	221	GDSC
CDKN1C	EXON1	XMD8-85	-0.3439	1.57E-07	1.23E-05	11p15.4	221	GDSC
PHLDA2	UTR5	XMD8-85	-0.3435	1.62E-07	1.26E-05	11p15.4	221	GDSC
CDKN1C	EXON1	Z-LLNle-CHO	-0.3433	1.27E-07	1.05E-05	11p15.4	225	GDSC
DDC	UTR5	Temsirolimus	0.3433	8.40E-16	5.44E-12	7p12.1	519	GDSC
AIM1	GENE BODY	AICA Ribonucleotide	0.3433	8.47E-16	5.44E-12	6q21	519	GDSC
USP29	TSS200	WZ-1-84	-0.3427	1.44E-07	1.15E-05	19q13.43	224	GDSC
SLC22A3	EXON1	KIN001-270	-0.3422	1.11E-17	3.78E-13	6q25.3	591	GDSC
BLCAP	EXON1	Cyclopamine	-0.3418	1.88E-07	1.40E-05	20q11.23	221	GDSC
SLC22A3	EXON1	GSK319347A	-0.3412	1.75E-07	1.33E-05	6q25.3	223	GDSC
PHLDA2	UTR3	GSK429286A	-0.3409	1.60E-17	3.78E-13	11p15.4	590	GDSC
SLC22A18	TSS1500	PLX-4720	0.3408	9.32E-18	3.78E-13	11p15.4	599	GDSC
DIO3	UTR5	XMD8-85	-0.3408	2.06E-07	1.49E-05	14q32.31	221	GDSC
AIM1	GENE BODY	Z-LLNle-CHO	0.3405	1.64E-07	1.27E-05	6q21	225	GDSC
CDKN1C	GENE BODY	XMD8-85	-0.3403	2.16E-07	1.53E-05	11p15.4	221	GDSC
SLC22A18AS	GENE BODY	PLX-4720	0.3392	1.35E-17	3.78E-13	11p15.4	599	GDSC
USP29	TSS1500	WZ-1-84	-0.3392	1.84E-07	1.38E-05	19q13.43	225	GDSC
NTM	UTR5	CGP-082996	-0.3385	1.95E-07	1.44E-05	11q25	225	GDSC
NTM	EXON1	CGP-082996	-0.3385	1.95E-07	1.44E-05	11q25	225	GDSC
PHLDA2	EXON1	CGP-082996	-0.3378	2.08E-07	1.50E-05	11p15.4	225	GDSC
NNAT	GENE BODY	Cyclopamine	-0.3377	2.69E-07	1.82E-05	20q11.23	221	GDSC
SLC22A3	EXON1	QL-X-138	-0.3377	4.80E-17	6.80E-13	6q25.3	584	GDSC
PHLDA2	TSS200	XMD8-85	-0.3375	2.75E-07	1.84E-05	11p15.4	221	GDSC
PPP1R9A	UTR5	XMD8-85	-0.3373	2.79E-07	1.86E-05	7q21.3	221	GDSC
SGK2	TSS200	ZG-10	-0.3372	2.82E-09	5.79E-07	20q13.12	295	GDSC
PHLDA2	UTR5	Crizotinib	-0.3372	1.61E-07	1.25E-05	11p15.4	230	GDSC
PHLDA2	UTR3	JW-7-24-1	-0.3369	3.96E-17	6.23E-13	11p15.4	590	GDSC
PHACTR2	GENE BODY	XMD8-85	-0.3359	3.15E-07	2.05E-05	6q24.2	221	GDSC
NLRP2	UTR5	Sunitinib	-0.3359	2.46E-07	1.69E-05	19q13.42	225	GDSC
USP29	TSS200	XMD8-85	-0.3358	3.39E-07	2.17E-05	19q13.43	220	GDSC
PHLDA2	EXON1	Crizotinib	-0.3356	1.85E-07	1.38E-05	11p15.4	230	GDSC
GABRB3	UTR5	Z-LLNle-CHO	-0.3355	2.54E-07	1.74E-05	15q12	225	GDSC
SLC22A3	TSS200	QL-X-138	-0.3355	7.91E-17	8.62E-13	6q25.3	584	GDSC
PHLDA2	EXON1	XMD8-85	-0.3353	3.32E-07	2.13E-05	11p15.4	221	GDSC
SLC22A3	EXON1	Zibotentan	-0.3346	6.27E-17	8.08E-13	6q25.3	591	GDSC
CDKN1C	UTR5	Cyclopamine	-0.3346	3.51E-07	2.22E-05	11p15.4	221	GDSC
GRB10	EXON1	Cyclopamine	-0.3345	3.54E-07	2.24E-05	7p12.1	221	GDSC
NTM	UTR5	Z-LLNle-CHO	-0.3342	2.83E-07	1.89E-05	11q25	225	GDSC
NTM	EXON1	Z-LLNle-CHO	-0.3342	2.83E-07	1.89E-05	11q25	225	GDSC

SLC22A3	EXON1	KIN001-236	-0.3339	7.46E-17	8.62E-13	6q25.3	591	GDSC
BLCAP	EXON1	GSK269962A	-0.3333	2.69E-17	4.77E-13	20q11.23	610	GDSC
CDKN1C	EXON1	CGP-082996	-0.3329	3.19E-07	2.07E-05	11p15.4	225	GDSC
CDKN1C	GENE BODY	CGP-082996	-0.3327	3.24E-07	2.09E-05	11p15.4	225	GDSC
SLC22A3	EXON1	Imatinib	-0.3325	2.29E-07	1.61E-05	6q25.3	231	GDSC
PEG3	UTR5	WZ-1-84	-0.3322	3.37E-07	2.16E-05	19q13.43	225	GDSC
ZIM2	UTR5	WZ-1-84	-0.3322	3.37E-07	2.16E-05	19q13.43	225	GDSC
ZIM3	TSS1500	WZ-1-84	-0.3315	3.82E-07	2.37E-05	19q13.43	224	GDSC
IGF2	EXON1	WZ-1-84	-0.3315	3.60E-07	2.27E-05	11p15.5	225	GDSC
GABRB3	EXON1	Sunitinib	-0.3315	3.60E-07	2.27E-05	15q12	225	GDSC
DDC	GENE BODY	Docetaxel	0.3314	6.00E-15	2.30E-11	7p12.1	526	GDSC
ZIM3	UTR5	XMD8-85	-0.3311	4.74E-07	2.80E-05	19q13.43	221	GDSC
PHLDA2	UTR3	Axitinib	-0.3298	9.84E-15	3.33E-11	11p15.4	523	GDSC
PHACTR2	UTR5	XMD8-85	-0.3294	5.44E-07	3.09E-05	6q24.2	221	GDSC
PHACTR2	EXON1	XMD8-85	-0.3294	5.44E-07	3.09E-05	6q24.2	221	GDSC
CDKN1C	EXON1	PHA-665752	-0.3293	3.03E-07	1.99E-05	11p15.4	231	GDSC
ZIM3	UTR3	WZ-1-84	-0.3292	4.63E-07	2.75E-05	19q13.43	224	GDSC
PEG3	TSS1500	XMD8-85	-0.3290	5.96E-07	3.31E-05	19q13.43	220	GDSC
USP29	UTR5	XMD8-85	-0.3290	5.97E-07	3.31E-05	19q13.43	220	GDSC
LRRTM1	TSS200	WZ-1-84	-0.3290	4.45E-07	2.68E-05	2p12	225	GDSC
BLCAP	EXON1	GSK319347A	-0.3287	5.12E-07	2.97E-05	20q11.23	223	GDSC
KCNK9	TSS1500	Z-LLNle-CHO	-0.3287	4.57E-07	2.72E-05	8q24.3	225	GDSC
NTM	UTR5	Salubrinal	-0.3285	5.53E-07	3.12E-05	11q25	222	GDSC
NTM	EXON1	Salubrinal	-0.3285	5.53E-07	3.12E-05	11q25	222	GDSC
KLF14	EXON1	XMD8-85	-0.3281	6.10E-07	3.35E-05	7q32.3	221	GDSC
GABRG3	UTR5	Sunitinib	-0.3278	4.94E-07	2.89E-05	15q12	225	GDSC
GABRG3	EXON1	Sunitinib	-0.3278	4.94E-07	2.89E-05	15q12	225	GDSC
PHACTR2	UTR5	Cyclopamine	-0.3276	6.33E-07	3.45E-05	6q24.2	221	GDSC
PHACTR2	EXON1	Cyclopamine	-0.3276	6.33E-07	3.45E-05	6q24.2	221	GDSC
WIF1	UTR5	CGP-082996	-0.3269	5.32E-07	3.04E-05	12q14.3	225	GDSC
WIF1	EXON1	CGP-082996	-0.3269	5.32E-07	3.04E-05	12q14.3	225	GDSC
GABRB3	EXON1	WZ-1-84	-0.3268	5.37E-07	3.07E-05	15q12	225	GDSC
DIO3	UTR5	CGP-082996	-0.3267	5.40E-07	3.08E-05	14q32.31	225	GDSC
CDKN1C	EXON1	CMK	-0.3266	5.45E-07	3.09E-05	11p15.4	225	GDSC
ANO1	EXON1	TGX221	-0.3266	6.14E-07	3.37E-05	11q13.3	223	GDSC
PHLDA2	UTR3	QL-XI-92	-0.3264	4.08E-16	3.85E-12	11p15.4	590	GDSC
PHLDA2	TSS1500	ZG-10	-0.3263	9.59E-09	1.43E-06	11p15.4	295	GDSC
MIMT1	GENE BODY	XMD8-85	-0.3262	7.09E-07	3.77E-05	19q13.43	221	GDSC
SNRPN	TSS200	WZ-1-84	-0.3262	5.66E-07	3.18E-05	15q11.2	225	GDSC
CALCR	TSS200	Z-LLNle-CHO	-0.3261	5.67E-07	3.19E-05	7q21.3	225	GDSC
BLCAP	EXON1	GSK429286A	-0.3256	4.92E-16	4.36E-12	20q11.23	590	GDSC
CDKN1C	TSS200	Cyclopamine	-0.3256	7.51E-07	3.94E-05	11p15.4	221	GDSC
GABRB3	EXON1	XMD8-85	-0.3254	7.58E-07	3.97E-05	15q12	221	GDSC
PHLDA2	TSS200	CMK	-0.3254	6.01E-07	3.33E-05	11p15.4	225	GDSC
KCNK9	EXON1	Z-LLNle-CHO	-0.3252	6.14E-07	3.37E-05	8q24.3	225	GDSC
PHLDA2	UTR3	Daporinad	-0.3251	1.79E-15	9.73E-12	11p15.4	569	GDSC
ZIM2	TSS1500	XMD8-85	-0.3250	8.33E-07	4.26E-05	19q13.43	220	GDSC
DIO3	TSS200	Z-LLNle-CHO	-0.3249	6.30E-07	3.43E-05	14q32.31	225	GDSC
GABRB3	EXON1	CGP-082996	-0.3248	6.32E-07	3.44E-05	15q12	225	GDSC
PHLDA2	TSS200	Cyclopamine	-0.3248	7.99E-07	4.14E-05	11p15.4	221	GDSC
BLCAP	EXON1	XMD8-85	-0.3246	8.10E-07	4.19E-05	20q11.23	221	GDSC
GABRG3	TSS200	XMD8-85	-0.3244	8.26E-07	4.24E-05	15q12	221	GDSC
PEG3	EXON1	WZ-1-84	-0.3242	6.68E-07	3.60E-05	19q13.43	225	GDSC
LRRTM1	UTR5	WZ-1-84	-0.3241	6.72E-07	3.62E-05	2p12	225	GDSC
RASGRF1	TSS200	Z-LLNle-CHO	-0.3241	6.73E-07	3.62E-05	15q25.1	225	GDSC
SLC22A18AS	TSS1500	TL-1-85	-0.3241	6.43E-16	5.06E-12	11p15.4	591	GDSC
SLC22A3	EXON1	JW-7-24-1	-0.3238	7.15E-16	5.33E-12	6q25.3	590	GDSC
BLCAP	EXON1	Crizotinib	-0.3238	5.17E-07	2.98E-05	20q11.23	230	GDSC
LRRTM1	TSS1500	WZ-1-84	-0.3237	6.95E-07	3.71E-05	2p12	225	GDSC
PPP1R9A	UTR5	Methotrexate	-0.3236	2.88E-14	7.42E-11	7q21.3	525	GDSC
PHLDA2	UTR3	Masitinib	-0.3234	8.29E-16	5.44E-12	11p15.4	589	GDSC
KLF14	EXON1	WZ-1-84	-0.3232	7.24E-07	3.83E-05	7q32.3	225	GDSC
PHLDA2	UTR3	Quizartinib	-0.3231	8.84E-16	5.44E-12	11p15.4	589	GDSC
PHLDA2	TSS200	GSK319347A	-0.3230	8.23E-07	4.23E-05	11p15.4	223	GDSC
DIO3	TSS1500	XMD8-85	-0.3230	9.29E-07	4.65E-05	14q32.31	221	GDSC
CALCR	TSS1500	XMD8-85	-0.3222	9.89E-07	4.89E-05	7q21.3	221	GDSC
SLC22A18AS	GENE BODY	Selumetinib	0.3220	5.79E-16	4.83E-12	11p15.4	601	GDSC
PHLDA2	TSS200	Crizotinib	-0.3219	6.10E-07	3.35E-05	11p15.4	230	GDSC
NDN	EXON1	WZ-1-84	-0.3218	8.15E-07	4.21E-05	15q11.2	225	GDSC
GABRG3	TSS1500	WZ-1-84	-0.3217	8.20E-07	4.22E-05	15q12	225	GDSC
PHLDA2	UTR3	KIN001-270	-0.3215	1.13E-15	6.66E-12	11p15.4	591	GDSC
ANO1	EXON1	Z-LLNle-CHO	-0.3214	8.43E-07	4.30E-05	11q13.3	225	GDSC
SLC22A3	TSS200	GSK319347A	-0.3211	9.63E-07	4.78E-05	6q25.3	223	GDSC
USP29	TSS1500	XMD8-85	-0.3210	1.09E-06	5.26E-05	19q13.43	221	GDSC
GABRG3	TSS200	Z-LLNle-CHO	-0.3208	8.88E-07	4.46E-05	15q12	225	GDSC
AIM1	GENE BODY	Crizotinib	0.3203	6.94E-07	3.71E-05	6q21	230	GDSC
DIO3	TSS200	Sunitinib	-0.3202	9.30E-07	4.65E-05	14q32.31	225	GDSC
PHLDA2	UTR3	TL-1-85	-0.3202	1.49E-15	8.42E-12	11p15.4	591	GDSC
PHLDA2	UTR3	GSK1070916	-0.3201	3.47E-15	1.58E-11	11p15.4	576	GDSC
RB1	TSS1500	Salubrinal	-0.3198	1.20E-06	5.68E-05	13q14.2	221	GDSC
CPA4	UTR5	TL-2-105	-0.3191	1.96E-15	9.91E-12	7q32.2	590	GDSC
CPA4	EXON1	TL-2-105	-0.3191	1.96E-15	9.91E-12	7q32.2	590	GDSC
USP29	GENE BODY	XMD8-85	-0.3185	1.41E-06	6.40E-05	19q13.43	220	GDSC

PEG3	UTR5	Salubrinal	-0.3180	1.32E-06	6.07E-05	19q13.43	222	GDSC
ZIM2	UTR5	Salubrinal	-0.3180	1.32E-06	6.07E-05	19q13.43	222	GDSC
SLC22A3	EXON1	Tubastatin A	-0.3177	3.10E-15	1.50E-11	6q25.3	587	GDSC
BLCAP	EXON1	STF-62247	-0.3176	3.17E-15	1.50E-11	20q11.23	587	GDSC
MAGI2	UTR5	Lapatinib	-0.3174	1.54E-06	6.82E-05	7q21.11	220	GDSC
SLC22A3	EXON1	PI-103	-0.3171	3.95E-15	1.75E-11	6q25.3	585	GDSC
IGF2	EXON1	Lapatinib	-0.3171	1.58E-06	6.96E-05	11p15.5	220	GDSC
CALCR	TSS200	Sunitinib	-0.3171	1.20E-06	5.68E-05	7q21.3	225	GDSC
CDKN1C	GENE BODY	Cyclopamine	-0.3170	1.50E-06	6.68E-05	11p15.4	221	GDSC
DDC	UTR3	Docetaxel	0.3159	1.18E-13	2.43E-10	7p12.1	526	GDSC
PHLDA2	UTR3	Tanespimycin	0.3159	1.12E-13	2.34E-10	11p15.4	527	GDSC
PHLDA2	UTR5	GSK319347A	-0.3159	1.48E-06	6.60E-05	11p15.4	223	GDSC
SLC22A18AS	TSS1500	Cyclopamine	-0.3158	1.65E-06	7.20E-05	11p15.4	221	GDSC
PEG3	UTR5	XMD8-85	-0.3158	1.66E-06	7.22E-05	19q13.43	221	GDSC
ZIM2	UTR5	XMD8-85	-0.3158	1.66E-06	7.22E-05	19q13.43	221	GDSC
ANO1	EXON1	CGP-082996	-0.3158	1.33E-06	6.14E-05	11q13.3	225	GDSC
GABRG3	TSS200	Sunitinib	-0.3157	1.34E-06	6.15E-05	15q12	225	GDSC
PHLDA2	TSS200	Z-LLNle-CHO	-0.3157	1.35E-06	6.17E-05	11p15.4	225	GDSC
NNAT	GENE BODY	GSK319347A	-0.3151	1.57E-06	6.93E-05	20q11.23	223	GDSC
DIO3	TSS200	GNF-2	-0.3150	1.43E-06	6.44E-05	14q32.31	225	GDSC
AIM1	GENE BODY	XMD8-85	0.3148	1.79E-06	7.64E-05	6q21	221	GDSC
PHLDA2	UTR3	NG-25	-0.3148	4.63E-15	1.99E-11	11p15.4	591	GDSC
CPA4	UTR5	Quizartinib	-0.3147	5.23E-15	2.11E-11	7q32.2	589	GDSC
CPA4	EXON1	Quizartinib	-0.3147	5.23E-15	2.11E-11	7q32.2	589	GDSC
AIM1	GENE BODY	Parthenolide	0.3141	1.80E-06	7.65E-05	6q21	222	GDSC
CDKN1C	EXON1	CGP-60474	-0.3139	1.55E-06	6.84E-05	11p15.4	225	GDSC
SLC22A3	EXON1	KIN001-260	-0.3139	5.63E-15	2.21E-11	6q25.3	591	GDSC
GABRB3	UTR5	Sunitinib	-0.3133	1.63E-06	7.12E-05	15q12	225	GDSC
ZIM3	UTR3	Salubrinal	-0.3133	2.02E-06	8.37E-05	19q13.43	221	GDSC
ZIM3	UTR3	GSK319347A	-0.3132	1.94E-06	8.10E-05	19q13.43	222	GDSC
DDC	TSS1500	Dactolisib	0.3131	2.47E-13	4.54E-10	7p12.1	522	GDSC
PPP1R9A	EXON1	CGP-082996	-0.3130	1.67E-06	7.25E-05	7q21.3	225	GDSC
SLC22A3	TSS200	JW-7-24-1	-0.3127	7.50E-15	2.72E-11	6q25.3	590	GDSC
BLCAP	EXON1	TL-1-85	-0.3127	7.12E-15	2.65E-11	20q11.23	591	GDSC
NTM	UTR3	Topotecan	-0.3127	6.65E-10	1.99E-07	11q25	373	CCLE
PHLDA2	UTR3	PI-103	-0.3125	1.01E-14	3.33E-11	11p15.4	585	GDSC
PPP1R9A	UTR5	PHA-665752	-0.3121	1.31E-06	6.04E-05	7q21.3	231	GDSC
SLC22A3	EXON1	OSI-930	-0.3118	9.56E-15	3.33E-11	6q25.3	589	GDSC
DIO3	TSS200	Crizotinib	-0.3116	1.43E-06	6.45E-05	14q32.31	230	GDSC
PWRN1	TSS1500	Salubrinal	-0.3115	2.22E-06	8.98E-05	15q11.2	222	GDSC
PHLDA2	TSS1500	GNF-2	-0.3113	1.91E-06	8.00E-05	11p15.4	225	GDSC
INPP5F	GENE BODY	PI-103	-0.3111	1.36E-14	4.39E-11	10q26.11	585	GDSC
SLC22A3	TSS200	KIN001-270	-0.3111	1.00E-14	3.33E-11	6q25.3	591	GDSC
NTM	GENE BODY	Topotecan	-0.3109	8.44E-10	2.35E-07	11q25	373	CCLE
CDKN1C	EXON1	Crizotinib	-0.3101	1.61E-06	7.07E-05	11p15.4	230	GDSC
PEG3	TSS200	WZ-1-84	-0.3098	2.16E-06	8.79E-05	19q13.43	225	GDSC
ZIM2	TSS200	WZ-1-84	-0.3098	2.16E-06	8.79E-05	19q13.43	225	GDSC
CALCR	TSS200	CGP-082996	-0.3096	2.19E-06	8.90E-05	7q21.3	225	GDSC
PHLDA2	UTR3	OSI-027	-0.3096	1.58E-14	4.87E-11	11p15.4	588	GDSC
DLX5	TSS200	Cyclopamine	-0.3094	2.74E-06	0.0001054	7q21.3	221	GDSC
GABRG3	UTR5	XMD8-85	-0.3093	2.76E-06	0.00010594	15q12	221	GDSC
GABRG3	EXON1	XMD8-85	-0.3093	2.76E-06	0.00010594	15q12	221	GDSC
AIM1	GENE BODY	PD-0332991	0.3093	2.07E-08	2.59E-06	6q21	315	CCLE
DLX5	UTR5	Cyclopamine	-0.3093	2.76E-06	0.00010614	7q21.3	221	GDSC
PHLDA2	TSS1500	BX-912	-0.3093	1.52E-14	4.79E-11	11p15.4	590	GDSC
SNORD107	TSS1500	XMD8-85	-0.3092	2.78E-06	0.00010649	15q11.2	221	GDSC
DIO3	TSS200	CGP-082996	-0.3091	2.27E-06	9.17E-05	14q32.31	225	GDSC
IGF2R	EXON1	CGP-082996	-0.3089	2.32E-06	9.30E-05	6q25.3	225	GDSC
SLC22A3	EXON1	I-BET-762	-0.3088	1.97E-14	5.94E-11	6q25.3	587	GDSC
GABRG3	TSS1500	XMD8-85	-0.3085	2.96E-06	0.0001117	15q12	221	GDSC
USP29	GENE BODY	Salubrinal	-0.3083	2.98E-06	0.00011226	19q13.43	221	GDSC
MIMT1	GENE BODY	WZ-1-84	-0.3082	2.45E-06	9.71E-05	19q13.43	225	GDSC
ZIM3	UTR3	GNF-2	-0.3079	2.64E-06	0.00010273	19q13.43	224	GDSC
SLC22A3	EXON1	GSK690693	-0.3077	2.34E-14	6.76E-11	6q25.3	588	GDSC
ZIM3	TSS1500	XMD8-85	-0.3077	3.31E-06	0.00012109	19q13.43	220	GDSC
SLC22A3	TSS200	Zibotentan	-0.3074	2.12E-14	6.26E-11	6q25.3	591	GDSC
SLC22A3	TSS200	T0901317	-0.3073	2.77E-14	7.40E-11	6q25.3	586	GDSC
C15orf2	UTR5	XMD8-85	-0.3069	3.33E-06	0.00012164	15q11.2	221	GDSC
GABRG3	TSS200	CGP-082996	-0.3069	2.71E-06	0.0001047	15q12	225	GDSC
CDKN1C	EXON1	Sunitinib	-0.3068	2.73E-06	0.00010531	11p15.4	225	GDSC
PHLDA2	UTR3	Tivozanib	-0.3067	2.72E-14	7.40E-11	11p15.4	589	GDSC
PHACTR2	TSS200	CGP-082996	-0.3065	2.80E-06	0.00010719	6q24.2	225	GDSC
AIM1	GENE BODY	Vismodegib	0.3065	6.70E-13	9.68E-10	6q21	526	GDSC
PLAGL1	UTR3	Sunitinib	-0.3064	2.82E-06	0.00010765	6q24.2	225	GDSC
PHLDA2	TSS1500	TL-1-85	-0.3063	2.65E-14	7.36E-11	11p15.4	591	GDSC
BLCAP	EXON1	JW-7-24-1	-0.3062	2.86E-14	7.42E-11	20q11.23	590	GDSC
PEG10	UTR3	Crizotinib	0.3062	2.22E-06	9.00E-05	7q21.3	230	GDSC
DLX5	TSS200	Z-LLNle-CHO	-0.3060	2.91E-06	0.00011058	7q21.3	225	GDSC
DLX5	UTR5	Z-LLNle-CHO	-0.3060	2.92E-06	0.00011085	7q21.3	225	GDSC
PPP1R9A	UTR5	Lenalidomide	-0.3059	7.45E-13	1.06E-09	7q21.3	526	GDSC
DIO3	UTR5	Z-LLNle-CHO	-0.3058	2.94E-06	0.00011148	14q32.31	225	GDSC
IGF2	GENE BODY	WZ-1-84	-0.3058	2.96E-06	0.0001118	11p15.5	225	GDSC
INS-IGF2	GENE BODY	WZ-1-84	-0.3058	2.97E-06	0.00011188	11p15.5	225	GDSC

INS-IGF2	GENE BODY	WZ-1-84	-0.3058	2.97E-06	0.00011188	11p15.5	225	GDSC
IGF2	GENE BODY	CGP-082996	-0.3056	3.00E-06	0.0001127	11p15.5	225	GDSC
ZFAT	UTR5	Dasatinib	0.3055	3.34E-06	0.00012187	8q24.22	223	GDSC
CDKN1C	UTR5	Z-LLNle-CHO	-0.3049	3.18E-06	0.0001175	11p15.4	225	GDSC
PEG3	TSS200	PHA-665752	-0.3048	2.35E-06	9.43E-05	19q13.43	231	GDSC
ZIM2	TSS200	PHA-665752	-0.3048	2.35E-06	9.43E-05	19q13.43	231	GDSC
KCNK9	TSS1500	CGP-082996	-0.3045	3.27E-06	0.00012033	8q24.3	225	GDSC
SLC22A3	TSS200	PI-103	-0.3042	5.43E-14	1.33E-10	6q25.3	585	GDSC
DLX5	TSS200	Salubrinal	-0.3041	3.94E-06	0.0001378	7q21.3	222	GDSC
SLC22A18	TSS1500	Selumetinib	0.3040	2.57E-14	7.28E-11	11p15.4	601	GDSC
GABRB3	UTR5	XMD8-85	-0.3039	4.20E-06	0.00014457	15q12	221	GDSC
DDC	TSS1500	PLX-4720	0.3037	3.01E-14	7.62E-11	7p12.1	599	GDSC
SLC22A18AS	TSS1500	PI-103	-0.3036	6.15E-14	1.45E-10	11p15.4	585	GDSC
SLC22A3	TSS200	Temozolomide	-0.3032	1.20E-13	2.43E-10	6q25.3	573	GDSC
GABRB3	UTR5	CGP-082996	-0.3031	3.64E-06	0.00013002	15q12	225	GDSC
IGF2AS	TSS1500	Z-LLNle-CHO	-0.3031	3.65E-06	0.00013026	11p15.5	225	GDSC
GLIS3	EXON1	Imatinib	-0.3030	2.71E-06	0.0001047	9p24.2	231	GDSC
CALCR	TSS1500	CGP-082996	-0.3030	3.67E-06	0.00013078	7q21.3	225	GDSC
DLK1	EXON1	WZ-1-84	-0.3030	3.68E-06	0.00013092	14q32.2	225	GDSC
PPP1R9A	EXON1	WZ-1-84	-0.3030	3.68E-06	0.00013096	7q21.3	225	GDSC
SLC22A3	TSS200	GSK690693	-0.3029	6.03E-14	1.45E-10	6q25.3	588	GDSC
MAGEL2	EXON1	XMD8-85	-0.3029	4.52E-06	0.00015214	15q11.2	221	GDSC
CALCR	TSS200	Cyclopamine	-0.3027	4.58E-06	0.00015356	7q21.3	221	GDSC
ZIM3	UTR3	Cyclopamine	-0.3027	4.82E-06	0.00015932	19q13.43	220	GDSC
ZIM2	TSS1500	WZ-1-84	-0.3027	3.95E-06	0.00013806	19q13.43	224	GDSC
SLC22A3	EXON1	OSI-027	-0.3026	6.46E-14	1.48E-10	6q25.3	588	GDSC
GABRG3	UTR5	CGP-082996	-0.3025	3.81E-06	0.00013422	15q12	225	GDSC
GABRG3	EXON1	CGP-082996	-0.3025	3.81E-06	0.00013422	15q12	225	GDSC
PEG3	TSS1500	WZ-1-84	-0.3025	4.03E-06	0.00014013	19q13.43	224	GDSC
PPP1R9A	TSS1500	XMD8-85	-0.3024	4.70E-06	0.00015632	7q21.3	221	GDSC
ZIM3	GENE BODY	WZ-1-84	-0.3024	4.04E-06	0.00014044	19q13.43	224	GDSC
SLC22A18AS	TSS1500	GSK429286A	-0.3022	6.35E-14	1.47E-10	11p15.4	590	GDSC
CDKN1C	EXON1	Salubrinal	-0.3021	4.56E-06	0.00015304	11p15.4	222	GDSC
PPP1R9A	UTR5	GNF-2	-0.3019	4.01E-06	0.00013957	7q21.3	225	GDSC
SNRPN	UTR5	XMD8-85	-0.3018	4.92E-06	0.00016173	15q11.2	221	GDSC
GRB10	EXON1	WZ-1-84	-0.3017	4.05E-06	0.00014059	7p12.1	225	GDSC
GNAS	GENE BODY	WH-4-023	-0.3017	4.48E-06	0.00015115	20q13.32	223	GDSC
NNAT	GENE BODY	Salubrinal	-0.3017	4.72E-06	0.00015685	20q11.23	222	GDSC
PHLDA2	UTR3	TL-2-105	-0.3017	7.06E-14	1.56E-10	11p15.4	590	GDSC
KCNK9	UTR5	Z-LLNle-CHO	-0.3016	4.08E-06	0.00014139	8q24.3	225	GDSC
SLC22A3	EXON1	T0901317	-0.3016	8.62E-14	1.85E-10	6q25.3	586	GDSC
GABRB3	UTR5	WZ-1-84	-0.3015	4.12E-06	0.00014252	15q12	225	GDSC
DIO3	UTR5	GNF-2	-0.3012	4.22E-06	0.00014497	14q32.31	225	GDSC
PHLDA2	EXON1	GSK319347A	-0.3009	4.77E-06	0.00015806	11p15.4	223	GDSC
DLGAP2	TSS200	PF2341066	-0.3009	3.52E-09	6.85E-07	8p23.3	370	CCLE
RNU5D	GENE BODY	ZG-10	-0.3007	1.40E-07	1.12E-05	5q14.1	295	GDSC
CDKN1C	UTR5	XMD8-85	-0.3006	5.40E-06	0.00017349	11p15.4	221	GDSC
PHLDA2	UTR3	BIX02189	-0.3005	8.54E-14	1.85E-10	11p15.4	591	GDSC
SLC22A3	TSS200	Imatinib	-0.3004	3.34E-06	0.00012183	6q25.3	231	GDSC
PHLDA2	EXON1	Cyclopamine	-0.3004	5.49E-06	0.00017573	11p15.4	221	GDSC
CALCR	TSS1500	WZ-1-84	-0.3003	4.53E-06	0.00015222	7q21.3	225	GDSC
AIM1	GENE BODY	Cyclopamine	0.3003	5.53E-06	0.00017657	6q21	221	GDSC
GLIS3	GENE BODY	Crizotinib	0.3002	3.57E-06	0.00012823	9p24.2	230	GDSC
PLAGL1	UTR3	Z-LLNle-CHO	-0.3001	4.60E-06	0.00015394	6q24.2	225	GDSC

Correlation results for the regions of the genes located on 20q11-q13.3 are highlighted in yellow.

Spearman ρ, Spearman correlation coefficient**.** The results are sorted by the absolute value of |ρ|.
***p* $_0$,** *p*- value prior to FDR adjustment
***p* $_{FDR}$,** *p* -value after FDR adjustment

Cytoband, chromosomal region location according to the UCSC genome annotation database for the hg19 (GRCh37) assembly of the human genome based on the probe coordinates in the Illumina Infinium HumanMethylation 450K BeadChip annotation

Sample size, number of cell lines with available data used in correlation analysis
Drug response data source, dataset (GDSC or CCLE) from which the drug response values were obtained

The 6 gene regions include **TSS1500**, **TSS200**, 5'UTR (**UTR5**), 1st exon (**EXON1**), gene body (**GENE BODY**), and 3' UTR (**UTR3**).
