# Supplementary material for: Increased copy number of imprinted genes in the chromosomal region 20q11-q13.32 is associated with resistance to antitumor agents in cancer cell lines
Source: Clin Epigenetics. 2022 Dec 2;14:161. doi: 10.1186/s13148-022-01368-7 (PMC9716673; doi:10.1186/s13148-022-01368-7)
Supplement: Supplementary file 10 — Additional file 10: Table S7. Spearman and Pearson correlation among copy number values of imprinted genes in the chromosomal region 20q11-q13 and non-imprinted cancer genes AURKA, MYBL2, and ZNF217. Shown are associations of copy number of non-imprinted and imprinted genes with copy number values of imprinted genes at 20q11-q13 listed in Table 1 whose copy number values were associated with drug response (pSegmFDR < 0.05 and Spearman |ρ| > 0.3). ρ, Spearman correlation coefficient; r, Pearson correlation coefficient. [file 13148_2022_1368_MOESM10_ESM.pdf]

Table S7. Spearman and Pearson correlation among copy number values of non-imprinted cancer genes *AURKA, MYBL2,* and Z*NF217* and imprinted genes in the chromosomal region 20q11-q13

Gene 1	Gene 2	Imprinting status of gene 1	Spearman ρ_o	Spearman p	Pearson r	Pearson r_o
AURKA	***GNAS-AS1***	Not imprinted	0.9659	0	0.9553	0
AURKA	***MIR296***	Not imprinted	0.9659	0	0.9553	0
AURKA	***MIR298***	Not imprinted	0.9659	0	0.9553	0
AURKA	***GNAS***	Not imprinted	0.9642	0	0.9414	2.85E-295
AURKA	***ZNF217***	Not imprinted	0.9481	2.946E-311	0.9046	3.65E-232
AURKA	***MYBL2***	Not imprinted	0.8004	4.12E-140	0.6683	7.43E-82
AURKA	***BLCAP***	Not imprinted	0.7655	4.88E-121	0.6266	2.99E-69
AURKA	***NNAT***	Not imprinted	0.7655	4.88E-121	0.6266	2.99E-69
AURKA	***HM13***	Not imprinted	0.6257	5.27E-69	0.4382	1.29E-30
MYBL2	***BLCAP***	Not imprinted	0.8919	3.59E-216	0.8255	2.18E-156
MYBL2	***NNAT***	Not imprinted	0.8919	3.59E-216	0.8255	2.18E-156
MYBL2	***ZNF217***	Not imprinted	0.8006	3.07E-140	0.6424	8.80E-74
MYBL2	***AURKA***	Not imprinted	0.8004	4.12E-140	0.6683	7.43E-82
MYBL2	***GNAS***	Not imprinted	0.7839	1.23E-130	0.692	5.90E-90
MYBL2	***GNAS-AS1***	Not imprinted	0.7782	1.49E-127	0.6471	3.42E-75
MYBL2	***MIR296***	Not imprinted	0.7782	1.49E-127	0.6471	3.42E-75
MYBL2	***MIR298***	Not imprinted	0.7782	1.49E-127	0.6471	3.42E-75
MYBL2	***HM13***	Not imprinted	0.6952	4.25E-91	0.5311	1.26E-46
ZNF217	***AURKA***	Not imprinted	0.9481	2.946E-311	0.9046	3.65E-232
ZNF217	***GNAS-AS1***	Not imprinted	0.9288	5.36E-270	0.8907	8.01E-215
ZNF217	***MIR296***	Not imprinted	0.9288	5.36E-270	0.8907	8.01E-215
ZNF217	***MIR298***	Not imprinted	0.9288	5.36E-270	0.8907	8.01E-215
ZNF217	***GNAS***	Not imprinted	0.9271	8.63E-267	0.8632	1.87E-186
ZNF217	***MYBL2***	Not imprinted	0.8006	3.07E-140	0.6424	8.80E-74
ZNF217	***BLCAP***	Not imprinted	0.7605	1.53E-118	0.5599	1.06E-52
ZNF217	***NNAT***	Not imprinted	0.7605	1.53E-118	0.5599	1.06E-52
ZNF217	***HM13***	Not imprinted	0.6286	7.93E-70	0.4606	4.85E-34
BLCAP	***NNAT***	Imprinted	1	0	1	0
BLCAP	***MYBL2***	Imprinted	0.8919	3.59E-216	0.8255	2.18E-156
BLCAP	***AURKA***	Imprinted	0.7655	4.88E-121	0.6266	2.99E-69
BLCAP	***ZNF217***	Imprinted	0.7605	1.53E-118	0.5599	1.06E-52
BLCAP	***GNAS***	Imprinted	0.7562	1.83E-116	0.6342	2.12E-71

BLCAP	*GNAS-AS1*	Imprinted	0.7505	8.55E-114	0.5888	2.09E-59
BLCAP	*MIR296*	Imprinted	0.7505	8.55E-114	0.5888	2.09E-59
BLCAP	*MIR298*	Imprinted	0.7505	8.55E-114	0.5888	2.09E-59
BLCAP	*HM13*	Imprinted	0.7314	2.41E-105	0.5748	4.53E-56
GNAS	*GNAS*	Imprinted	1	0	1	0
GNAS	*GNAS-AS1*	Imprinted	0.9984	0	0.9837	0
GNAS	*MIR296*	Imprinted	0.9984	0	0.9837	0
GNAS	*MIR298*	Imprinted	0.9984	0	0.9837	0
GNAS	*AURKA*	Imprinted	0.9642	0	0.9414	2.85E-295
GNAS	*ZNF217*	Imprinted	0.9271	8.63E-267	0.8632	1.87E-186
GNAS	*MYBL2*	Imprinted	0.7839	1.23E-130	0.692	5.90E-90
GNAS	*BLCAP*	Imprinted	0.7562	1.83E-116	0.6342	2.12E-71
GNAS	*NNAT*	Imprinted	0.7562	1.83E-116	0.6342	2.12E-71
GNAS	*HM13*	Imprinted	0.6149	4.63E-66	0.4377	1.53E-30
GNAS-AS1	*MIR296*	Imprinted	1	0	1	0
GNAS-AS1	*MIR298*	Imprinted	1	0	1	0
GNAS-AS1	*GNAS*	Imprinted	0.9984	0	0.9837	0
GNAS-AS1	*AURKA*	Imprinted	0.9659	0	0.9553	0
GNAS-AS1	*ZNF217*	Imprinted	0.9288	5.36E-270	0.8907	8.01E-215
GNAS-AS1	*MYBL2*	Imprinted	0.7782	1.49E-127	0.6471	3.42E-75
GNAS-AS1	*BLCAP*	Imprinted	0.7505	8.55E-114	0.5888	2.09E-59
GNAS-AS1	*NNAT*	Imprinted	0.7505	8.55E-114	0.5888	2.09E-59
GNAS-AS1	*HM13*	Imprinted	0.6162	2.09E-66	0.4375	1.60E-30
HM13	*BLCAP*	Imprinted	0.7314	2.41E-105	0.5748	4.53E-56
HM13	*NNAT*	Imprinted	0.7314	2.41E-105	0.5748	4.53E-56
HM13	*MYBL2*	Imprinted	0.6952	4.25E-91	0.5311	1.26E-46
HM13	*ZNF217*	Imprinted	0.6286	7.93E-70	0.4606	4.85E-34
HM13	*AURKA*	Imprinted	0.6257	5.27E-69	0.4382	1.29E-30
HM13	*GNAS-AS1*	Imprinted	0.6162	2.09E-66	0.4375	1.60E-30
HM13	*MIR296*	Imprinted	0.6162	2.09E-66	0.4375	1.60E-30
HM13	*MIR298*	Imprinted	0.6162	2.09E-66	0.4375	1.60E-30
HM13	*GNAS*	Imprinted	0.6149	4.63E-66	0.4377	1.53E-30
MIR296	*GNAS-AS1*	Imprinted	1	0	1	0
MIR296	*MIR298*	Imprinted	1	0	1	0
MIR296	*GNAS*	Imprinted	0.9984	0	0.9837	0
MIR296	*AURKA*	Imprinted	0.9659	0	0.9553	0

MIR296	***ZNF217***	Imprinted	0.9288	5.36E-270	0.8907	8.01E-215
MIR296	***MYBL2***	Imprinted	0.7782	1.49E-127	0.6471	3.42E-75
MIR296	***BLCAP***	Imprinted	0.7505	8.55E-114	0.5888	2.09E-59
MIR296	***NNAT***	Imprinted	0.7505	8.55E-114	0.5888	2.09E-59
MIR296	***HM13***	Imprinted	0.6162	2.09E-66	0.4375	1.60E-30
MIR298	***GNAS-AS1***	Imprinted	1	0	1	0
MIR298	***MIR296***	Imprinted	1	0	1	0
MIR298	***GNAS***	Imprinted	0.9984	0	0.9837	0
MIR298	***AURKA***	Imprinted	0.9659	0	0.9553	0
MIR298	***ZNF217***	Imprinted	0.9288	5.36E-270	0.8907	8.01E-215
MIR298	***MYBL2***	Imprinted	0.7782	1.49E-127	0.6471	3.42E-75
MIR298	***BLCAP***	Imprinted	0.7505	8.55E-114	0.5888	2.09E-59
MIR298	***NNAT***	Imprinted	0.7505	8.55E-114	0.5888	2.09E-59
MIR298	***HM13***	Imprinted	0.6162	2.09E-66	0.4375	1.60E-30
NNAT	***BLCAP***	Imprinted	1	0	1	0
NNAT	***MYBL2***	Imprinted	0.8919	3.59E-216	0.8255	2.18E-156
NNAT	***AURKA***	Imprinted	0.7655	4.88E-121	0.6266	2.99E-69
NNAT	***ZNF217***	Imprinted	0.7605	1.53E-118	0.5599	1.06E-52
NNAT	***GNAS***	Imprinted	0.7562	1.83E-116	0.6342	2.12E-71
NNAT	***GNAS-AS1***	Imprinted	0.7505	8.55E-114	0.5888	2.09E-59
NNAT	***MIR296***	Imprinted	0.7505	8.55E-114	0.5888	2.09E-59
NNAT	***MIR298***	Imprinted	0.7505	8.55E-114	0.5888	2.09E-59
NNAT	***HM13***	Imprinted	0.7314	2.41E-105	0.5748	4.53E-56

Shown are associations of copy number of non-imprinted and imprinted genes with copy number values of imprinted genes on 20q11-q13 listed in Table 1 whose copy number was associated with drug response ($p_{SegmFDR} < 0.05$ and Spearman $|\rho| > 0.3$)

ρ, Spearman correlation coefficient
r, Pearson correlation coefficient
