# Supplementary material for: Increased copy number of imprinted genes in the chromosomal region 20q11-q13.32 is associated with resistance to antitumor agents in cancer cell lines
Source: Clin Epigenetics. 2022 Dec 2;14:161. doi: 10.1186/s13148-022-01368-7 (PMC9716673; doi:10.1186/s13148-022-01368-7)
Supplement: Supplementary file 11 — Additional file 11: Table S8. Correlations of expression of imprinted and non-imprinted genes at 20q11-q13.32 with log(IC50) satisfying Spearman |ρ| > 0.25.All correlations satisfying |ρ| > 0.25 had pFDR < 0.1. An asterisk (*) shows correlations satisfying more stringent criteria of Spearman |ρ| > 0.3 and pFDR < 0.1. Correlations are highlighted in shades of pink or blue according to the direction of association (positive or negative, respectively). Sample size, number of cell lines with available data used in correlation analysis. Spearman ρ, Spearman correlation coefficient. p0, p value prior to FDR adjustment. pFDR, p value after FDR adjustment. [file 13148_2022_1368_MOESM11_ESM.pdf]

Table S8. Correlations of expression of imprinted and non-imprinted genes at 20q11-q13.32 with log(IC50) satisfying Spearman |ρ| > 0.25

Gene	Agent	Spearman ρ	p_0	p_{FDR}	|ρ| > 0.3 and p_{FDR} < 0.05	Sample size	Imprinted or non-imprinted gene
SGK2	Nilotinib	0.3343	2.40E-09	2.80E-07	*	303	Imprinted
L3MBTL1	Nilotinib	0.3067	5.05E-08	2.96E-06	*	303	Imprinted
NNAT	TG101348 (fedratinib)	0.3005	0.00578	0.04507	*	83	Imprinted
GNAS	TG101348 (fedratinib)	0.2777	0.01103	0.06454		83	Imprinted
GNAS	Lestaurtinib (CEP-701)	0.2747	0.01366	0.06948		80	Imprinted
SGK2	Panobinostat	0.2705	0.00202	0.02361		128	Imprinted
GNAS	Nilotinib	0.2669	2.45E-06	9.54E-05		303	Imprinted
ZNF217	Lenalidomide	0.2606	0.00883	0.06454		100	Non-imprinted
GNAS	Lenalidomide	-0.3197	0.00118	0.01540	*	100	Imprinted
SGK2	Lenalidomide	-0.3208	0.00114	0.01540	*	100	Imprinted

All correlations satisfying |ρ| > 0.25 had p_{FDR} < 0.1.

An asterisk (*) shows correlations satisfying more stringent criteria of Spearman |ρ| > 0.3 and p_{FDR} < 0.1.

Correlations are highlighted in shades of pink or blue according to the direction of association (positive or negative, respectively).

Sample size, number of cell lines with available data used in correlation analysis

Spearman ρ, Spearman correlation coefficient

p_0, *p*- value prior to FDR adjustment

p_{FDR}, *p* -value after FDR adjustment
