# Supplementary material for: Increased copy number of imprinted genes in the chromosomal region 20q11-q13.32 is associated with resistance to antitumor agents in cancer cell lines
Source: Clin Epigenetics. 2022 Dec 2;14:161. doi: 10.1186/s13148-022-01368-7 (PMC9716673; doi:10.1186/s13148-022-01368-7)
Supplement: Supplementary file 12 — Additional file 12: Fig. S4. Scatterplots of the ex vivo log(IC50) drug response measures from the Beat AML 1.0 cohort vs log2 RPKM expression of the genes at 20q11-q13.32 for the correlations listed in Additional file 11:Table S8, which satisfy Spearman |ρ| > 0.25 and pFDR < 0.1. Shown are the following gene–drug pairs: A. SGK2 - nilotinib. B. L3MBTL1 - nilotinib. C. NNAT - TG101348 (fedratinib). D. GNAS - TG101348 (fedratinib). E. GNAS - lestaurtinib (CEP-701). F. SGK2 - panobinostat. G. GNAS - nilotinib. H. ZNF217 - lenalidomide. I. GNAS – lenalidomide. J. SGK2- lenalidomide. Samples carrying ASXL1 mutations are shown in red and are listed as ASXL1 mutation in the figure legend. Samples with a reported cytogenetic loss of all or a part of the 20q11-q13.32 region are shown as diamonds with black borders and are listed as 20q deletion in the figure legend. Spearman ρ, Spearman correlation coefficient; Pearson r, Pearson correlation coefficient. The dashed line indicates the linear regression line. [file 13148_2022_1368_MOESM12_ESM.pdf]

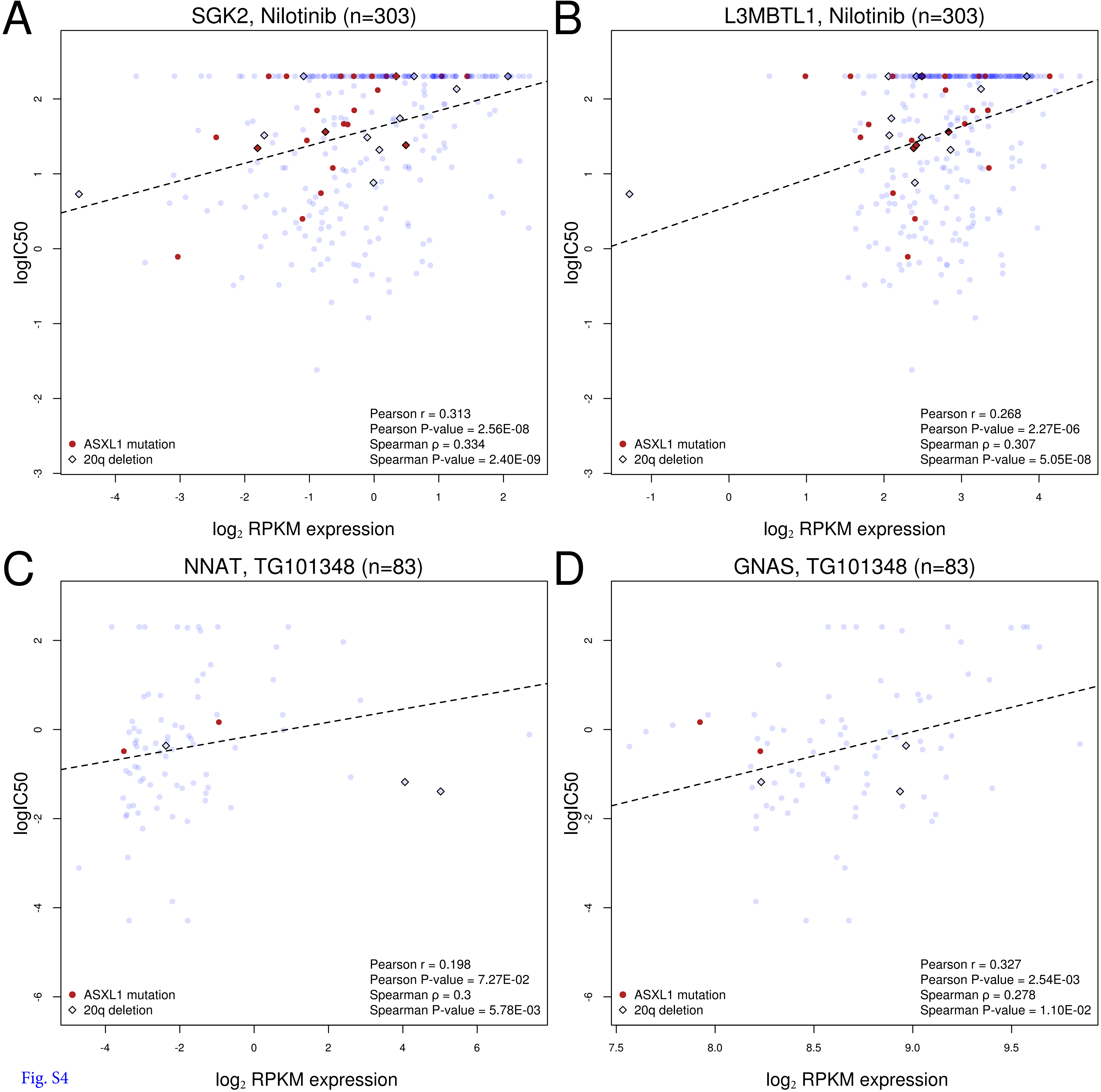
A
SGK2, Nilotinib (n=303)
Pearson r = 0.313
Pearson P-value = 2.56E-08
Spearman ρ = 0.334
Spearman P-value = 2.40E-09
ASXL1 mutation
20q deletion
logIC50
log2 RPKM expression
B
L3MBTL1, Nilotinib (n=303)
Pearson r = 0.268
Pearson P-value = 2.27E-06
Spearman ρ = 0.307
Spearman P-value = 5.05E-08
ASXL1 mutation
20q deletion
logIC50
log2 RPKM expression
C
NNAT, TG101348 (n=83)
Pearson r = 0.198
Pearson P-value = 7.27E-02
Spearman ρ = 0.3
Spearman P-value = 5.78E-03
ASXL1 mutation
20q deletion
logIC50
log2 RPKM expression
D
GNAS, TG101348 (n=83)
Pearson r = 0.327
Pearson P-value = 2.54E-03
Spearman ρ = 0.278
Spearman P-value = 1.10E-02
ASXL1 mutation
20q deletion
logIC50
log2 RPKM expression

Fig. S4

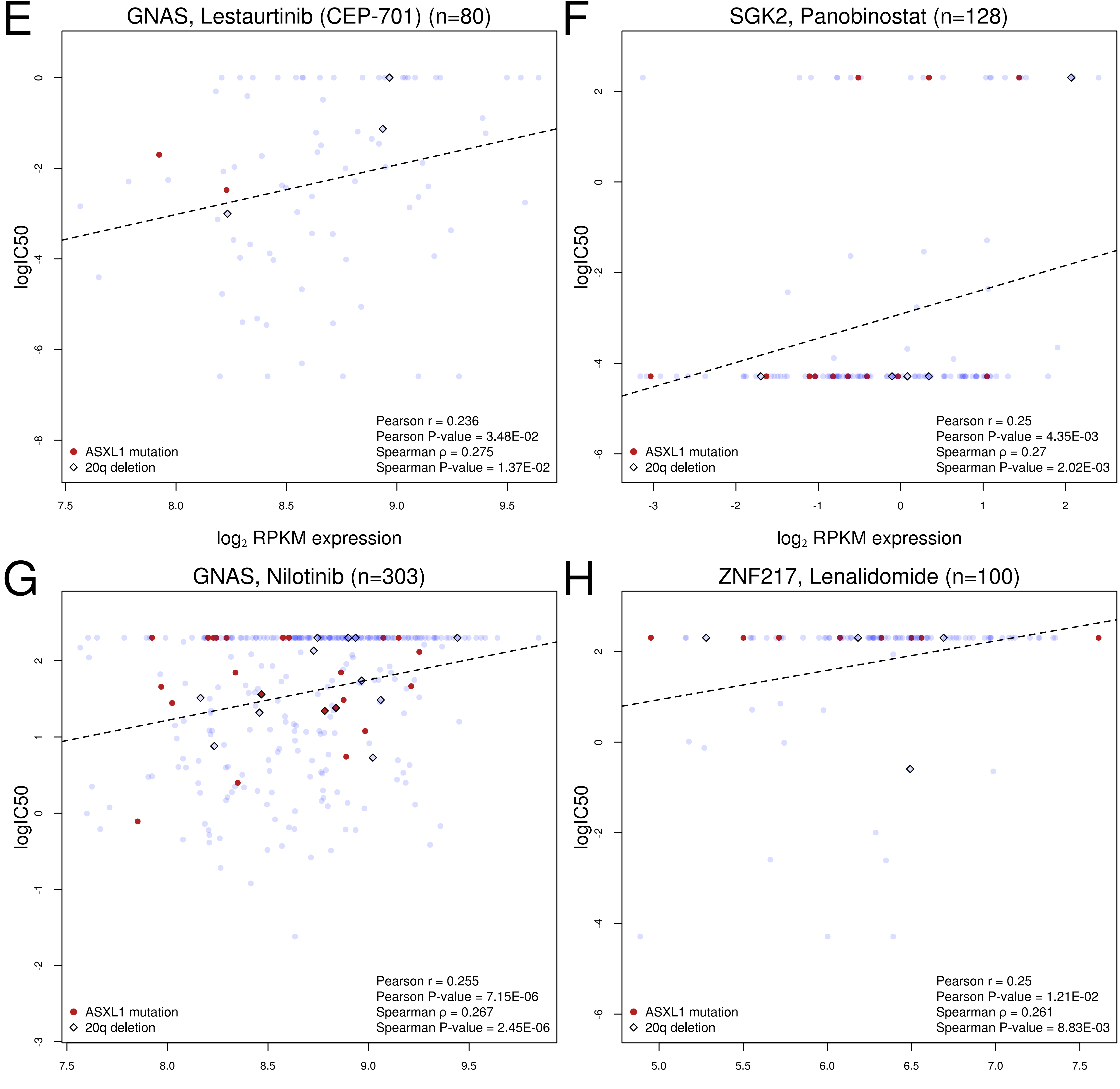

E
GNAS, Lestaurtinib (CEP-701) (n=80)
logIC50
log2 RPKM expression
ASXL1 mutation
20q deletion
Pearson r = 0.236
Pearson P-value = 3.48E-02
Spearman ρ = 0.275
Spearman P-value = 1.37E-02
F
SGK2, Panobinostat (n=128)
logIC50
log2 RPKM expression
ASXL1 mutation
20q deletion
Pearson r = 0.25
Pearson P-value = 4.35E-03
Spearman ρ = 0.27
Spearman P-value = 2.02E-03
G
GNAS, Nilotinib (n=303)
logIC50
log2 RPKM expression
ASXL1 mutation
20q deletion
Pearson r = 0.255
Pearson P-value = 7.15E-06
Spearman ρ = 0.267
Spearman P-value = 2.45E-06
H
ZNF217, Lenalidomide (n=100)
logIC50
log2 RPKM expression
ASXL1 mutation
20q deletion
Pearson r = 0.25
Pearson P-value = 1.21E-02
Spearman ρ = 0.261
Spearman P-value = 8.83E-03

Fig. S4 (cont.)

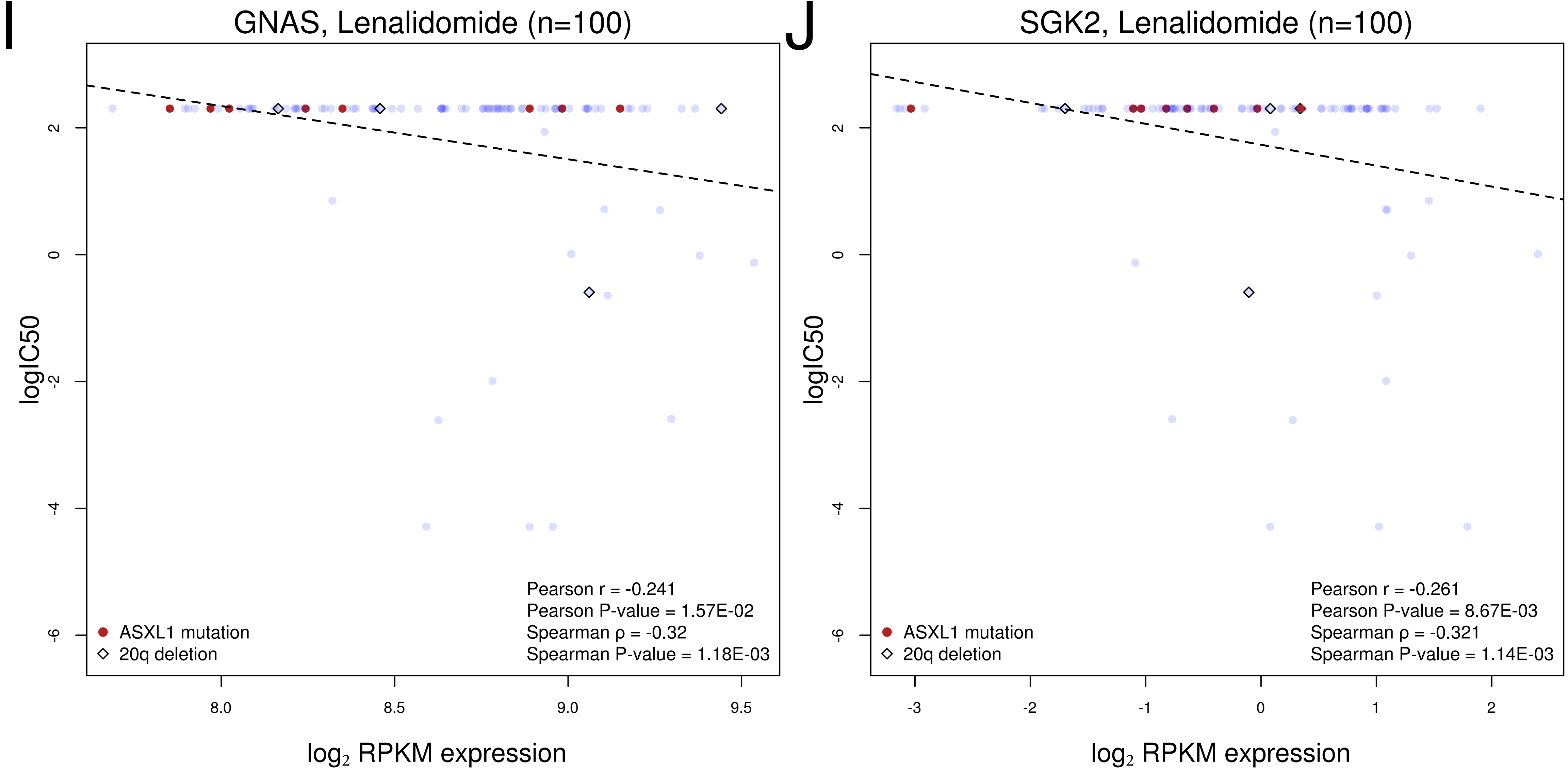

I
GNAS, Lenalidomide (n=100)
logIC50
2
0
-2
-4
-6
8.0
8.5
9.0
9.5
log2 RPKM expression
ASXL1 mutation
20q deletion
Pearson r = -0.241
Pearson P-value = 1.57E-02
Spearman ρ = -0.32
Spearman P-value = 1.18E-03
J
SGK2, Lenalidomide (n=100)
logIC50
2
0
-2
-4
-6
-3
-2
-1
0
1
2
log2 RPKM expression
ASXL1 mutation
20q deletion
Pearson r = -0.261
Pearson P-value = 8.67E-03
Spearman ρ = -0.321
Spearman P-value = 1.14E-03

Fig. S4 (cont.)
