# Supplementary figures and images for: Increased copy number of imprinted genes in the chromosomal region 20q11-q13.32 is associated with resistance to antitumor agents in cancer cell lines
Source: Clin Epigenetics. 2022 Dec 2;14:161. doi: 10.1186/s13148-022-01368-7 (PMC9716673; doi:10.1186/s13148-022-01368-7)

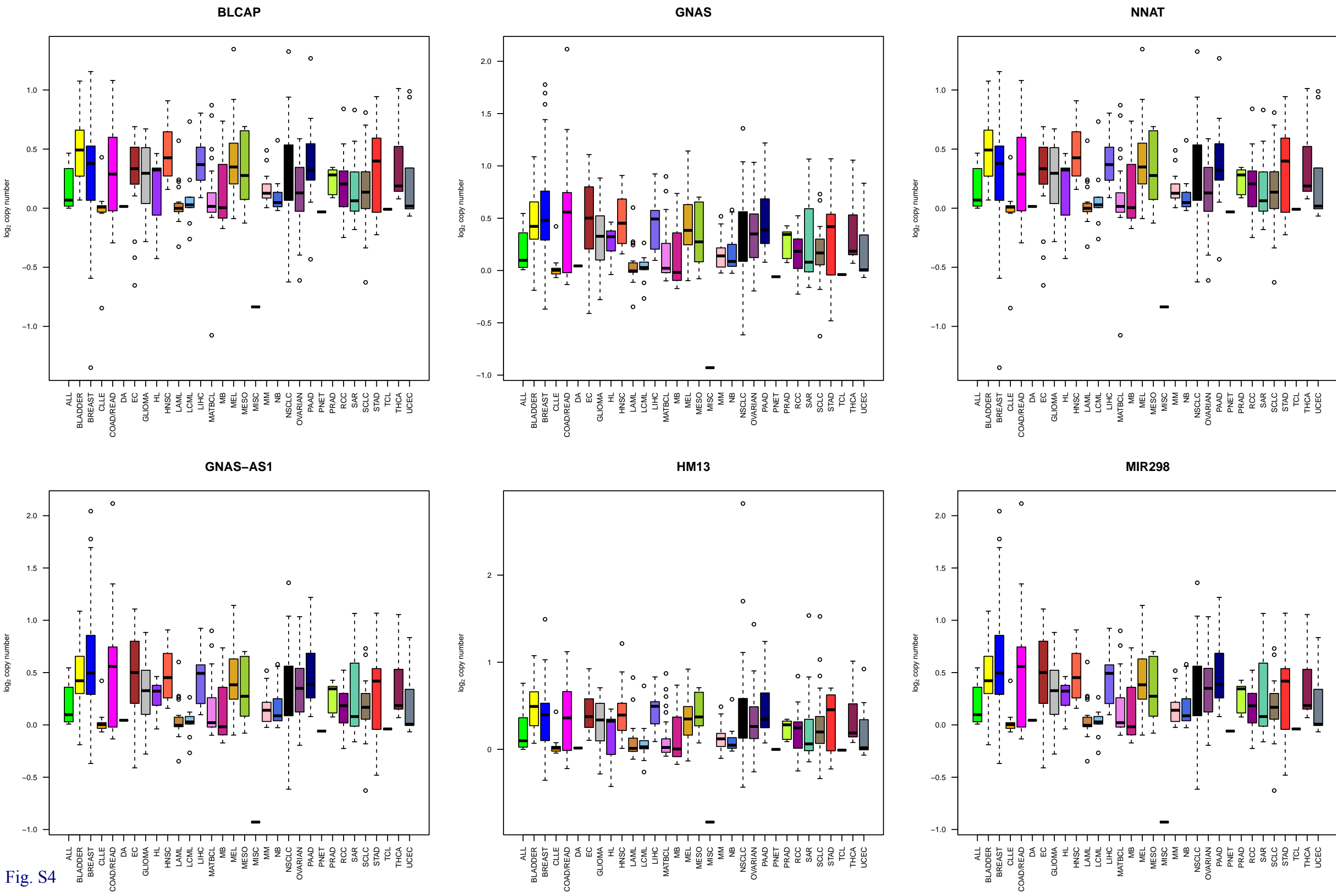

Fig. S4

Supplement: Supplementary file 13 — Additional file 13: Fig. S5. Boxplots of the distribution, by cancer category, of continuous copy number values of select genes in the 20q11-q13.32 region, for the 623 cell lines with available copy number data. [file 13148_2022_1368_MOESM13_ESM.pdf]

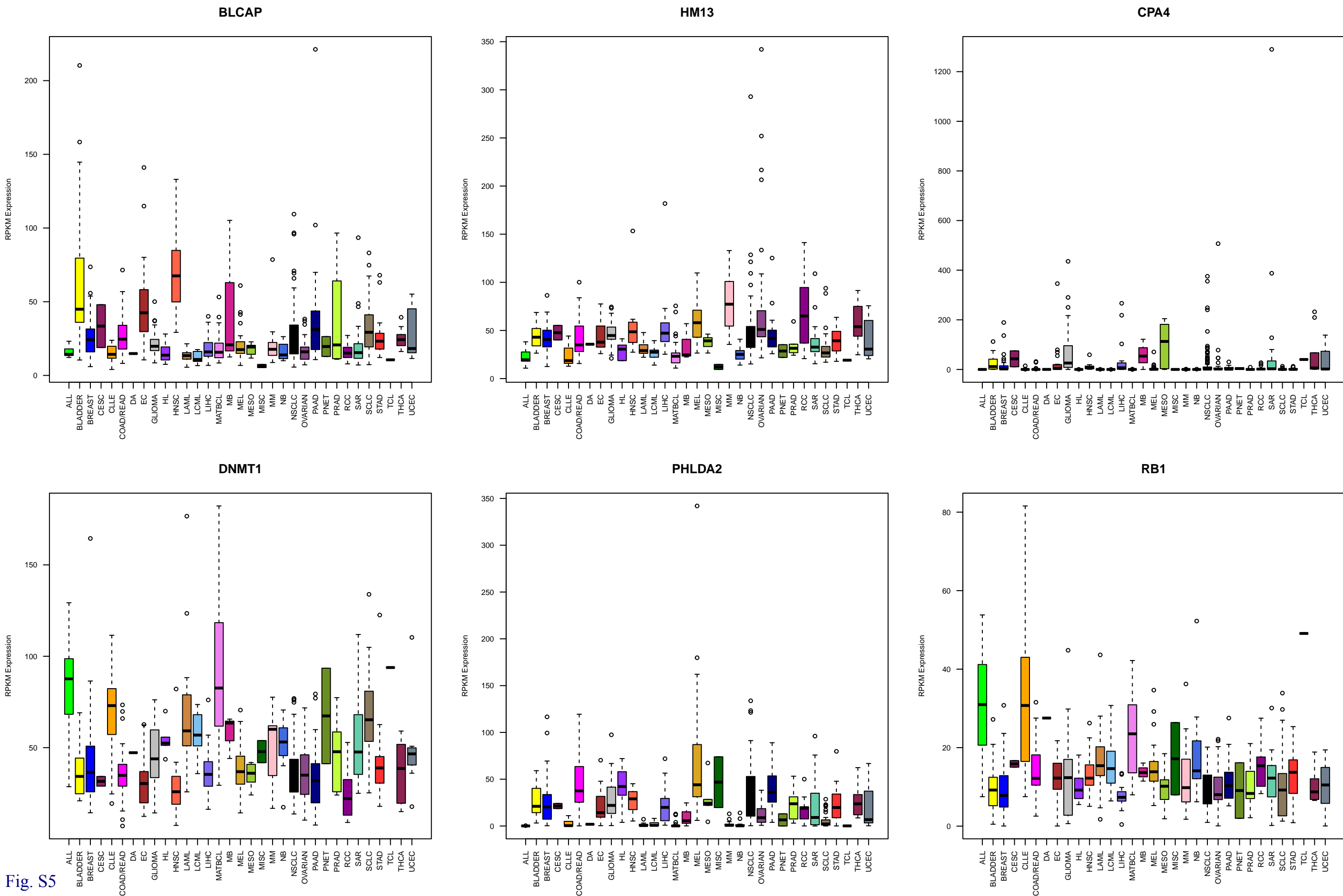

Fig. S5

Supplement: Supplementary file 14 — Additional file 14: Fig. S6. Boxplots of the distribution of gene expression measures among cancer categories in the 645 cancer cell lines. Shown are select genes from Additional file 8:Table S5 which were discussed in the text and whose expression was significantly associated drug response. BLCAP and HM13 are located in the 20q11-q13.32 region (20q11.23 and 20q11.21, respectively). The remaining genes shown in the figure are located in other chromosomal regions (CPA4 at 7q32.2, DNMT1 at 19p13.2, PHLDA2 at 11p15.4, and RB1 at 13q14.2; Additional file 2:Table S1). [file 13148_2022_1368_MOESM14_ESM.pdf]
