# Supplementary material for: Increased copy number of imprinted genes in the chromosomal region 20q11-q13.32 is associated with resistance to antitumor agents in cancer cell lines
Source: Clin Epigenetics. 2022 Dec 2;14:161. doi: 10.1186/s13148-022-01368-7 (PMC9716673; doi:10.1186/s13148-022-01368-7)
Supplement: Supplementary file 15 — Additional file 15: Fig. S7. Boxplots of the distribution of logIC50 measures of drug response among cancer categories in the 645 cancer cell lines. Shown are examples of agents from Table 1 and Additional file 8:Table S5 whose expression was significantly associated with copy number and/or expression of imprinted genes. [file 13148_2022_1368_MOESM15_ESM.pdf]

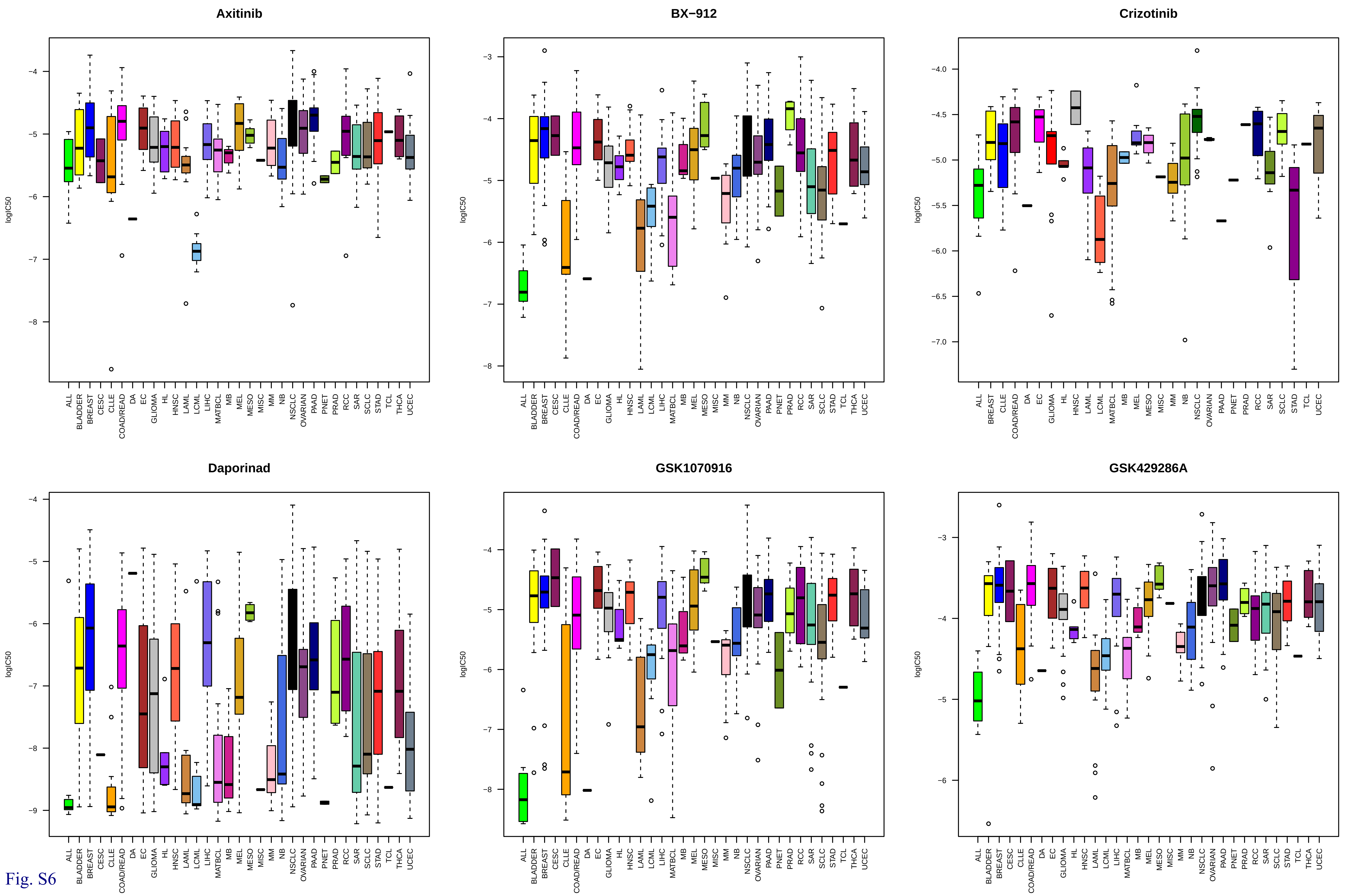
Axitinib
BX−912
Crizotinib
Daporinad
GSK1070916
GSK429286A
logIC50
ALL BLADDER BREAST CESC CLLE COAD/READ DA EC GLIOMA HL HNSC LAML LCML LIHC MATBCL MB MEL MESO MISC MM NB NSCLC OVARIAN PAAD PNET PRAD RCC SAR SCLC STAD TCL THCA UCEC
ALL BREAST CLLE COAD/READ DA EC GLIOMA HL HNSC LAML LCML MATBCL MB MEL MESO MISC MM NB NSCLC OVARIAN PAAD PNET PRAD RCC SAR SCLC STAD TCL UCEC
ALL BLADDER BREAST CESC CLLE COAD/READ DA EC GLIOMA HL HNSC LAML LCML LIHC MATBCL MB MEL MESO MISC MM NB NSCLC OVARIAN PAAD PNET PRAD RCC SAR SCLC STAD TCL THCA UCEC

Fig. S6

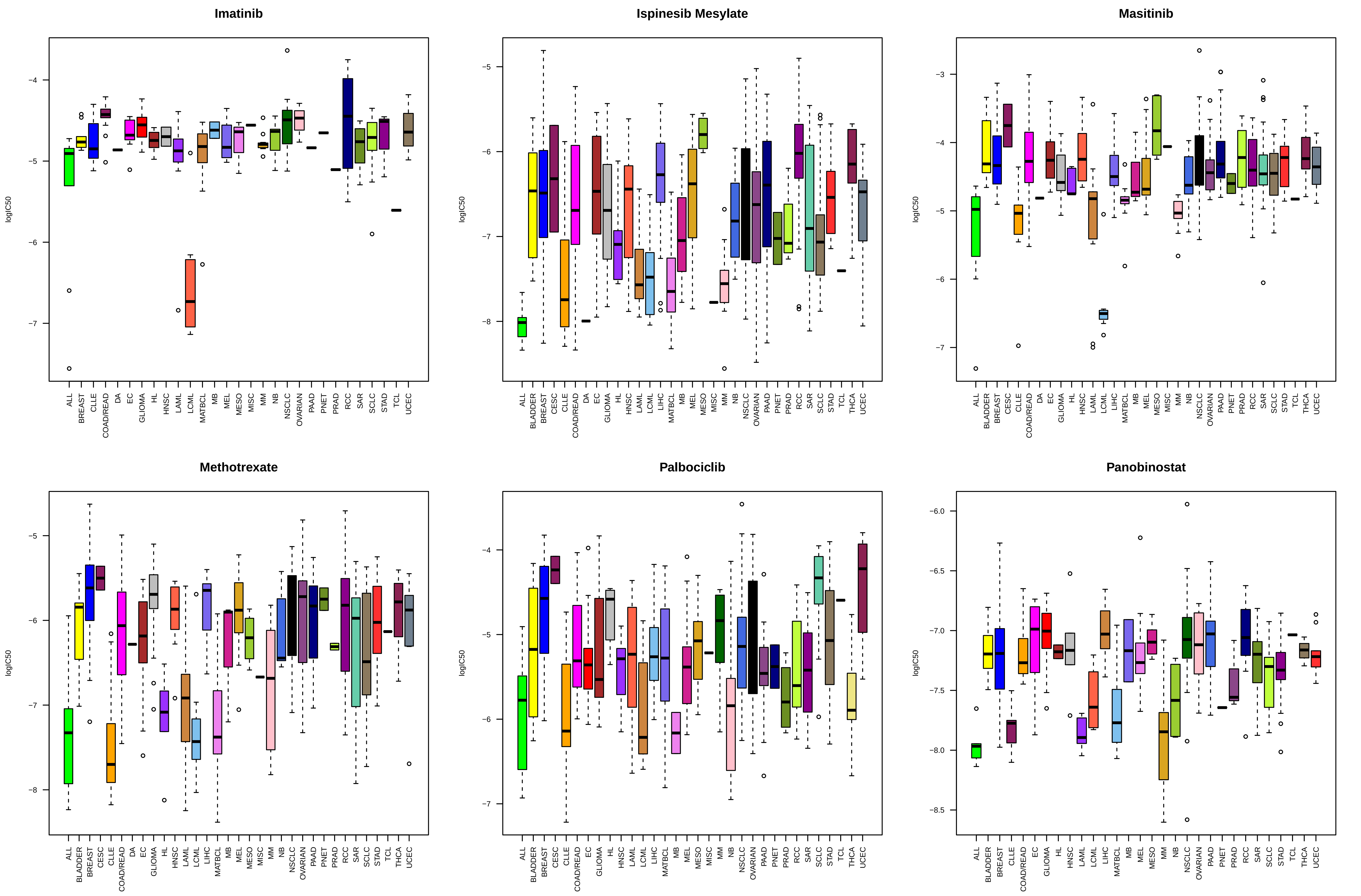
Imatinib
logIC50
-4
-5
-6
-7
ALL BREAST CLLE COAD/READ DA EC GLIOMA HL HNSC LAML LCML MATBCL MB MEL MESO MISC MM NB NSCLC OVARIAN PAAD PNET PRAD RCC SAR SCLC STAD TCL UCEC
Ispinesib Mesylate
logIC50
-5
-6
-7
-8
ALL BLADDER BREAST CESC CLLE COAD/READ DA EC GLIOMA HL HNSC LAML LCML LIHC MATBCL MB MEL MESO MISC MM NB NSCLC OVARIAN PAAD PNET PRAD RCC SAR SCLC STAD TCL THCA UCEC
Masitinib
logIC50
-3
-4
-5
-6
-7
ALL BLADDER BREAST CESC CLLE COAD/READ DA EC GLIOMA HL HNSC LAML LCML LIHC MATBCL MB MEL MESO MISC MM NB NSCLC OVARIAN PAAD PNET PRAD RCC SAR SCLC STAD TCL THCA UCEC
Methotrexate
logIC50
-5
-6
-7
-8
ALL BLADDER BREAST CESC CLLE COAD/READ DA EC GLIOMA HL HNSC LAML LCML LIHC MATBCL MB MEL MESO MISC MM NB NSCLC OVARIAN PAAD PNET PRAD RCC SAR SCLC STAD TCL THCA UCEC
Palbociclib
logIC50
-4
-5
-6
-7
ALL BLADDER BREAST CESC CLLE COAD/READ EC GLIOMA HL HNSC LAML LCML LIHC MATBCL MB MEL MESO MISC MM NB NSCLC OVARIAN PAAD PNET PRAD RCC SAR SCLC STAD TCL THCA UCEC
Panobinostat
logIC50
-6.0
-6.5
-7.0
-7.5
-8.0
-8.5
ALL BLADDER BREAST CLLE COAD/READ EC GLIOMA HL HNSC LAML LCML LIHC MATBCL MB MEL MESO MM NB NSCLC OVARIAN PAAD PNET PRAD RCC SAR SCLC STAD TCL THCA UCEC

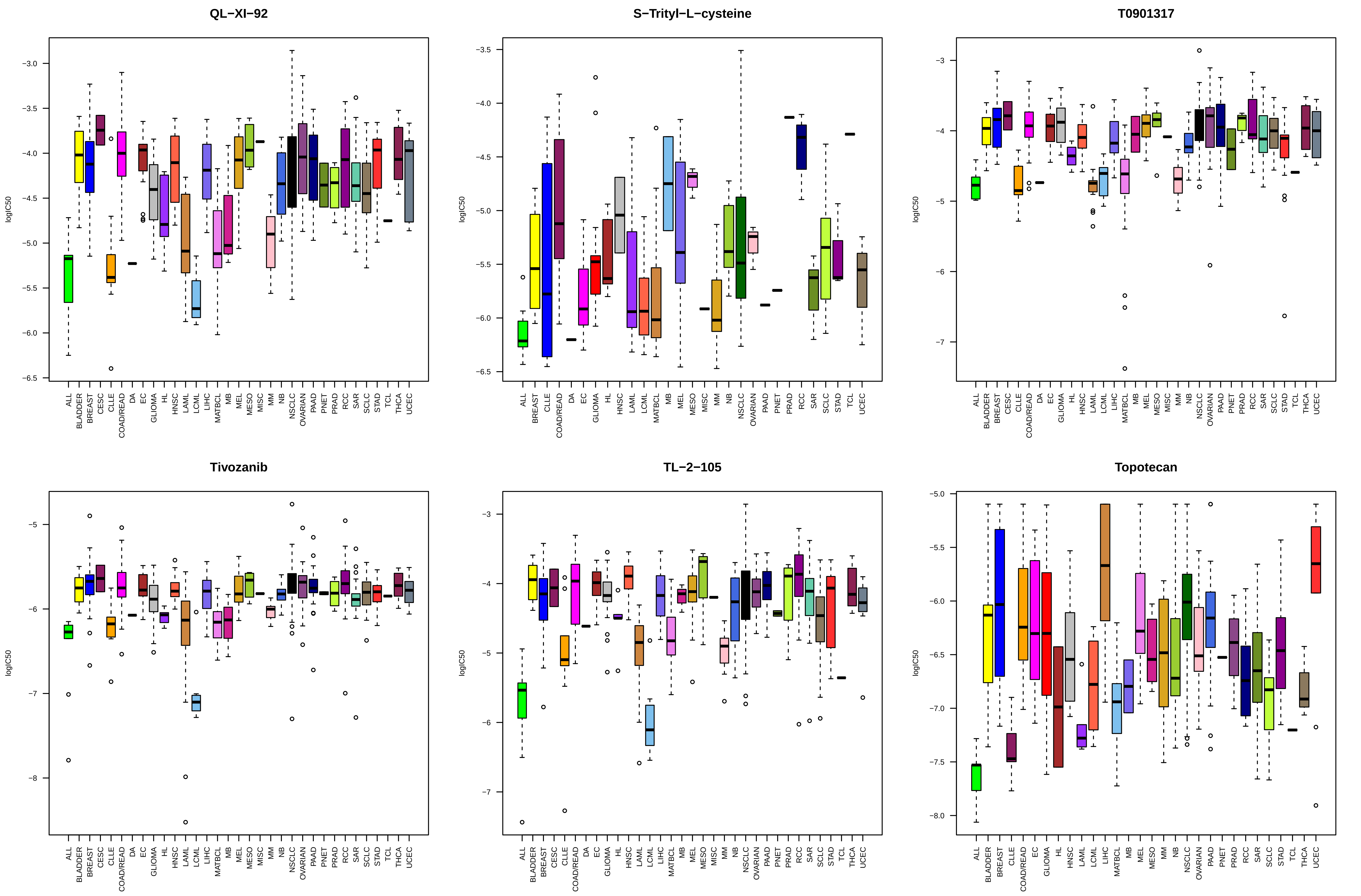
QL−XI−92
logIC50
−3.0 −3.5 −4.0 −4.5 −5.0 −5.5 −6.0 −6.5
ALL BLADDER BREAST CESC CLLE COAD/READ DA EC GLIOMA HL HNSC LAML LCML LIHC MATBCL MB MEL MESO MISC MM NB NSCLC OVARIAN PAAD PNET PRAD RCC SAR SCLC STAD TCL THCA UCEC
S−Trityl−L−cysteine
logIC50
−3.5 −4.0 −4.5 −5.0 −5.5 −6.0 −6.5
ALL BREAST CLLE COAD/READ DA EC GLIOMA HL HNSC LAML LCML MATBCL MB MEL MESO MISC MM NB NSCLC OVARIAN PAAD PNET PRAD RCC SAR SCLC STAD TCL UCEC
T0901317
logIC50
−3 −4 −5 −6 −7
ALL BLADDER BREAST CESC CLLE COAD/READ DA EC GLIOMA HL HNSC LAML LCML LIHC MATBCL MB MEL MESO MISC MM NB NSCLC OVARIAN PAAD PNET PRAD RCC SAR SCLC STAD TCL THCA UCEC
Tivozanib
logIC50
−5 −6 −7 −8
ALL BLADDER BREAST CESC CLLE COAD/READ DA EC GLIOMA HL HNSC LAML LCML LIHC MATBCL MB MEL MESO MISC MM NB NSCLC OVARIAN PAAD PNET PRAD RCC SAR SCLC STAD TCL THCA UCEC
TL−2−105
logIC50
−3 −4 −5 −6 −7
ALL BLADDER BREAST CESC CLLE COAD/READ DA EC GLIOMA HL HNSC LAML LCML LIHC MATBCL MB MEL MESO MISC MM NB NSCLC OVARIAN PAAD PNET PRAD RCC SAR SCLC STAD TCL THCA UCEC
Topotecan
logIC50
−5.0 −5.5 −6.0 −6.5 −7.0 −7.5 −8.0
ALL BLADDER BREAST CLLE COAD/READ EC GLIOMA HL HNSC LAML LCML LIHC MATBCL MB MEL MESO MM NB NSCLC OVARIAN PAAD PNET PRAD RCC SAR SCLC STAD TCL THCA UCEC

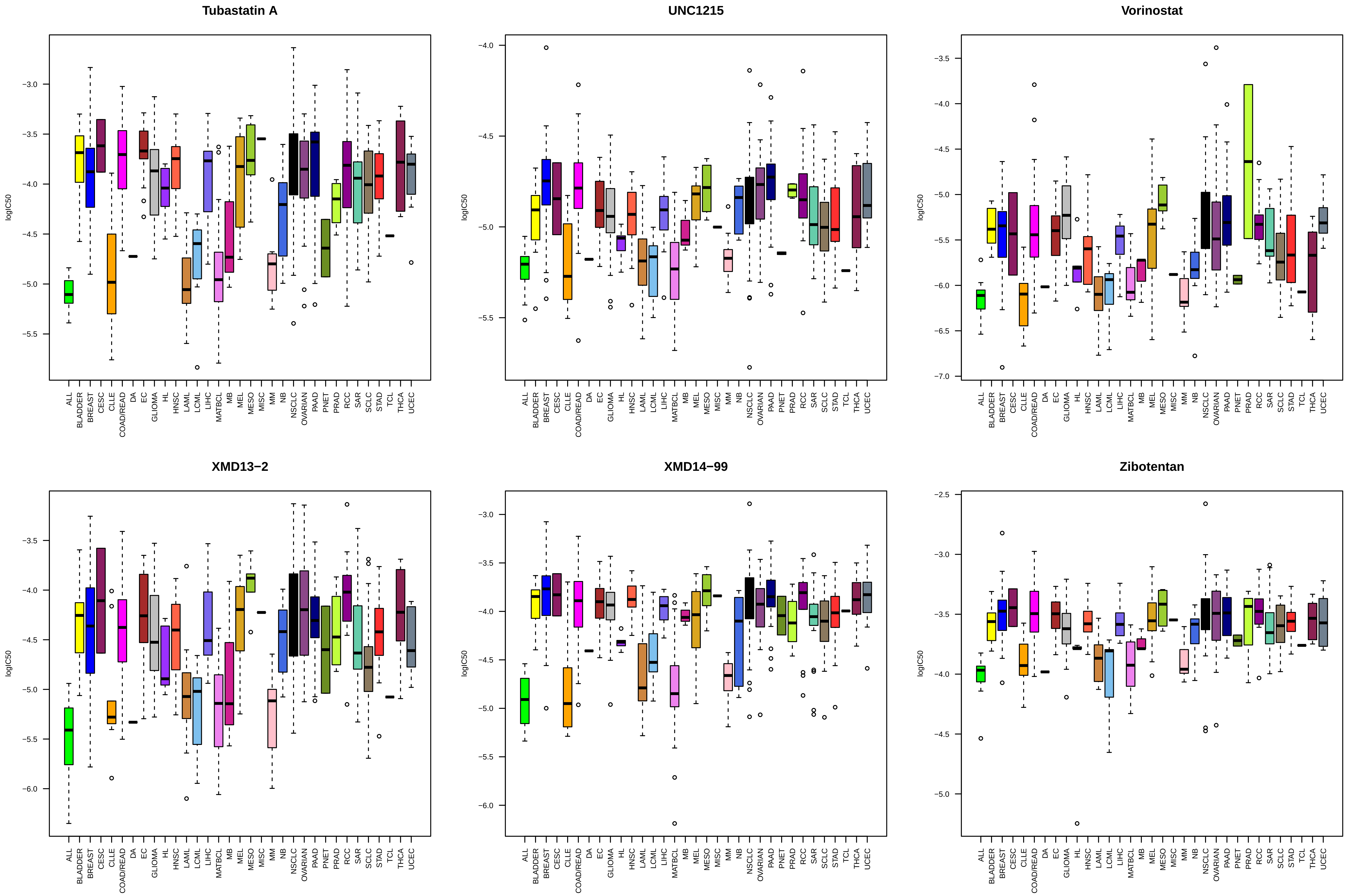
Tubastatin A
logIC50
−3.0
−3.5
−4.0
−4.5
−5.0
−5.5
UNC1215
logIC50
−4.0
−4.5
−5.0
−5.5
Vorinostat
logIC50
−3.5
−4.0
−4.5
−5.0
−5.5
−6.0
−6.5
−7.0
XMD13−2
logIC50
−3.5
−4.0
−4.5
−5.0
−5.5
−6.0
XMD14−99
logIC50
−3.0
−3.5
−4.0
−4.5
−5.0
−5.5
−6.0
Zibotentan
logIC50
−2.5
−3.0
−3.5
−4.0
−4.5
−5.0
ALL
BLADDER
BREAST
CESC
CLLE
COAD/READ
DA
EC
GLIOMA
HL
HNSC
LAML
LCML
LIHC
MATBCL
MB
MEL
MESO
MISC
MM
NB
NSCLC
OVARIAN
PAAD
PNET
PRAD
RCC
SAR
SCLC
STAD
TCL
THCA
UCEC
